# Azide–alkyne cycloaddition reactions in water *via* recyclable heterogeneous Cu catalysts: reverse phase silica gel and thermoresponsive hydrogels†

**DOI:** 10.1039/c8ra00306h

**Published:** 2018-02-06

**Authors:** Minkyung Lim, Heejin Lee, Minseok Kang, Woncheol Yoo, Hakjune Rhee

**Affiliations:** Department of Bionanotechnology, Hanyang University 55 Hanyangdaehak-ro, Sangnok-gu Ansan Gyeonggi-do 15588 South Korea.; Department of Applied Chemistry, Hanyang University 55 Hanyangdaehak-ro, Sangnok-gu Ansan Gyeonggi-do 15588 South Korea hrhee@hanyang.ac.kr

## Abstract

Functionalized reverse phase silica gel and thermoresponsive hydrogels were synthesized as heterogeneous catalysts supports. Cu(i) and Cu(ii) catalysts immobilized onto two types of supports were prepared and characterized. The copper catalyzed azide–alkyne cycloaddition was performed in water *via* a one-pot reaction and yielded good results. These catalysts are air stable and reusable over multiple uses.

## Abstract 2

Azide–alkyne cycloaddition reactions were performed *via* copper catalysts immobilized onto two types of supports in water.

## Graphical abstract

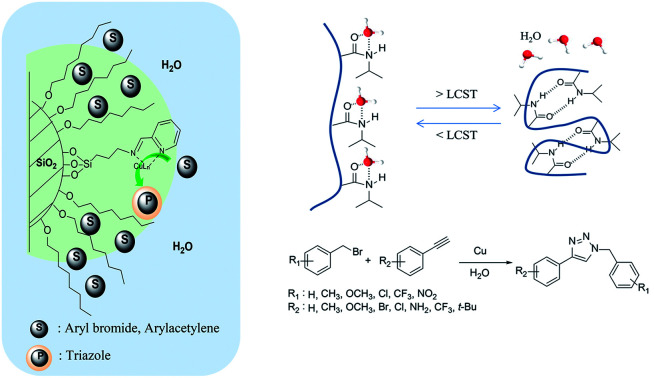

## 1. Introduction

Triazole is a very useful motif within the fields of medicinal chemistry,^1–4^ carbohydrate chemistry,^5–7^ and materials science.^8–12^ Triazole has also been studied as a strong ligand for metal coordination with wide applicability.^13–16^ The thermal Huisgen 1,3-dipolar cycloaddition of organoazides and alkynes is a classic method for the synthesis of triazole.^17,18^ However, this method is slow even at high temperatures, producing a mixture of 1,4- and 1,5-disubstituted triazoles. Numerous synthetic methods using metal catalysts have been reported;^19–29^ copper has been shown to especially accelerate the reaction. The copper(i)-catalyzed azide–alkyne cycloaddition (CuAAC) reaction initiated by Sharpless^30^ and Meldal^31^ is considered to be a powerful pathway with a high regioselectivity and yield. However, copper catalyzed methods also possess drawbacks due to limited catalyst recyclability, the use of organic solvents, and the use of relatively expensive copper complexes that are difficult to remove.^32–35^ Therefore, the development of copper immobilized heterogeneous catalysts has attracted attention due to its reusability and ease of catalyst separation.^36–41^ A variety of solid supports have been applied including zeolites,^42^ charcoal,^43,44^ silica,^45,46^ and polysaccharides.^47^ Additionally, the use of water as a solvent is a promising approach with regard to green chemistry.^37,38,48–52^

Recently, we developed two types of solid supports. One is an aminopropyl-functionalized reverse phase silica gel, which is end capped with a hydrophobic alkyl group. The other one is a thermoresponsive poly(*N*-isopropylacrylamide-*co*-4-vinylpyridine) (pNIPAM-VP) exhibiting hydrophilicity and hydrophobicity depending on temperature. These properties make it possible to use water as a solvent for organic reactions. These solid supports were applied to the syntheses of Pd, Au immobilized heterogeneous catalysts and organic reactions such as hydrogenations,^53^ Suzuki–Miyaura couplings,^54,55^ Heck–Mizoroki couplings,^56^ Sonogashira couplings,^56^ Tsuji–Trost reactions,^57^ and A-3 coupling reactions^58^ that were performed in water. Herein, we wish to demonstrate that our supports can be extended to other metal catalyzed organic reaction applications. In this study, Cu(i) and Cu(ii) catalysts immobilized onto two support types were synthesized and characterized. The copper catalyzed azide–alkyne cycloaddition was performed in water.

## 2. Results and discussion

### 2.1. Copper catalysts immobilized onto reverse phase silica gel

#### Synthesis and characterization

**Scheme 1:**
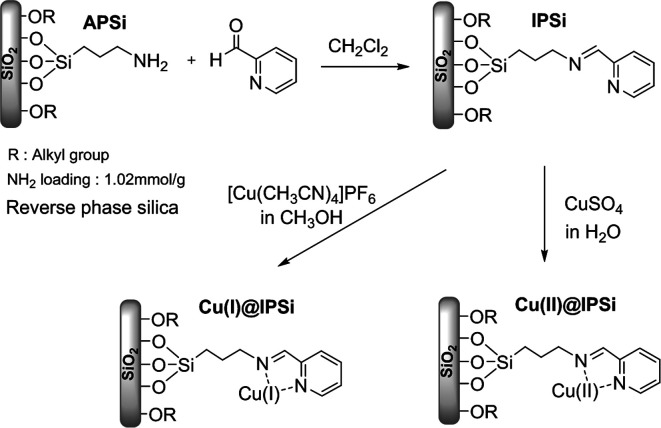
Synthesis of Cu(i)@IPSi and Cu(ii)@IPSi.

**Fig. 1:**
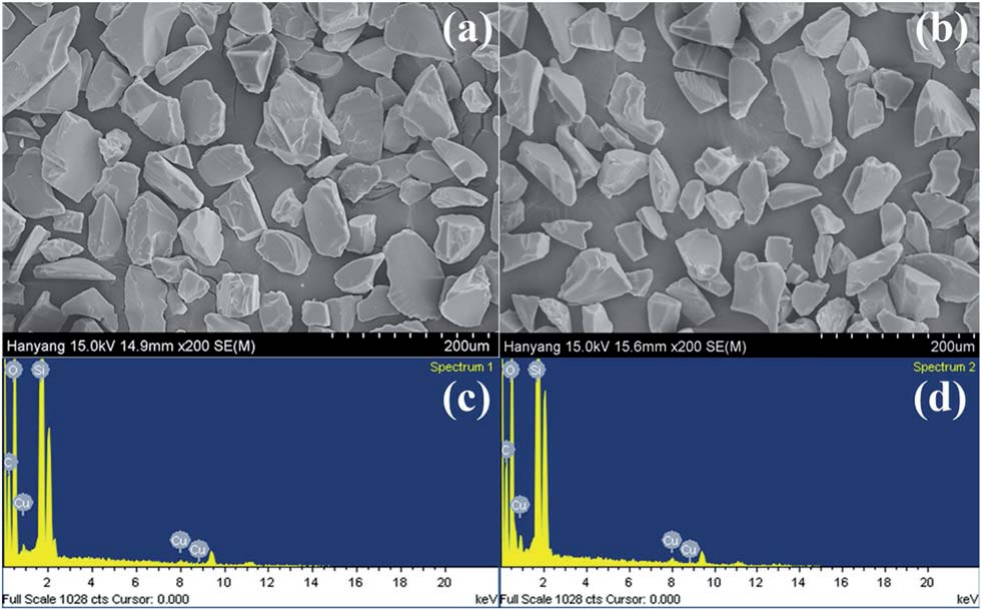
Scanning electron microscope (SEM) image and energy dispersive X-ray analysis (EDXA) of Cu(i)@IPSi (a, c) and Cu(ii)@IPSi (b, d).

Cu(i) and Cu(ii) catalysts immobilized onto reverse phase silica gel were synthesized; the preparation methods are outlined in Scheme 1. First, the 2-pyridinecarboxaldehyde ligand was anchored to a commercially available reverse phase 3-aminopropyl-functionalized silica gel (APSi). The complexation of [Cu(CH_3_CN)_4_]PF_6_ and CuSO_4_ with the resulting iminopropyl-functionalized silica gel (IPSi) subsequently followed, yielding Cu(i)@IPSi and Cu(ii)@IPSi. Introduction of the 2-pyridinecarboxaldehyde ligand to the starting silica gel was confirmed *via* solid-state ^13^C NMR (ESI). The copper catalysts possessed an irregular shape based on scanning electron microscopy (SEM) images acquired primarily from the shape of the starting silica gel. Energy dispersive X-ray analysis (EDXA) further confirmed that Cu was indeed present (Fig. 1). Copper loading values on the catalysts were determined *via* inductively coupled plasma (ICP); the results showed the loading values to be 0.498 mmol g^−1^ of Cu(i)@IPSi and 0.319 mmol g^−1^ of Cu(ii)@IPSi. As a result of the Brunnauer–Emmet–Teller (BET) method, the catalysts possessed pore sizes of 6–8 nm similar to the starting silica gel and pore volumes of 0.332 cm^3^ g^−1^ and 0.416 cm^3^ g^−1^ for Cu(i)@IPSi and Cu(ii)@Si, respectively (ESI). The oxidation number of copper supported on the catalyst was confirmed by X-ray photoelectron spectroscopy (XPS) (ESI).

**Fig. 2:**
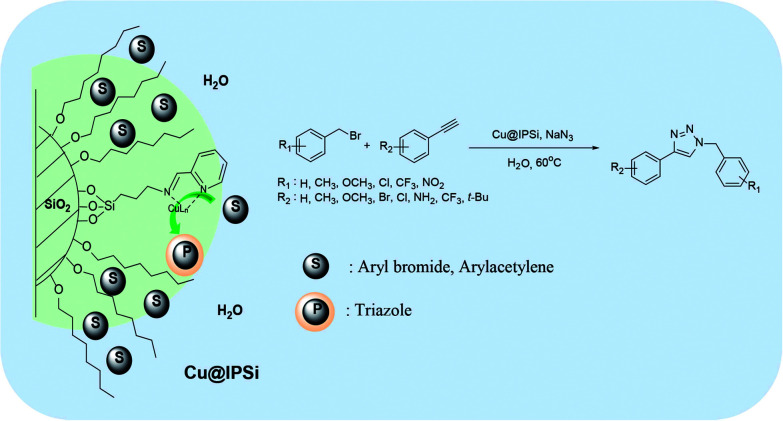
Catalytic behavior of the reverse phase silica gel in water.

The reverse phase silica gel possessed an end capped alkyl group yielding hydrophobicity. We anticipated that transport of the hydrophobic substrate towards the hydrophobic surface would enhanced reactivity by bringing the substrate and catalyst in close proximity.^47^ This property would also enable the azide–alkyne cycloaddition reaction in water (Fig. 2).

#### Azide–alkyne cycloaddition

Azide–alkyne cycloaddition reactions were performed to explore the catalytic activity of the prepared catalysts. The reaction was performed *via* the one-pot reaction of benzyl bromide, sodium azide, and phenylacetylene. First, the reaction of benzyl bromide with sodium azide generated benzyl azide *in situ*. Then, benzyl azide reacted with phenylacetylene to form the triazole product. Optimization of the reaction has been assessed as shown in Table 1. The turnover number (TON) and turnover frequency (TOF) values were compared. Initially, the reaction was performed at room temperature using 5 mol% of catalyst with excellent yields (94%) by the Cu(i)@IPSi and Cu(ii)@IPSi catalysts (Table 1, entries 1 & 6). The reaction temperature was varied to reduce the reaction time and the amount of catalyst was reduced to improve the TON and TOF values. The reactivity of the copper catalyst immobilized on the starting silica gel (Cu@APSi) was also compared to countercheck effects of the ligand (Table 1, entries 4 & 5, 7 & 10).

**Table 1:** Optimization of the azide–alkyne cycloaddition^a^

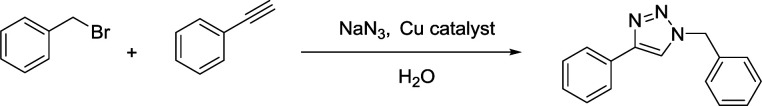							
Entry	Catalyst	Mol%	Temp. (°C)	Time (h)	Yield^b^ (%)	TON^c^ (mol mol^−1^)	TOF^d^ (h^−1^)
1	Cu(i)@IPSi	5	r.t.	24	94	18.8	0.78
2	Cu(i)@IPSi	5	60	2	92	18.4	9.2
3	Cu(i)@IPSi	5	80	2	89	17.8	8.9
**4**	**Cu(** **i** **)@IPSi**	**2.5**	**60**	**2**	**92**	**36.8**	**18.4**
5	Cu(i)@APSi	2.5	60	2	84	35.2	17.6
6	Cu(ii)@IPSi	5	r.t.	24	94	18.8	0.78
**7**	**Cu(** **ii** **)@IPSi**	**5**	**60**	**2**	**93**	**18.6**	**9.3**
8	Cu(ii)@IPSi	5	80	2	87	17.4	8.7
9	Cu(ii)@IPSi	2.5	60	2	87	34.8	17.4
10	Cu(ii)@APSi	5	60	2	78	15.6	7.8

a Reaction conditions: benzyl bromide (1.0 mmol), phenyl acetylene (1.0 mmol), sodium azide (1.0 mmol), H_2_O (2 mL).

b Isolated yield.

c TON (turnover number): reactant moles converted/catalyst moles.

d TOF (turnover frequency): TON/reaction time.

We considered various substrates for further investigation as can be seen in Table 2. The azide–alkyne cycloaddition was performed under the established reaction conditions of 60 °C in water (Table 1, 4 & 7). Both catalysts were effective and Cu(i)@IPSi was more reactive than Cu(ii)@IPSi. The reaction proceeded smoothly in water, an environmentally friendly solvent. All reactants, aryl bromides and arylacetylenes regardless of attached electron withdrawing and electron donating substituents, yielded good results (74%-quant. yield).

**Table 2:** Substrate scope using Cu@IPSi

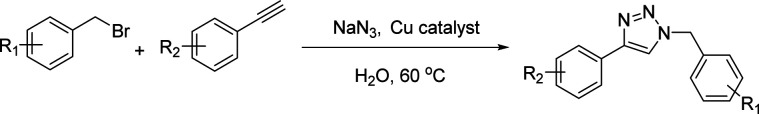											
Entry	Product	Cu(i)@IPSi^a^		Cu(ii)@IPSi^b^		Entry	Product	Cu(i)@IPSi^a^		Cu(ii)@IPSi^b^	
		Time (h)	Isolated yield (%)	Time (h)	Isolated yield (%)			Time (h)	Isolated yield (%)	Time (h)	Isolated yield (%)
1	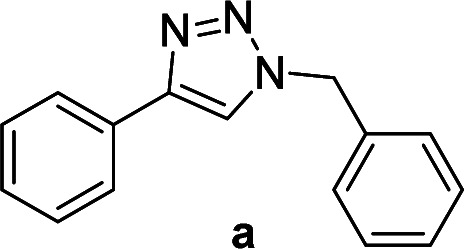	2	92	2	93	10^d^	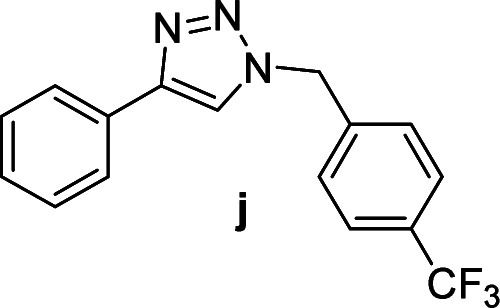	6	80	6	83
2	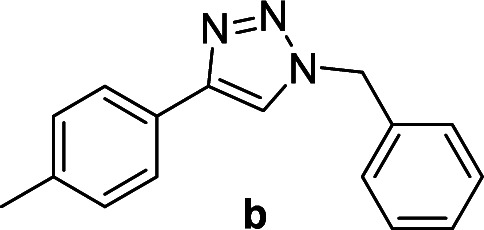	2	93	2	90	11	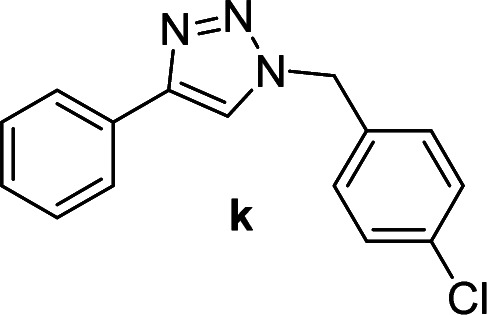	4	90	4	90
3	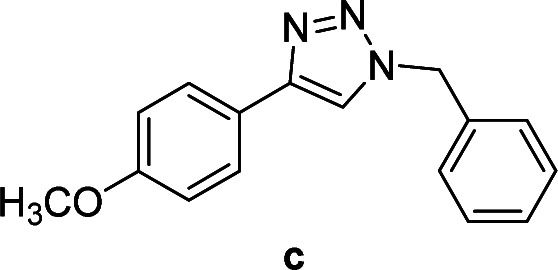	2	Quant.	2	88	12	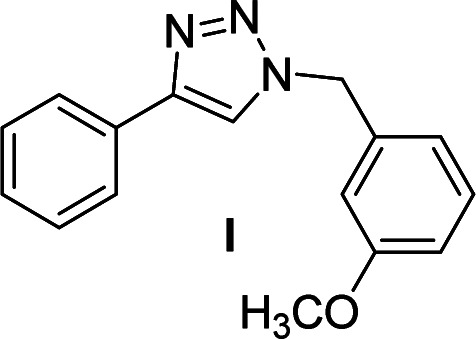	6	96	6	96
4	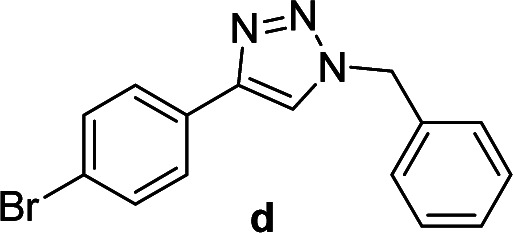	2	97	2	91	13^d^	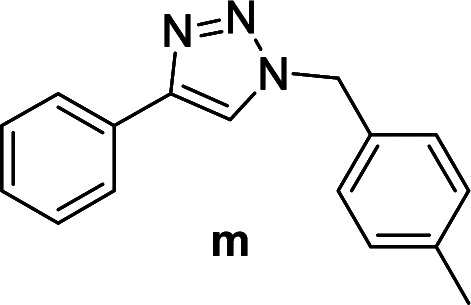	4	84	4	84
5^c^	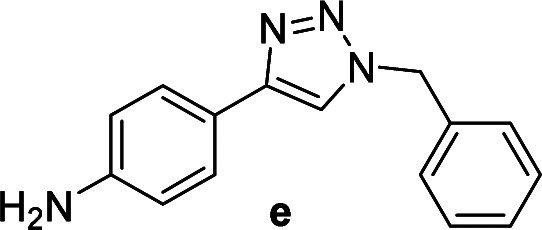	4	76	4	74	14	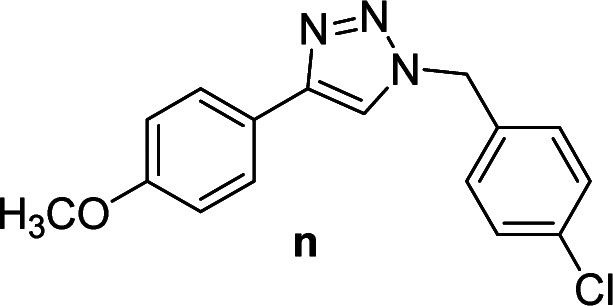	4	91	6	91
6	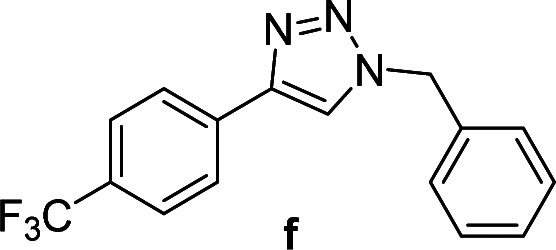	2	90	2	90	15	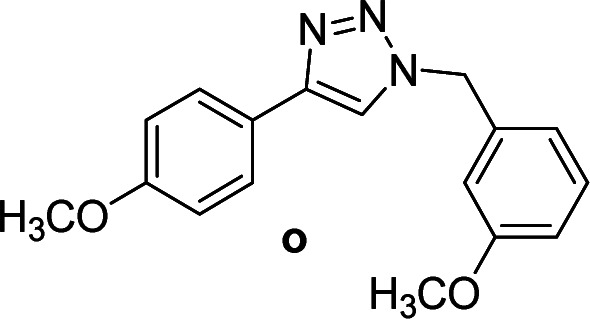	6	90	6	93
7	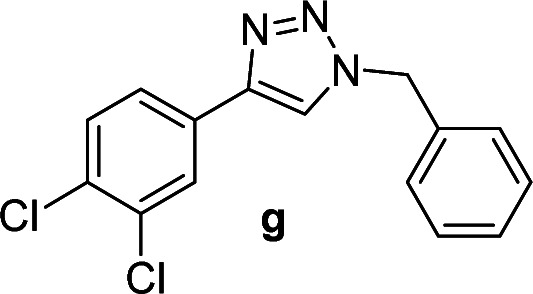	2	96	2	88	16	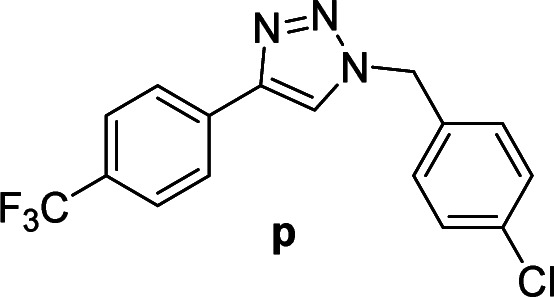	6	95	6	94
8	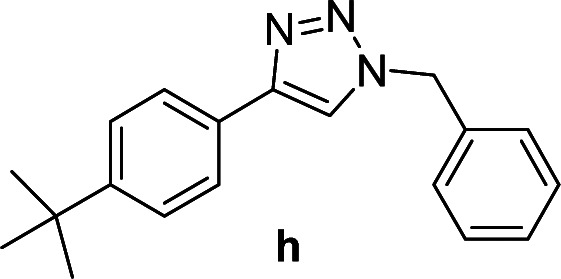	2	96	2	86	17	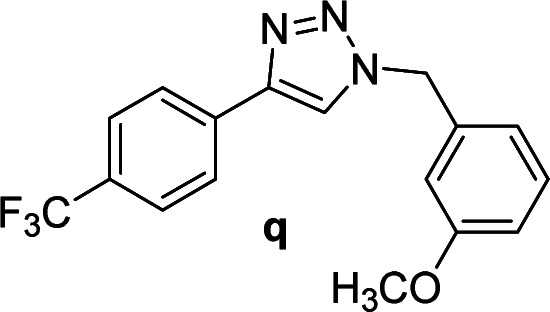	6	97	6	86
9	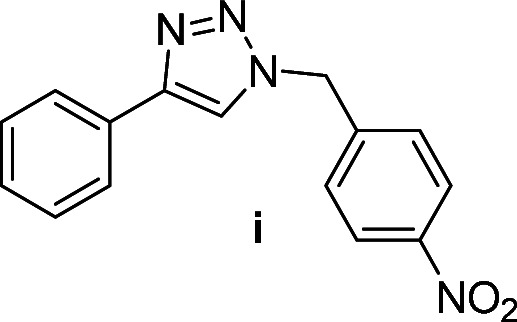	6	94	6	94						

a Reaction conditions: aryl bromide (1.0 mmol), aryl acetylene (1.0 mmol), sodium azide (1.0 mmol), Cu(i)@IPSi (2.5 mol% of Cu), H_2_O (2 mL).

b Cu(ii)@IPSi (5.0 mol% of Cu).

c 4-Aminobenzyl azide was prepared by the reaction of 4-aminobenzyl bromide with sodium azide for 2 hours; phenyl acetylene was subsequently added to the reaction mixture.

d Benzylbromide (1.2 mmol).

#### Recycling test

**Fig. 3:**
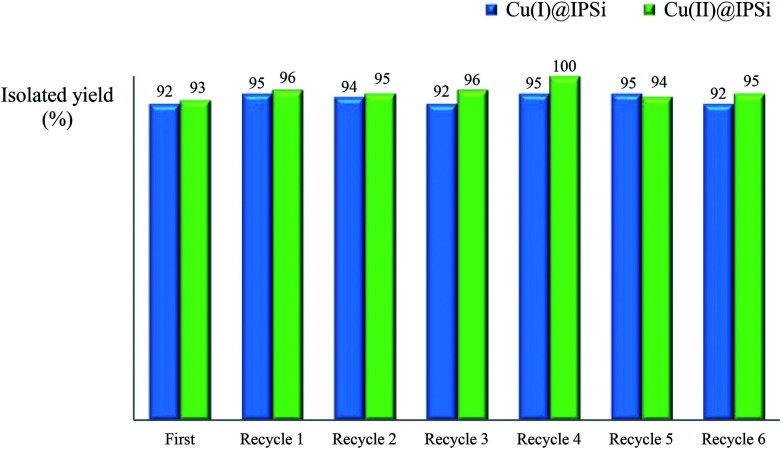
Recycling test of Cu(i)@IPSi and Cu(ii)@IPSi.

Recycling tests were performed using recovered catalysts under the established reaction conditions of 60 °C in water (Table 1, entry 4 & 9). As can be seen in Fig. 3, both catalysts were reusable several times, exhibiting little to no change to the product yield (92%-quant.). Since there was a slight difference in the amount of catalyst recovered in each reaction, a difference in yield was inevitable. After the reaction, catalysts were collected by hot filtration and the filtrate was checked *via* ICP to confirm the leaching of copper.

Fortunately, the ICP data showed no significant copper loss from both catalysts. XPS analysis was performed to confirm the oxidation state of freshly prepared catalyst and recovered catalyst (more details, see ESI†).

### 2.2. Copper catalyst immobilized on thermoresponsive hydrogels

#### Synthesis and characterization

**Scheme 2:**
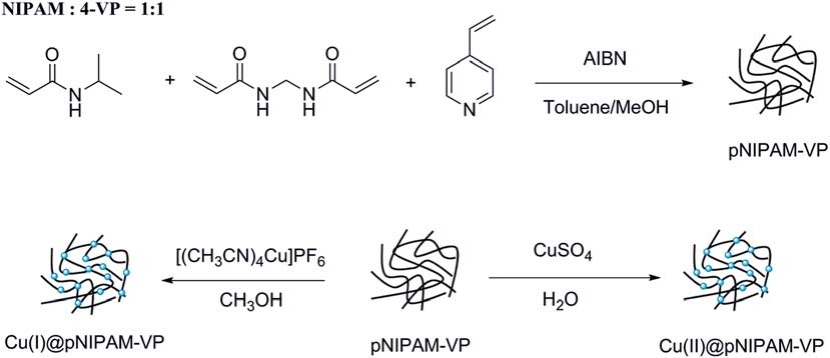
Synthesis of Cu(i)@pNIPAM-VP and Cu(ii)@pNIPAM-VP.

**Fig. 4:**
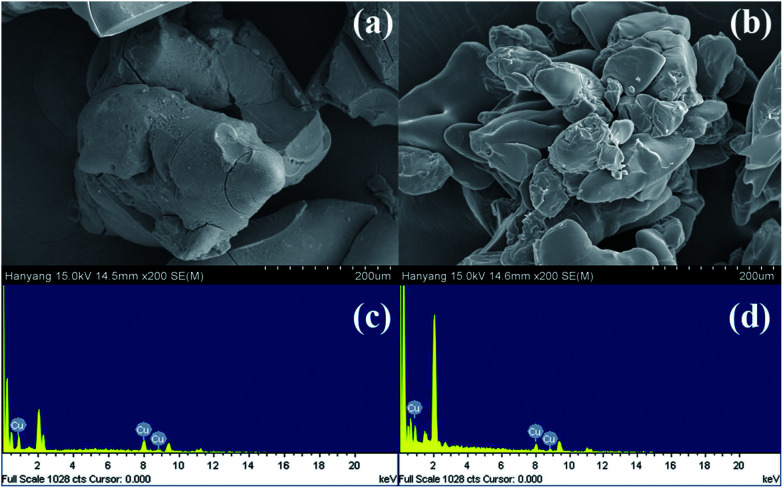
Scanning electron microscope (SEM) images and energy dispersive X-ray analysis (EDXA) of Cu(i)@pNIPAM-VP (a, c) and Cu(ii)@pNIPAM-VP (b, d).

Poly(*N*-isopropylacrylamide-*co*-4-vinylpyridine) (pNIPAM-VP) hydrogels were prepared *via* free radical solution polymerization using *N*-isopropylacrylamide (NIPAM), *N*,*N*′-methylenebis-acrylamide (MBAAm), and 4-vinylpyridine (4-VP) according to a previously reported procedure.^55^ As outlined in Scheme 2, copper coordination by [Cu(CH_3_CN)_4_]PF_6_ and CuSO_4_ was followed. The morphology of the resulting catalysts Cu(i)@pNIPAM-VP and Cu(ii)@pNIPAM-VP was determined *via* SEM imaging. The presence of copper in the catalyst was also confirmed *via* EDXA (Fig. 4). The oxidation number of copper supported on the catalyst was confirmed by X-ray photoelectron spectroscopy (XPS) (ESI).

**Fig. 5:**
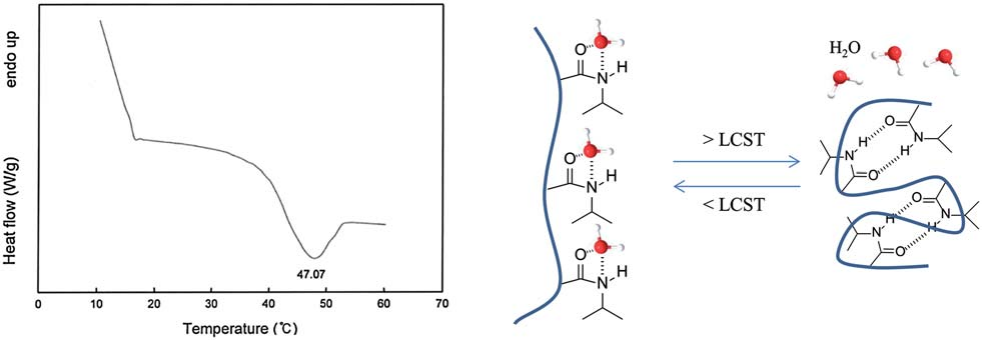
Differential scanning calorimetry curve for pNIPAM-VP.

The loading values of copper were determined *via* ICP and the results revealed loading levels of 0.374 mmol g^−1^ for Cu(i)@pNIPAM-VP and 0.166 mmol g^−1^ for Cu(ii)@pNIPAM-VP. pNIPAM-VP supports featured a special low critical solution temperature (LCST) that imparted additional control to accelerate the reaction. According to the differential scanning calorimetry (DSC) data, the LCST of the synthesized polymer was 47 °C, as can be seen in Fig. 5. At temperatures higher than 47 °C, hydrogen bonds between the polymer and solvent molecules become weak and the polymer coils around itself, shrinking in size as intramolecular hydrogen bonding becomes significant.^59,60^ This mechanism was proposed for the coil-to-globule transition of polymer coils that took place in hydrophilic solvents (Fig. 5).

### 2.3. Azide–alkyne cycloaddition

One-pot azide–alkyne cycloaddition reactions were performed to explore the catalytic activity of the prepared catalysts. Initially, we chose benzyl bromide and phenylacetylene as substrates and the reaction was performed at 60 °C above the LCST (47 °C). The reaction was attempted by varying the quantity of catalyst and the TON and TOF values of the corresponding reaction were compared as can be seen in Table 3. Cu(ii)@pNIPAM-VP (1 mol%) was able to effectively perform the reaction (91%). In case of Cu(i)@pNIPAM-VP (5 mol%), the product yield was 94% (Table 3, entries 1 & 4).

**Table 3:** Optimization of the azide–alkyne cycloaddition^a^

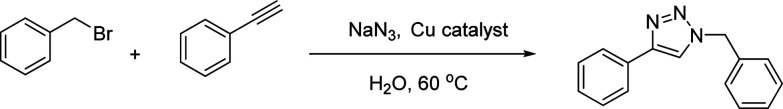						
Entry	Catalyst	Mol%	Time (h)	Yield^b^ (%)	TON^c^ (mol mol^−1^)	TOF^d^ (h^−1^)
**1**	**Cu(** **i** **)@pNIPAM-VP**	**5**	**2**	**94**	**18.8**	**9.4**
2	Cu(i)@pNIPAM-VP	2	2	89	44.5	22.3
3	Cu(i)@pNIPAM-VP	1	2	62	62	31
**4**	**Cu(** **ii** **)@pNIPAM-VP**	**1**	**2**	**91**	91	**45.5**

a Reaction conditions: benzyl bromide (1.0 mmol), phenyl acetylene (1.0 mmol), sodium azide (1.0 mmol), H_2_O (2 mL).

b Isolated yield.

c TON (turnover number): reactant moles converted/catalyst moles.

d TOF (turnover frequency): TON/reaction time.

The azide–alkyne cycloaddition was performed using various substrates with electron withdrawing and electron donating substituents (Table 4). Reactions with both catalysts proceeded in water and Cu(ii)@pNIPAM-VP was more reactive than Cu(i)@pNIPAM-VP.

**Table 4:** Substrate scope using Cu@pNIPAM-VP

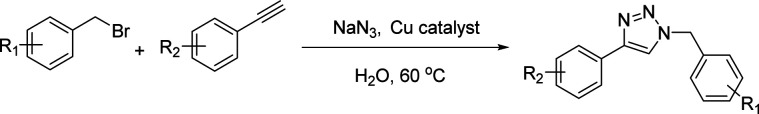											
Entry	Product	Cu(i)@pNIPAM-VP^a^		Cu(ii)@pNIPAM-VP^b^		Entry	Product	Cu(i)@pNIPAM-VP^a^		Cu(ii)@pNIPAM-VP^b^	
		Time (h)	Isolated yield (%)	Time (h)	Isolated yield (%)			Time (h)	Isolated yield (%)	Time (h)	Isolated yield (%)
1	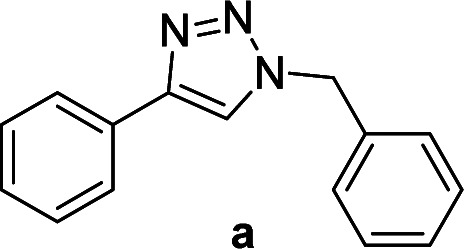	2	94	2	91	10	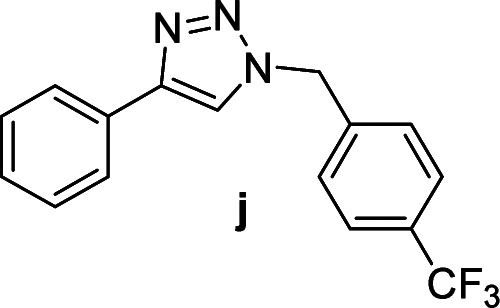	6	88	8	75
2	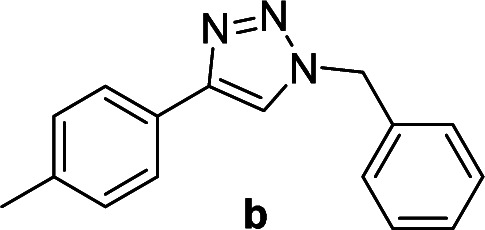	3	92	2	93	11^d^	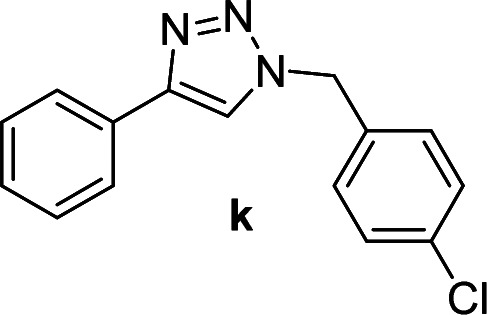	4	62	4	88
3	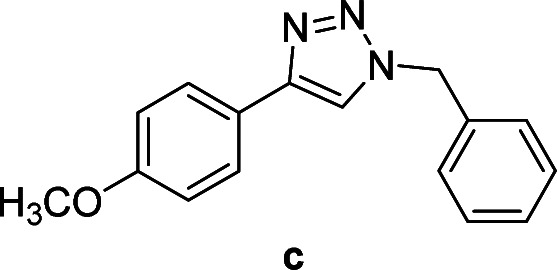	2	92	2	92	12	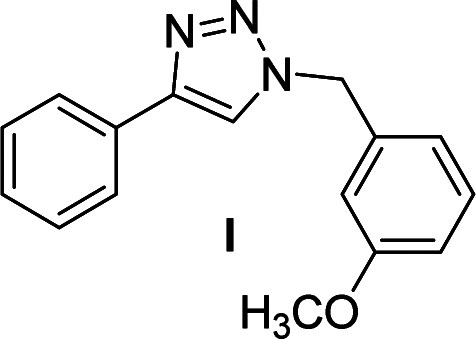	6	89	6	88
4	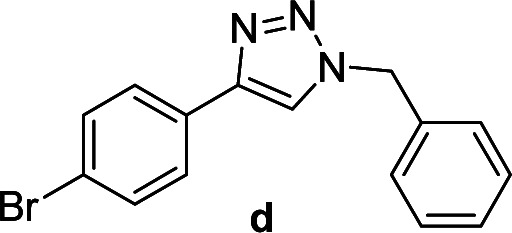	4	93	4	92	13^d^	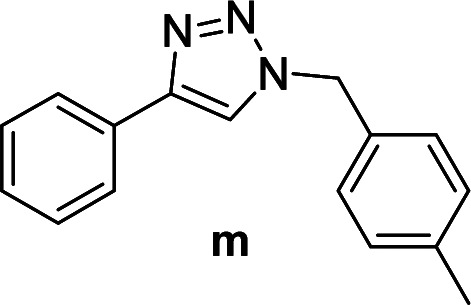	6	70	6	73
5^c^	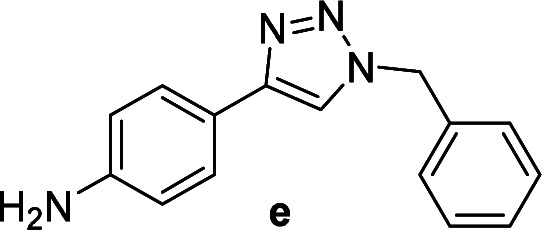	6	75	6	71	14	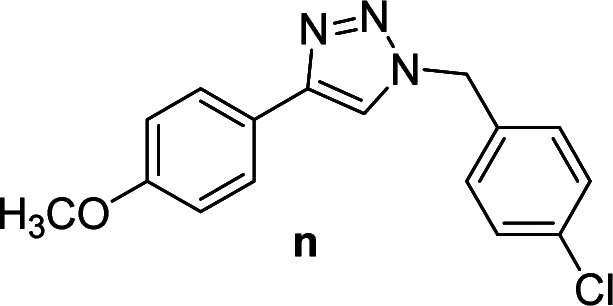	6	92	6	90
6	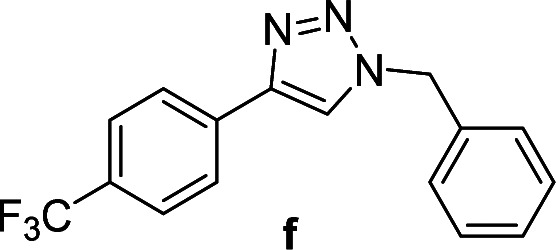	4	75	4	90	15	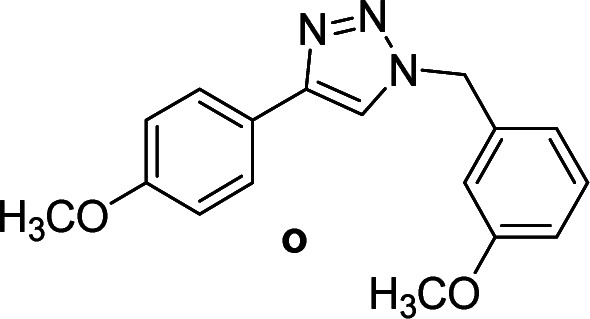	6	90	6	92
7	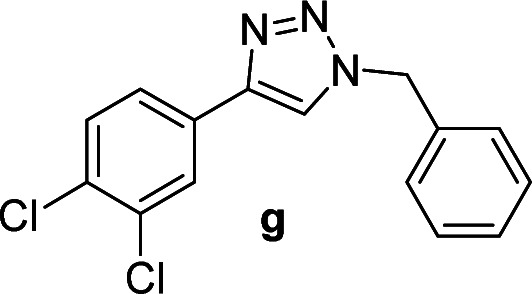	2	89	2	90	16	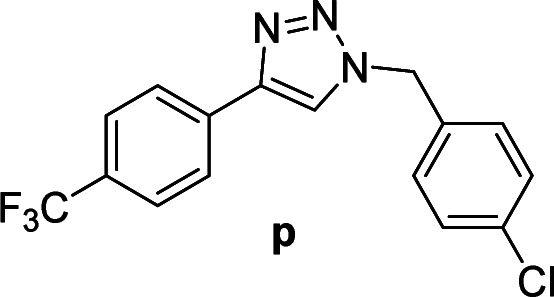	6	91	6	93
8	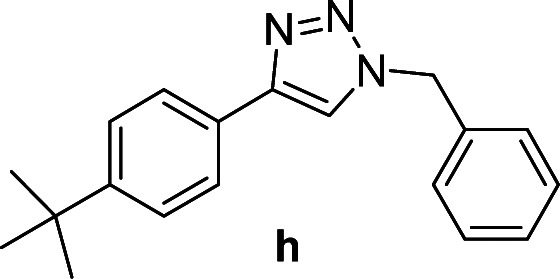	4	84	4	90	17	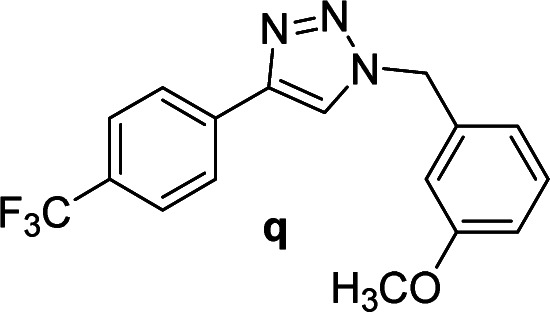	6	92	6	90
9^d^	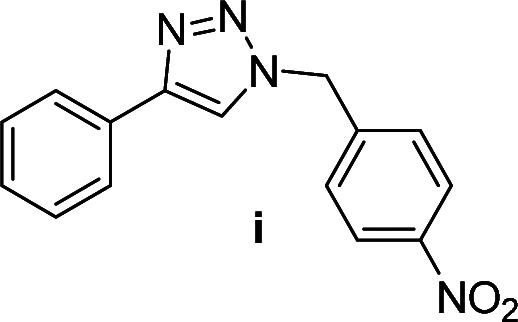	8	72	8	74						

a Reaction conditions: aryl bromide (1.0 mmol), aryl acetylene (1.0 mmol), sodium azide (1.0 mmol), Cu(i)@pNIPAM-VP (5 mol% of Cu), H_2_O (2 mL).

b Cu(ii)@pNIPAM-VP (1.0 mol% of Cu).

c 4-Aminobenzyl azide was prepared by the reaction of 4-aminobenzyl bromide with sodium azide for 2 hours; phenyl acetylene was subsequently added to the reaction mixture.

d Benzylbromide (1.2 mmol).

### 2.4. Recycling test

**Fig. 6:**
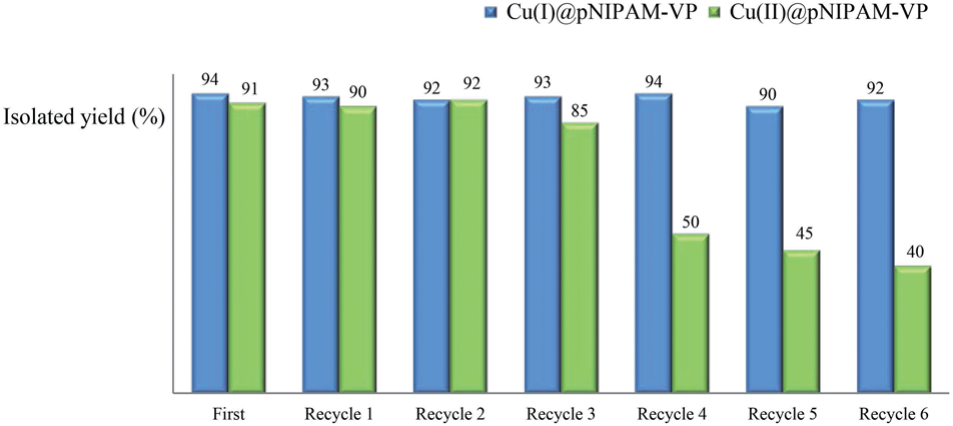
Recycling test of Cu(i)@pNIPAM-VP and Cu(ii)@pNIPAM-VP.

Recycling tests were performed using recovered catalysts under the established reaction conditions of 60 °C in water (Table 3, entries 1 & 4). As can be seen in Fig. 6, the Cu(i)@pNIPAM-VP catalyst was reusable several times, exhibiting little to no change to the product yield (90–94%). After the reaction, catalysts were collected by hot filtration and the filtrate was checked *via* ICP to confirm the leaching of copper. Fortunately, ICP data revealed no significant copper losses from the Cu(i)@pNIPAM-VP catalyst. On the other hand, in case of Cu(ii)@pNIPAM-VP, the yield decreased remarkably after being recycled three times. XPS analysis was also performed to confirm the oxidation state of freshly prepared catalyst and recovered catalyst (ESI).

## 3. Experimental

### 3.1. Material and characterization

All chemicals were purchased from commercial sources (Sigma Aldrich, TCI and Alfa Aesar) and were used without further purification unless specifically mentioned. 2-Pyridinecarboxaldehyde and 4-vinylpyridine (4-VP) were distilled under reduced pressure prior to use. The silica gel support used to prepare the catalysts was commercially available 9% functionalized 3-aminopropyl silica gel with 1 mmol NH_2_ per gram (particle size 40–63 μm; pore size 60 Å; surface area 550 m^2^ g^−1^). The copper catalyst loading on the silica support and the azide–alkyne cycloaddition reaction were performed using a shaker (Eyela, Mixer CM-1000) at 12 × 10^3^ rpm. Scanning electron microscopy (SEM) and energy dispersive X-ray analysis (EDXA) were performed on a HRTEM JEOL electron microscope at an acceleration voltage of 300 kV. The copper catalyst loading values were estimated *via* inductively coupled plasma (ICP) analysis with a JY Ultima2C. ICP analysis was also used to check for Cu leaching after the recycle test. X-ray photoelectron spectroscopy (XPS) analysis was performed on a VG SCIENTA R3000 to show the electronic state of copper. ^1^H and ^13^C NMR spectra were obtained in CDCl_3_ with a Bruker NMR at 400 MHz for ^1^H and at 100 MHz for ^13^C with TMS as an internal standard. For solid-state NMR experiments, 1D ^13^C spectra were measured *via* a cross polarization (CP) pulse sequence (contact time: 2 ms) on a Bruker 400 MHz NMR spectrometer equipped with a 4 mm magic angle spinning (MAS) probe (Bruker Biospin, Billerica, MA) operating at a 10 kHz spinning rate. Low temperature nitrogen adsorption–desorption isotherms were measured at −196 °C on an absorption volumetric analyzer BEL MINI manufactured by BEL, Inc. (Japan). Specific surface areas were determined using the Brunnauer–Emmet–Teller (BET) method from nitrogen adsorption isotherms of gas adsorbed at the relative pressure *P*/*P*_0_ = 0.99. Infrared spectra were recorded on a Bruker Alpha FT-IR spectrometer. Melting points were determined with a Sanyo Gallenkamp melting point apparatus. Analytical thin layer chromatography (TLC) was performed with E. Merck 60 F254 aluminum-backed silica gel plates (0.2 mm) containing a fluorescent indicator.

### 3.2. Synthesis of Cu(i)@IPSi and Cu(ii)@IPSi

3-Aminopropyl functionalized silica gel (3 mmol NH_2_ unit, 1.0 equiv., 3 g) was added to a jacketed vial containing a solution of 2-pyridinecarboxaldehyde (1.1 equiv., 0.34 mL) in 15 mL of distilled CH_2_Cl_2_. After 3 hours of vigorous stirring, the reaction mixture was filtered, washed with CH_2_Cl_2_, and dried under vacuum at 40 °C. To coordinate Cu(i) with silica, imine functionalized silica gel (IPSi, 1.0 equiv., 1.1 g) and [Cu(CH_3_CN)_4_]PF_6_ (1.1 equiv., 0.21 g) were added to 25 mL of methanol. The reaction mixture was shaken at room temperature for 12 hours, followed by filtration, washing with methanol, and drying under vacuum at 40 °C. The loading value of Cu(i)@IPSi was 0.498 mmol g^−1^. For the preparation of Cu(ii)@IPSi, imine functionalized silica gel (1.0 equiv., 1.1 g) was added to a jacketed vial containing CuSO_4_·5H_2_O (1.1 equiv., 0.27 g) dissolved in 25 mL of H_2_O. The mixture was stirred for 12 hours at room temperature, followed by filtration, washing with H_2_O, and drying under vacuum at 40 °C. The loading value of Cu(ii)@IPSi was 0.319 mmol g^−1^.

### 3.3. Synthesis of Cu(i)@pNIPAM-VP and Cu(ii)@pNIPAM-VP

Poly(*N*-isopropylacrylamide-*co*-4-vinylpyridine) (pNIPAM-VP) hydrogels were prepared *via* free radical solution polymerization using *N*-isopropylacrylamide (NIPAM), *N*,*N*′-methylenebis-acrylamide (MBAAm), and 4-vinylpyridine (4-VP) according to a previously reported procedure.^55^ NIPAM (10.1 mmol, 1.173 g) dissolved in 2 mL of methanol, MBAAm (1.28 mmol, 0.2 g) dissolved in 2 mL of methanol, and 4-VP (10.1 mmol, 1.06 g) dissolved in 16 mL of toluene were placed in a 100 mL flask. The polymerization was initiated by adding 2 mol% AIBN and heating the mixture at 80 °C for 4 h. A white solid polymer was filtered off, washed with methanol and diethyl ether, and dried under vacuum at 40 °C for 24 h. To coordinate Cu(i) with the polymer, pNIPAM-VP (1.0 g) and [Cu(CH_3_CN)_4_]PF_6_ (0.192 g) were added to 20 mL of methanol. The reaction mixture was shaken at room temperature for 12 hours, followed by filtration, washing with methanol, and drying under vacuum at 40 °C. The loading value of Cu(i)@pNIPAM-VP was 0.374 mmol g^−1^. For the preparation of Cu(ii)@pNIPAM-VP, polymer (1.0 g) was added to a jacketed vial containing CuSO_4_·5H_2_O (0.13 g) dissolved in 20 mL of H_2_O. The mixture was stirred for 12 hours at room temperature, followed by filtration, washing with H_2_O, and drying under vacuum at 40 °C. The loading value of Cu(ii)@pNIPAM-VP was 0.166 mmol g^−1^.

### 3.4. General procedure for triazole synthesis

For a typical triazole synthesis experiment, benzylbromide (1.0 mmol), sodium azide (1.0 mmol), phenylacetylene (1.0 mmol), and catalysts were added to a 20 mL of jacketed vial containing 2 mL of H_2_O. After the addition of the Cu(i)@IPSi catalyst (2.5 mol%), Cu(ii)@IPSi catalyst (5.0 mol%), Cu(i)@pNIPAM-VP catalyst (5.0 mol%), or Cu(ii)@pNIPAM-VP catalyst (1.0 mol%), the reaction mixture was stirred at 60 °C. The reaction was monitored *via* thin layer chromatography (TLC). After completion, the reaction mixture was cooled and 6 mL of CH_2_Cl_2_ was added, followed by filtration, and washing with CH_2_Cl_2_ and H_2_O. The reaction mixture was filtered through a pad of celite and dried *in vacuo*. The product was purified by silica gel column chromatography with hexane/ethyl acetate and analyzed by ^1^H and ^13^C NMR spectroscopy.

Compounds a,^61^b,^62^c,^63^d,^64^e,^65^f,^66^h,^67^i,^67^j,^66^k,^42^l,^68^m,^69^n,^50^ and o ^70^ are known compounds.

#### 1-Benzyl-4-(3,4-dichlorophenyl)-1*H*-1,2,3-triazole (g)

White solid, mp 135.5–136.8 °C; ^1^H NMR (400 MHz, CDCl_3_): *δ* 7.86 (d, *J* = 2.0 Hz, 1H), 7.69 (s, 1H), 7.60 (d, *J* = 8.4 Hz, 1H), 7.42 (d, *J* = 8.4 Hz, 1H), 7.38–7.36 (m, 3H), 7.31–7.28 (m, 2H), 5.58 (s, 2H); ^13^C NMR (100 MHz, CDCl_3_): *δ* 145.88, 134.23, 132.84, 131.77, 130.67, 130.46, 129.13, 128.84, 128.03, 127.29, 124.72, 119.93, 54.25; IR (KBr): 3018, 2929, 1455, 1214, 1131, 806, 667 cm^−1^; HRMS (EI): *m*/*z* [M]^+^ for C_15_H_11_Cl_2_N_3_, calculated: 303.0330, found: 303.0327.

#### 1-(4-Chlorobenzyl)-4-(4-(trifluoromethyl)phenyl)-1*H*-1,2,3-triazole (p)

White solid, mp 159.4–160.7 °C; ^1^H NMR (400 MHz, CDCl_3_): *δ* 7.91 (d, *J* = 8.1 Hz, 2H) 7.74 (s, 1H), 7.66 (d, *J* = 8.2 Hz, 2H), 7.38 (d, *J* = 8.4 Hz, 2H), 7.26 (d, *J* = 8.4 Hz, 2H), 5.56 (s, 2H); ^13^C NMR (100 MHz, CDCl_3_): *δ* 146.99, 135.03, 133.77, 132.84, 130.08 (q, *J* = 32 Hz), 129.45, 129.42, 125.82 (q, *J* = 2.88 Hz), 125.81, 124.02 (q, *J* = 270.2 Hz), 120.13, 53.60; IR (KBr): 3105, 3020, 1920, 2850, 1621, 1325, 1229, 1162, 1125, 1064, 1015, 744, 668 cm^−1^; HRMS (EI): *m*/*z* [M]^+^ for C_16_H_11_ClF_3_N_3_, calculated: 337.0594, found: 337.0591.

#### 1-(3-Methoxybenzyl)-4-(4-(trifluoromethyl)phenyl)-1*H*-1,2,3-triazole (q)

White solid, mp 116.2–117.4 °C; ^1^H NMR (400 MHz, CDCl_3_): *δ* 7.91 (d, *J* = 8.0 Hz, 2H), 7.75 (s, 1H), 7.65 (d, *J* = 8.4 Hz, 2H), 7.31 (t, *J* = 8.0 Hz, 1H), 6.91 (d, *J* = 8.3 Hz, 2H), 6.84 (t, *J* = 1.9 Hz, 1H); ^13^C NMR (100 MHz, CDCl_3_): *δ* 160.21, 146.86, 135.82, 133.98, 130.36, 129.97 (q, *J* = 32.6 Hz), 125.83, 125.81 (q, *J* = 3.0 Hz), 124.10 (q, *J* = 270.4 Hz), 120.32, 120.28, 114.30, 113.84, 55.34, 54.33; IR (KBr): 3108, 3016, 2948, 2844, 1614, 1587, 1329, 1164, 1109, 1067, 835, 752 cm^−1^; HRMS (EI): *m*/*z* [M]^+^ for C_17_H_14_F_3_N_3_O, calculated: 333.1089, found: 333.1088.

## 4. Conclusions

Two types of solid supports were synthesized for the preparation of heterogeneous catalysts. One was aminopropyl-functionalized reverse phase silica gel, which possessed an end capped hydrophobic alkyl group. The other was a thermoresponsive poly(*N*-isopropylacrylamide-*co*-4-vinylpyridine) (pNIPAM-VP), which exhibited hydrophilicity and hydrophobicity according to temperature. These catalyst properties enabled one-pot azide–alkyne cycloaddition reactions in water. A series of 1,4-disubstituted-1,2,3-triazoles were synthesized with good results and the catalysts could be reused multiple times.

## Conflicts of interest

There are no conflicts to declare.

## Supplementary Material

RA-008-C8RA00306H-s001
