# Supplementary material for: Azide–alkyne cycloaddition reactions in water via recyclable heterogeneous Cu catalysts: reverse phase silica gel and thermoresponsive hydrogels
Source: RSC Adv. 2018 Feb 6;8(11):6152–9. doi: 10.1039/c8ra00306h (PMC9078218; doi:10.1039/c8ra00306h)
Supplement: RA-008-C8RA00306H-s001 [file RA-008-C8RA00306H-s001.pdf]

Electronic Supplementary Material (ESI) for RSC Advances.
This journal is © The Royal Society of Chemistry 2018

Electronic Supplementary Information

Azide–alkyne cycloaddition reactions in water via recyclable heterogeneous catalysts: reverse phase silica gel and thermoresponsive hydrogels

Minkyung Lim,[†a] Heejin Lee,[†a] Minseok Kang,[b] Woncheol Yoo[b] and Hakjune Rhee[*a,b]

[a] Department of Bionanotechnology, Hanyang University, 55 Hanyangdaehak-ro, Sangnok-gu, Ansan, Gyeonggi-do, 15588, South Korea

[b] Department of Applied Chemistry, Hanyang University, 55 Hanyangdaehak-ro, Sangnok-gu, Ansan, Gyeonggi-do, 15588, South Korea

† These authors contributed equally.

Table of Contents

1. SEM and EDXA image …………………………………………………………….. S2
2. BET data ………………………………………………………………………………… S3
3. XPS data ………………………………………………………………………………… S4
4. Solid-state ^{13}C NMR of functionalized silica ……………………………………………… S5
5. ^{1}H NMR, ^{13}C NMR, IR and HRMS of product ……………………………………….... S6
6. References ……………………………………………………………………………..... S43

1. SEM and EDXA image

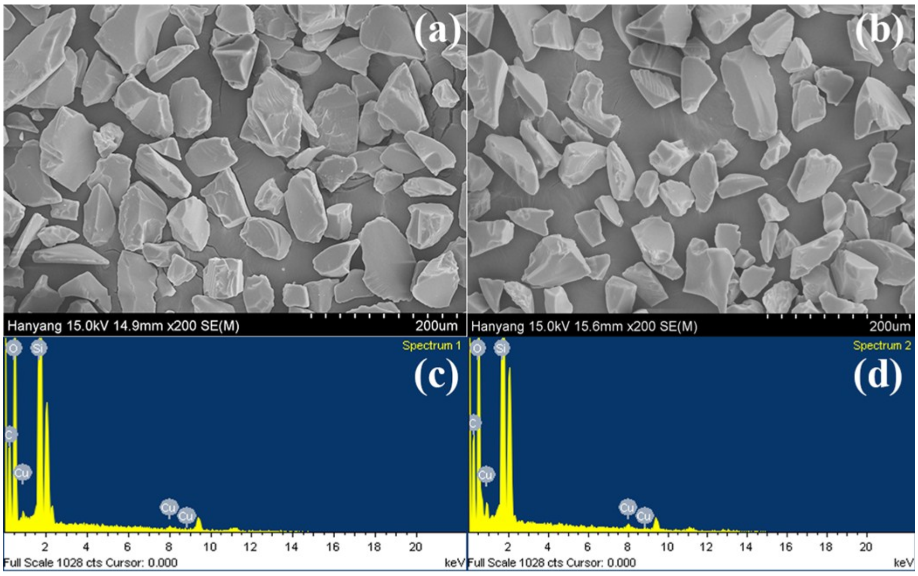

Figure S1. SEM and EDXA image of Cu(I)@IPSi(a,c) and Cu(II)@IPSi(b,d).

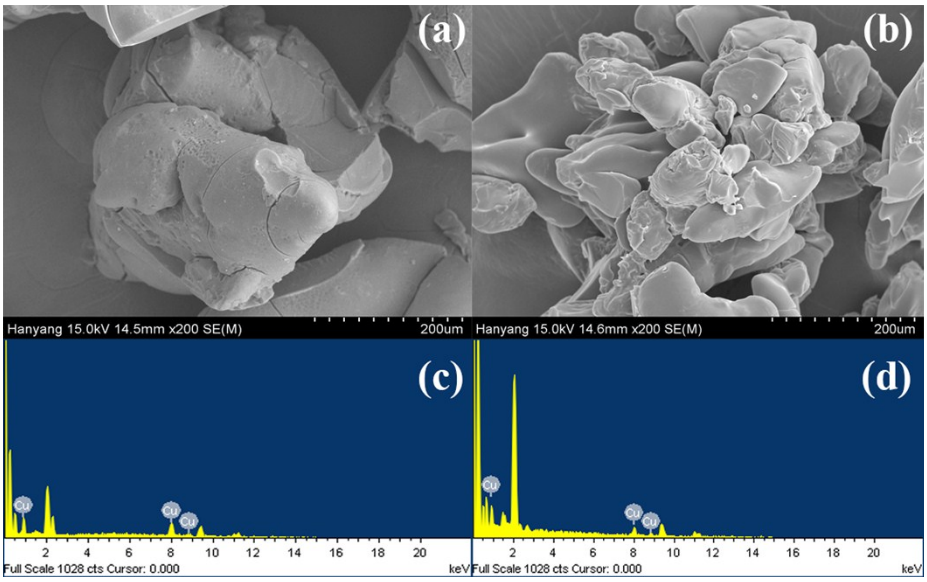

Figure S2. SEM and EDXA image of Cu(I)@pNIPAM-VP(a,c) and Cu(II)@pNIPAM-VP(b,d).

2. BET data

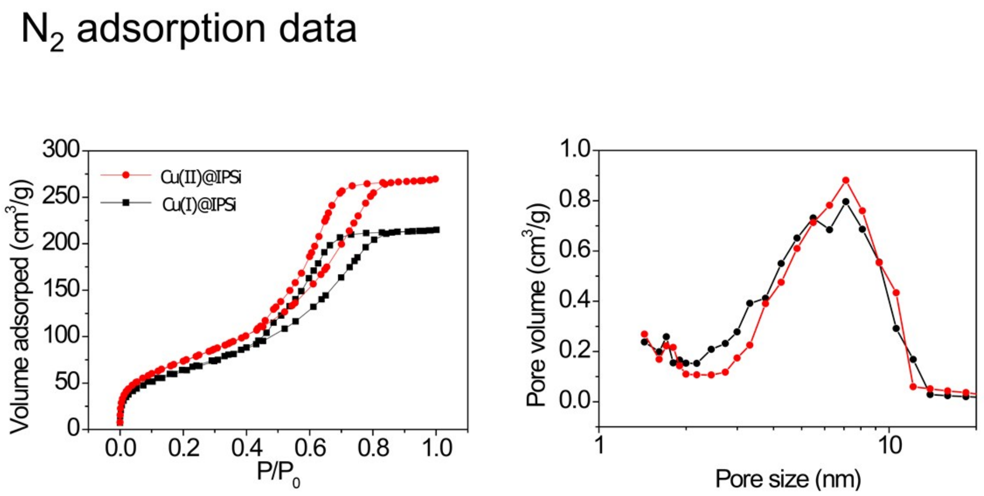

Sample	S_{BET} (m^2/g)	V_{tot} (cm^3/g)
Cu(I)@IPSi	232.00	0.332
Cu(II)@IPSi	275.17	0.416

Figure S3. BET data of Cu(I)@IPSi and Cu(II)@IPSi

3. XPS data

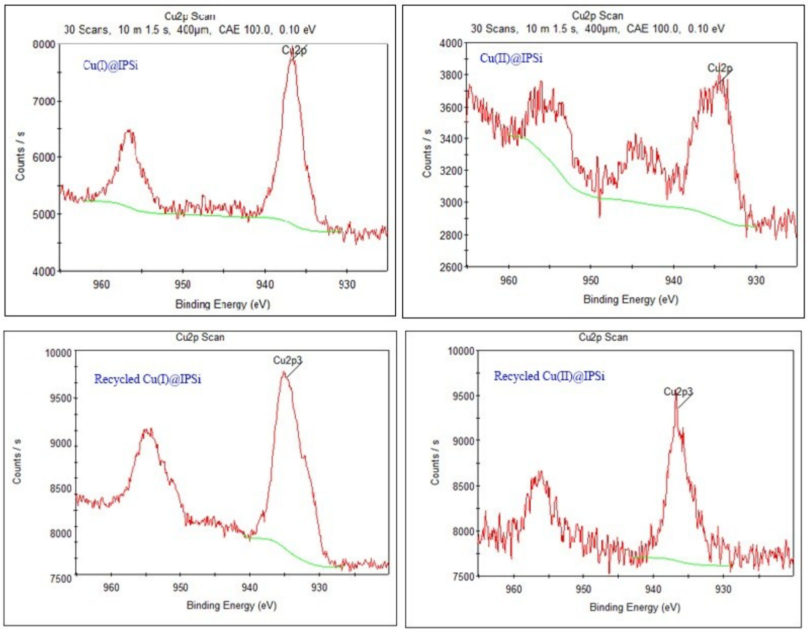

Figure S4. XPS data of fresh and recycled Cu@IPSi catalyst

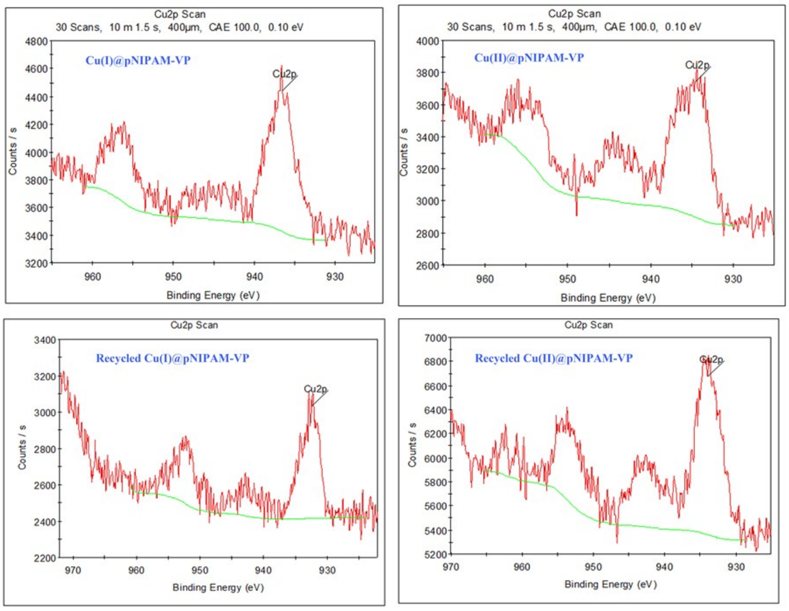

Figure S5. XPS data of fresh and recycled Cu@pNIPAM-VP catalyst

X-ray photoelectron spectroscopy (XPS) analysis was performed to confirm the oxidation state of freshly prepared catalyst and recovered catalyst. As shown in Figure S4, the oxidation number of recovered Cu(I)@IPSi after the reaction was maintained, but the oxidation number of Cu(II)@IPSi was changed. Despite the change of oxidation state, Cu(II)@IPSi catalyst was able to be reused several times. In the case of Cu@pNIPAM-VP catalysts, the loading value of Cu was relatively low and it was difficult to distinguish between the peak of Cu(I) and Cu(II). However, the peaks on the XPS data of freshly prepared catalyst and recovered catalyst appear to be unchanged (Figure S5).

4. Solid-state ^{13}C NMR of functionalized silica

For solid-state NMR experimens, 1D ^{13}C spectra were measured by cross polarization (CP) pulse sequence (contact time: 2 ms) on a Bruker 400 MHz NMR spectrometer equipped with a 4 mm magic angle spinning (MAS) probe (Bruker Biospin, Billerica, MA) operated at a 10 kHz spinning rate. A total of 8,000 transients were acquired for 11 h, and the time delay between each transient was 5 s. For each scan, 2,048 data points were acquired for an acquisition time of 34.4 ms. ^{1}H decoupling was applied for the acquisition time with SPINAL64. The raw data were zero filled to 4,096 complex data points, and an exponential window function with 50 Hz line broadening was applied before Fourier transform using the TOPSPIN 3.3 program (Bruker Biospin, Billerica, MA). All of the chemical shifts were calibrated with adamantane at 38.48 ppm.

Solid-state ^{13}C NMR (43, 25, 10).

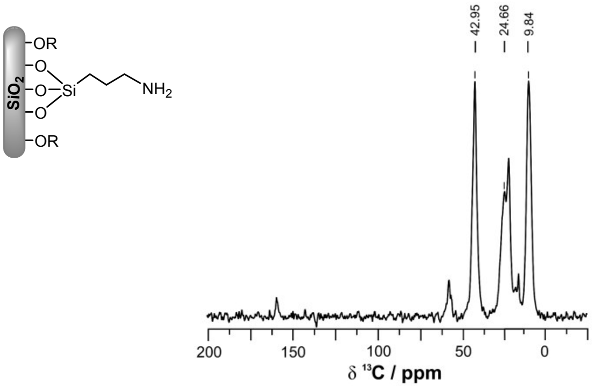

Figure S6. Solid-state ^{13}C NMR of 3-aminopropyl-functionalized silica (APSi)

Solid-state ^{13}C NMR (162, 155, 148, 136, 121, 63, 43, 24, 10).

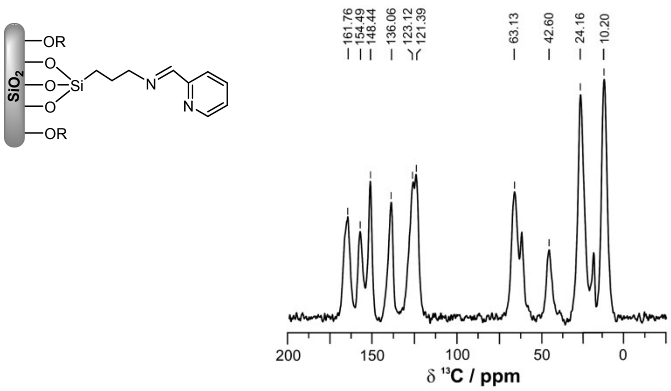

Figure S7. Solid-state ^{13}C NMR of iminopropyl-functionalized silica (IPSi)

5. ^{1}H NMR, ^{13}C NMR, IR and HRMS of product

1-Benzyl-4-phenyl-1*H*-1,2,3-triazole(a)[1]

White solid; yield: Cu(I)@IPSi 0.216 g (92%), Cu(II)@IPSi 0.219 g (93%), Cu(I)@pNIPAM-VP 0.221 g (94%), Cu(II)@pNIPAM-VP 0.214 g (91 %); R_f = 0.2 (hexane/ethyl acetate = 5:1, v/v); m.p. 126.7-127.2 ^oC; ^{1}H NMR (400 MHz, $CDCl_3$): δ 7.79-7.76 (m, 2H), 7.64 (s, 1H), 7.40-7.27 (m, 8H), 5.56 (s, 2H); ^{13}C NMR (100 MHz, $CDCl_3$): δ 148.42, 134.89, 130.74, 129.36, 129.01, 128.98, 128.36, 128.26, 125.89, 119.71, 54.42; CAS Number: 108717-96-0.

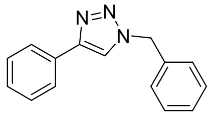

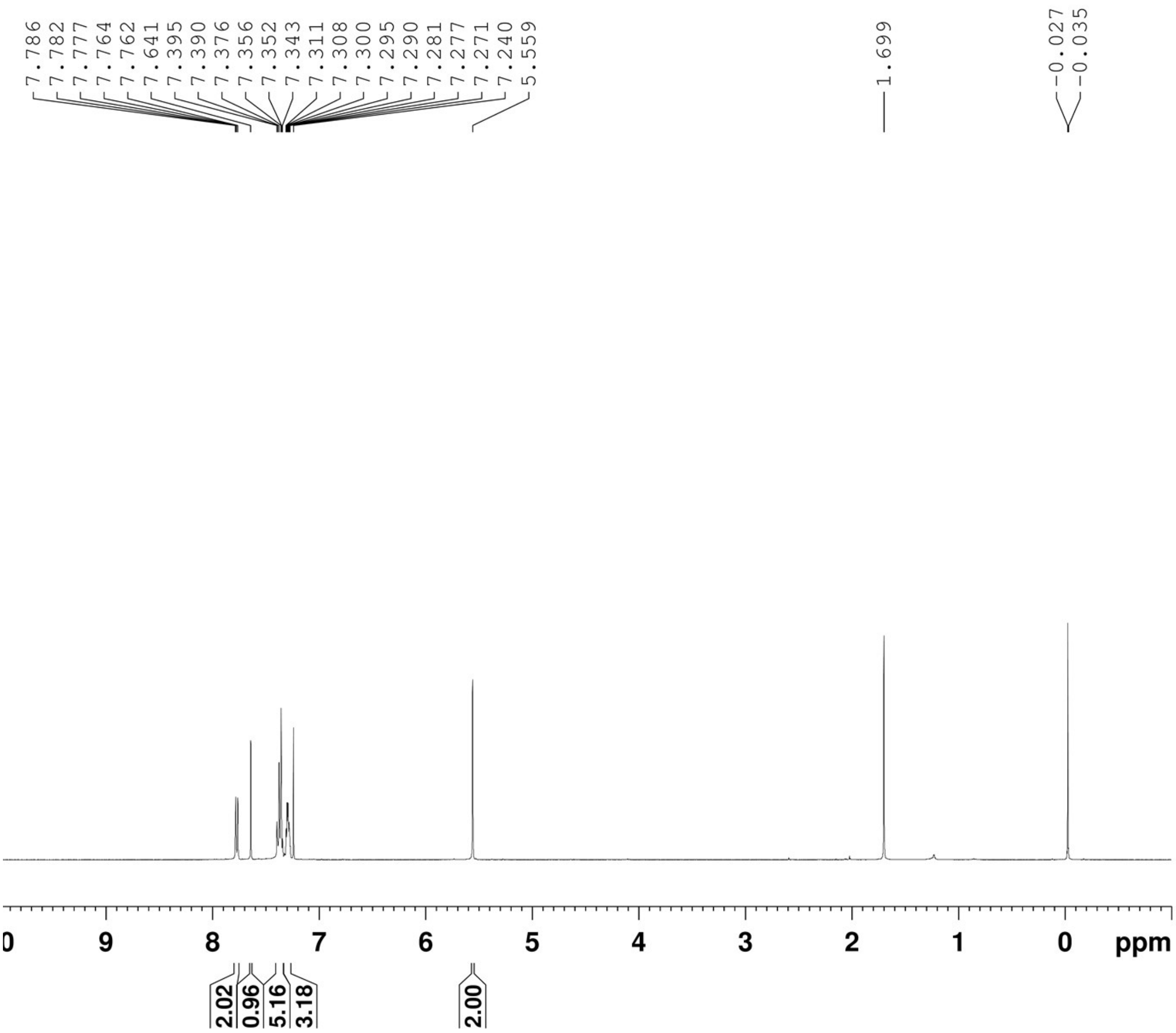

Figure S8. ^{1}H NMR (400 MHz, $CDCl_3$) spectra of compound **a**

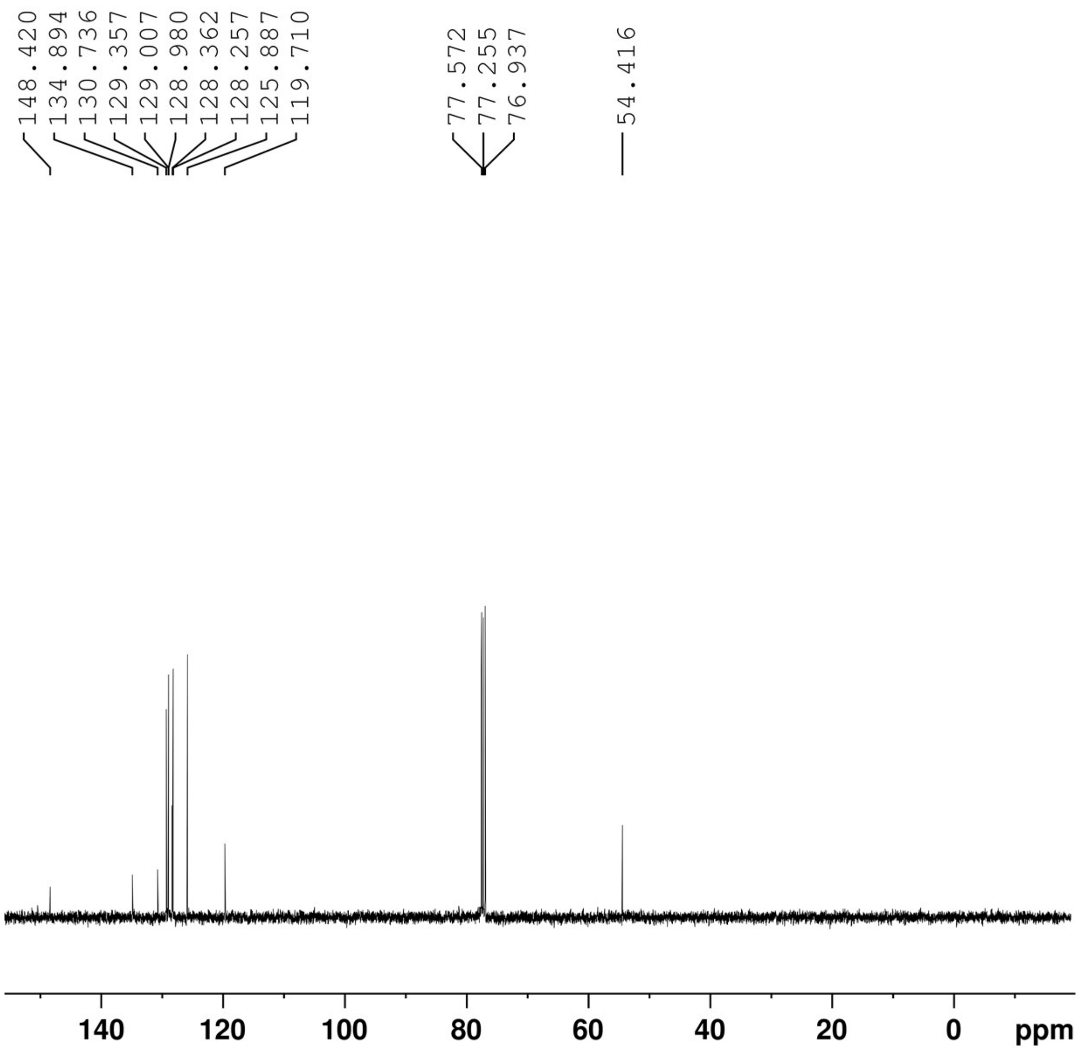

Figure S9. ^{13}C NMR (100 MHz, $CDCl_3$) spectra of compound **a**

1-Benzyl-4-(*p*-tolyl)-1*H*-1,2,3-triazole(b)[2]

White solid, yield: Cu(I)@IPSi 0.232 g (93%), Cu(II)@IPSi 0.224 g (90%), Cu(I)@pNIPAM-VP 0.229g (92 %), Cu(II)@pNIPAM-VP 0.232 g (93%); $R_f = 0.13$ (hexane/ethyl acetate = 5:1, v/v); m.p. 150.9-151.5 °C; ^1H NMR (400 MHz, $CDCl_3$): δ 7.69 (d, J = 8.1 Hz, 2H), 7.62 (s, 1H), 7.39-7.30 (m, 5H), 7.20 (d, J = 7.9 Hz, 2H), 5.57 (s, 2H), 2.36 (s, 3H); ^{13}C NMR (100 MHz, $CDCl_3$): δ 148.54, 138.22, 134.97, 129.70, 129.36, 129.98, 128.29, 127.94, 125.81, 119.35, 54.42, 21.51; CAS Number: 852657-84-2.

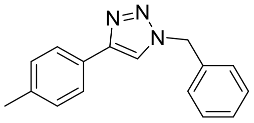

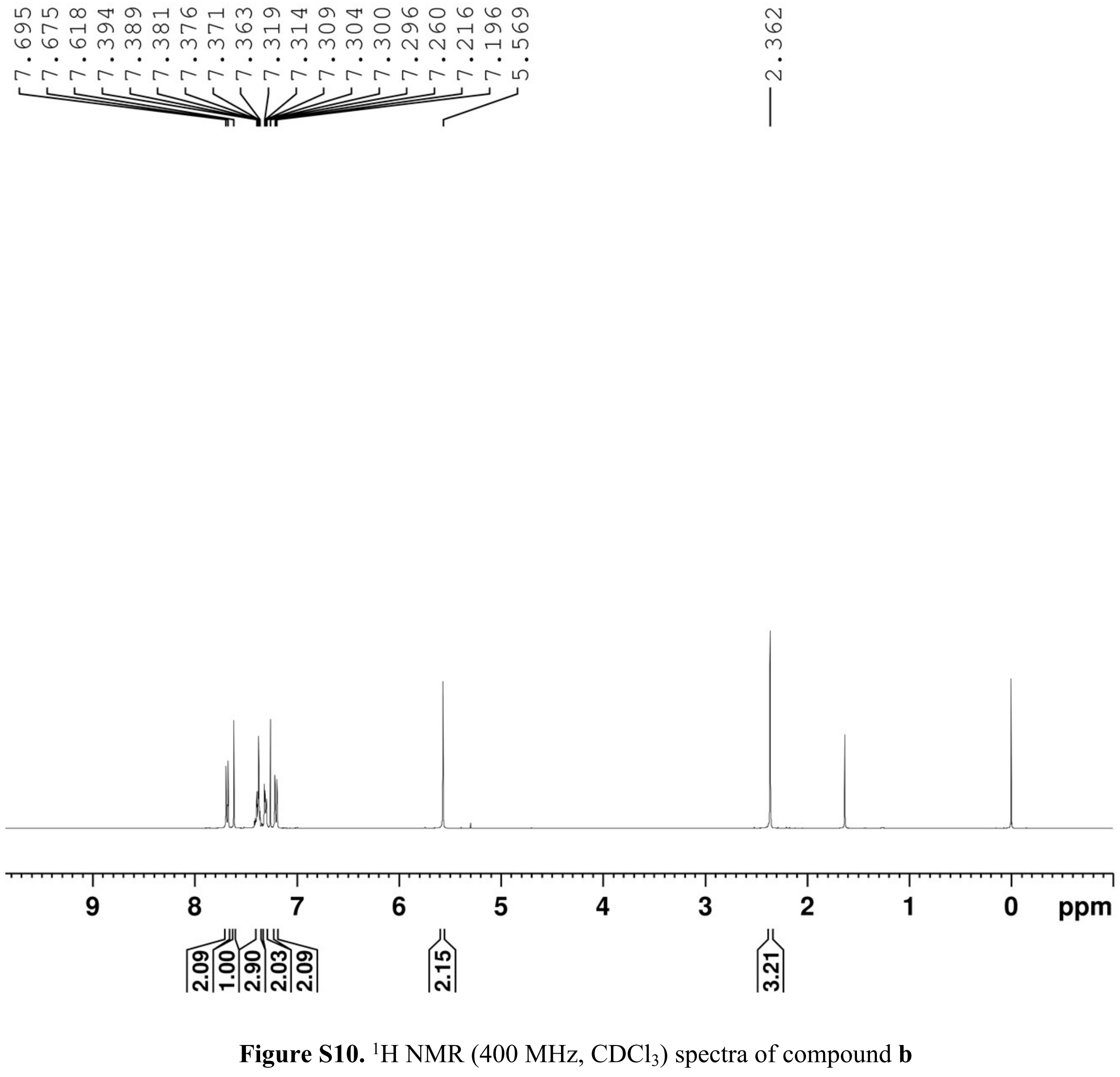

Figure S10. ^1H NMR (400 MHz, $CDCl_3$) spectra of compound **b**

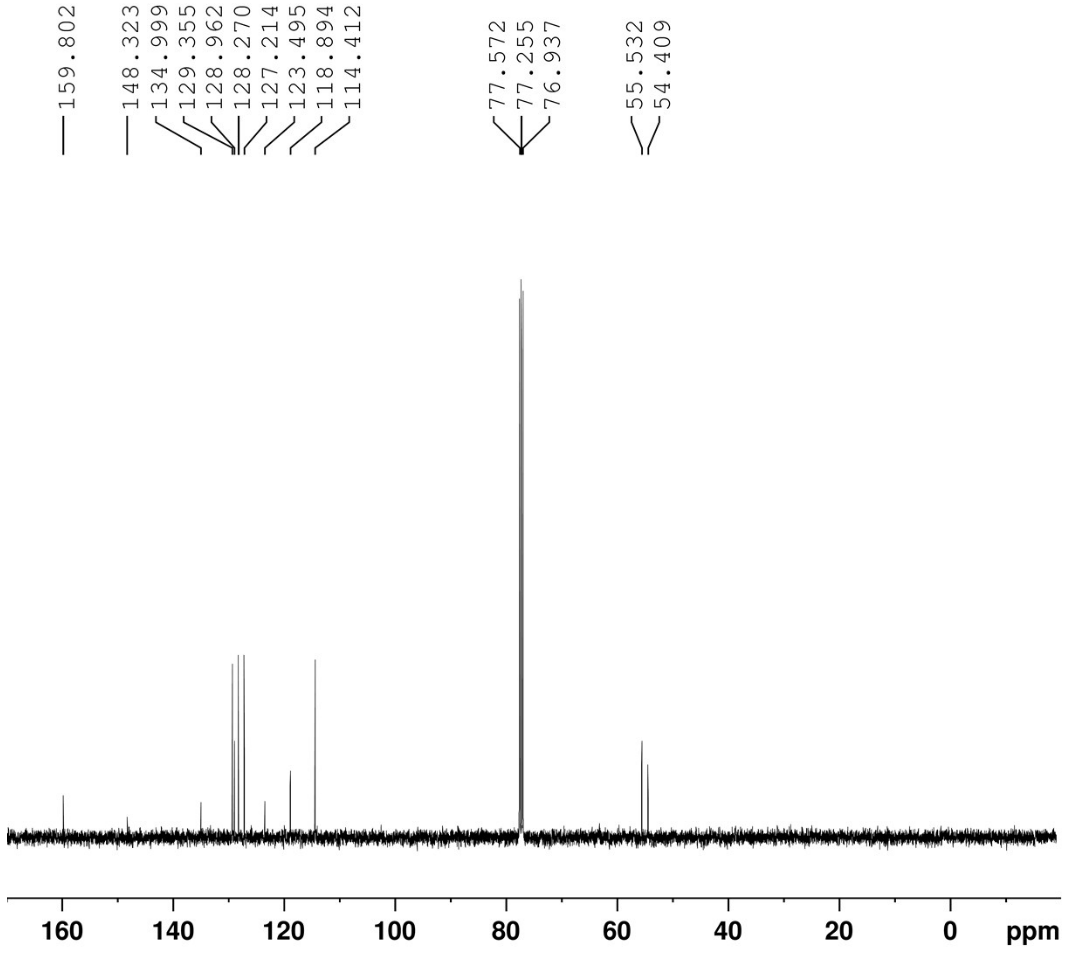

Figure S11. ^{13}C NMR (100 MHz, $CDCl_3$) spectra of compound **b**

1-Benzyl-4-(4-methoxyphenyl)-1*H*-1,2,3-triazole(c)[3]

White solid; yield: Cu(I)@IPSi 0.265 g (Quant.), Cu(II)@IPSi 0.233 g (88%), Cu(I)@pNIPAM-VP 0.244 g (92%), Cu(II)@pNIPAM-VP 0.244 g (92%); R_f = 0.13 (hexane/ethyl acetate = 5:1, v/v); m.p. 146.0-146.7 °C; ^{1}H NMR (400 MHz, $CDCl_3$): δ 7.72 (d, *J* = 8.8 Hz, 2H), 7.57 (s, 1H), 7.39-7.29 (m, 5H), 6.93 (d, *J* = 8.9 Hz, 2H), 5.56 (s, 2H), 3.83 (s, 3H); ^{13}C NMR (100 MHz, $CDCl_3$): δ 159.80, 148.32, 135.00, 129.36, 128.96, 128.27, 127.21, 123.50, 118.89, 114.41, 54.53, 54.41; CAS Number: 116557-81-4.

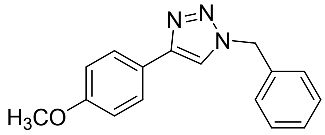

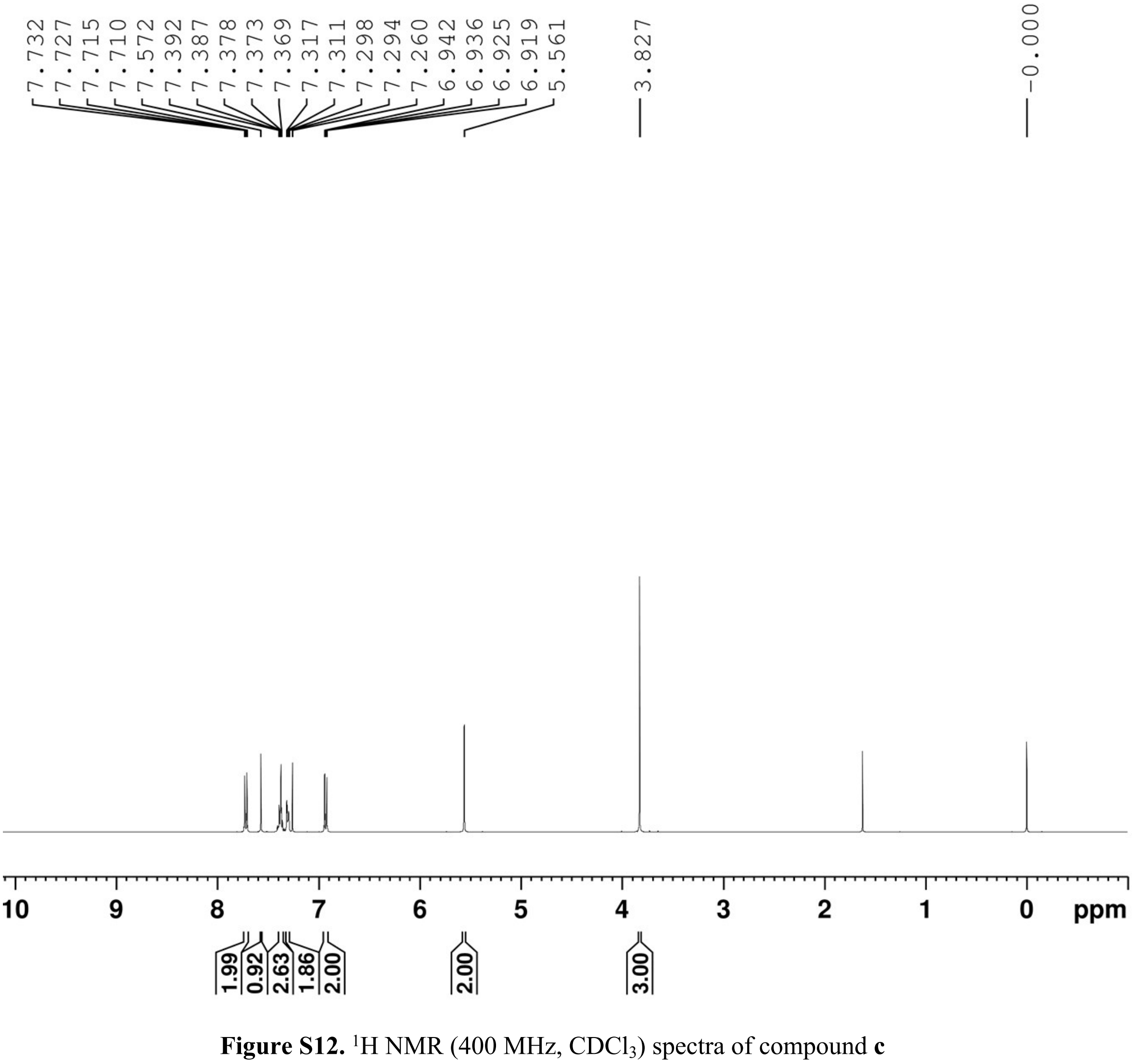

Figure S12. ^{1}H NMR (400 MHz, $CDCl_3$) spectra of compound **c**

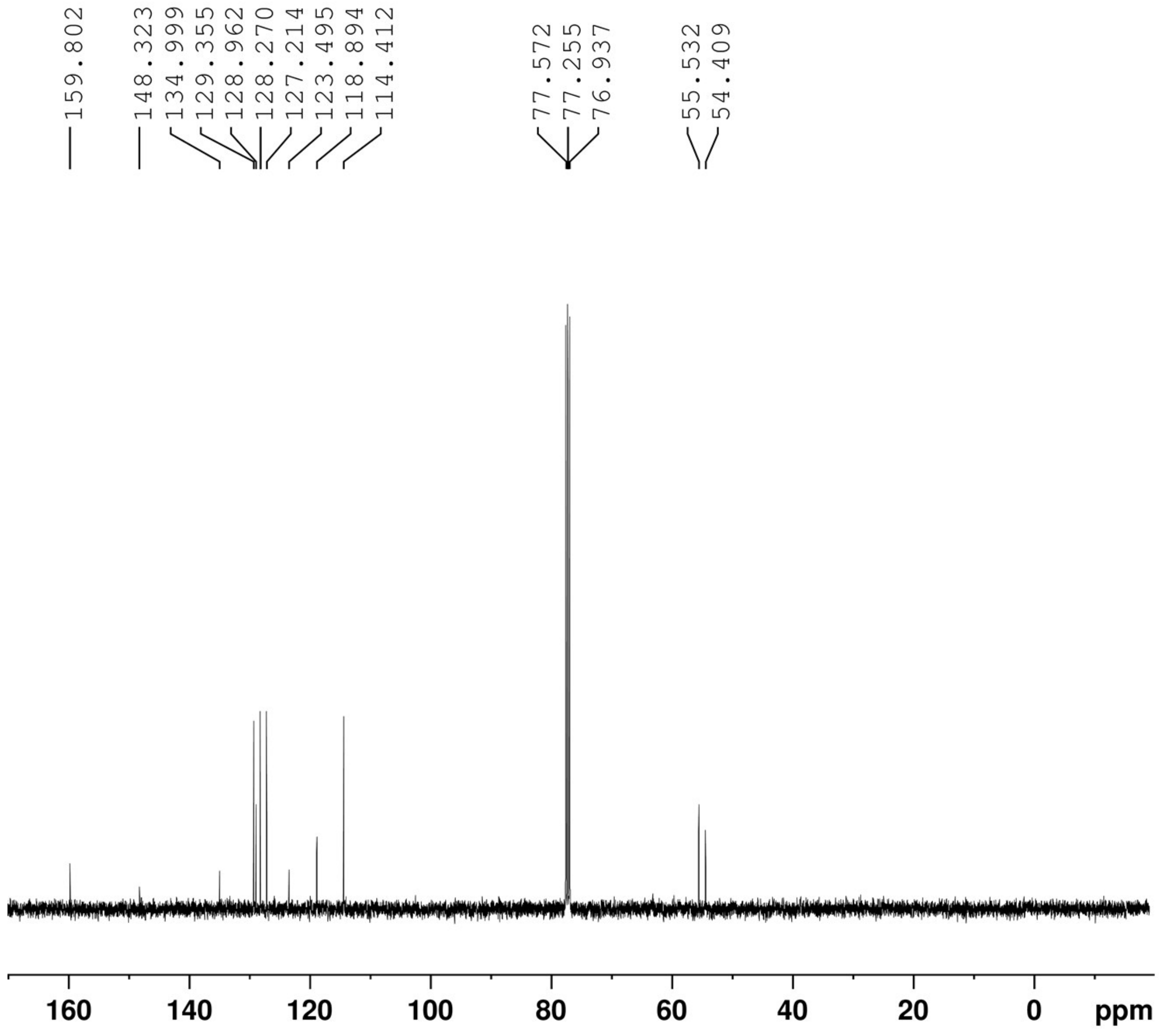

Figure S13. ^{13}C NMR (100 MHz, $CDCl_3$) spectra of compound **c**

1-Benzyl-4-(4-bromophenyl)-1*H*-1,2,3-triazol(d)[4]

White solid, yield: Cu(I)@IPSi 0.305 g (97%), Cu(II)@IPSi 0.286 g (91%), Cu(I)@pNIPAM-VP 0.292 g (93%), Cu(II)@pNIPAM-VP 0.289 g (92%); R_f = 0.25 (hexane/ethyl acetate = 5:1, v/v); m.p. 150.0-151.1 ^oC; ^1H NMR (400 MHz, $CDCl_3$): δ 7.67 (s, 1H), 7.65 (d, *J* = 8.6 Hz, 2H), 7.49 (d, *J* = 8.5 Hz, 2H), 7.38-7.28 (m, 5H), 5.55 (s, 2H); ^{13}C NMR (100 MHz, $CDCl_3$): δ 147.42, 134.71, 132.17, 129.73, 129.44, 129.12, 128.34, 127.44, 122.26, 119.77, 54.54; CAS Number: 1009089-48-8.

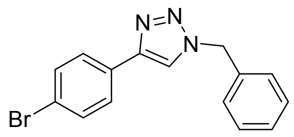

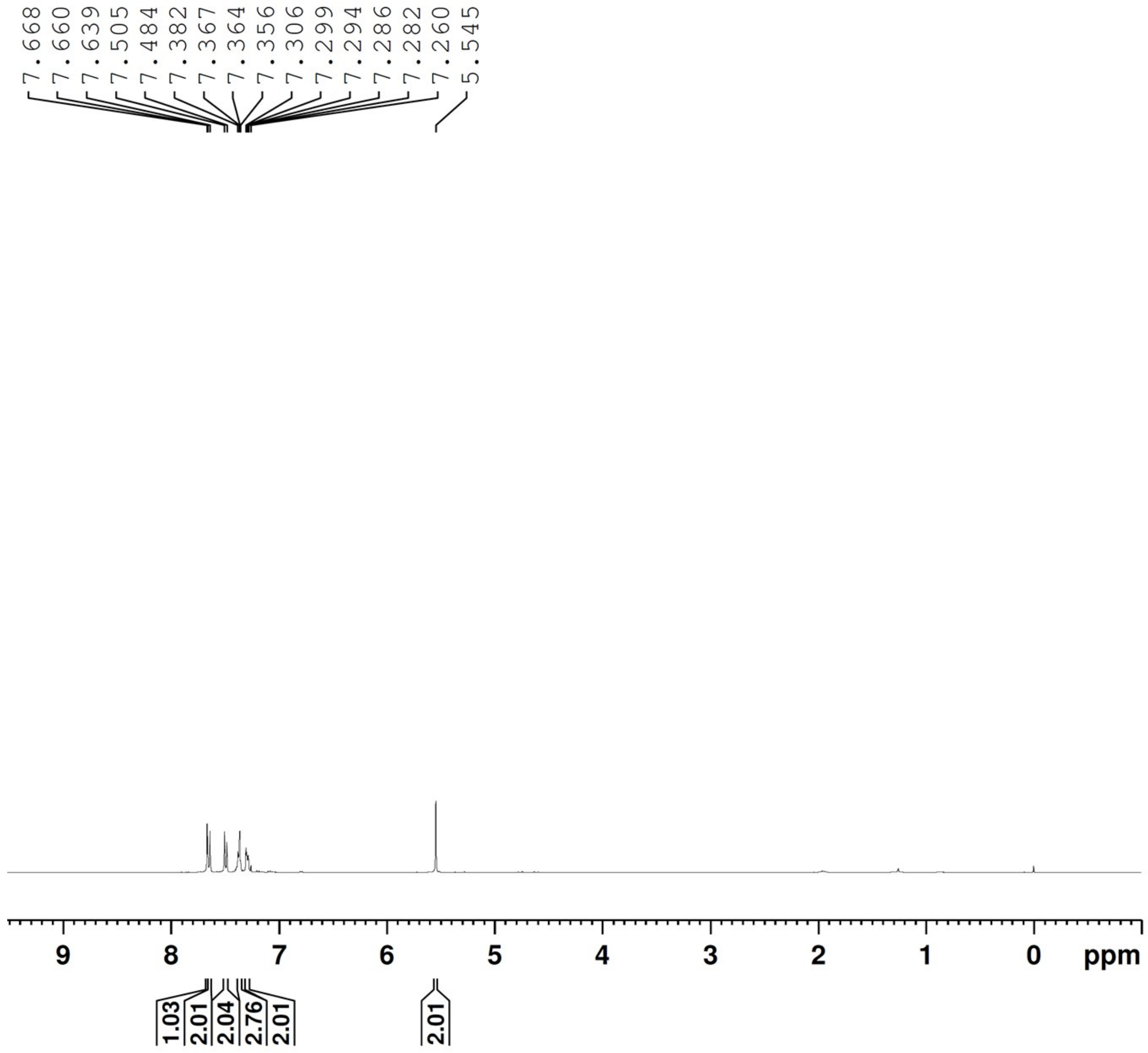

Figure S14. ^1H NMR (400 MHz, $CDCl_3$) spectra of compound **d**

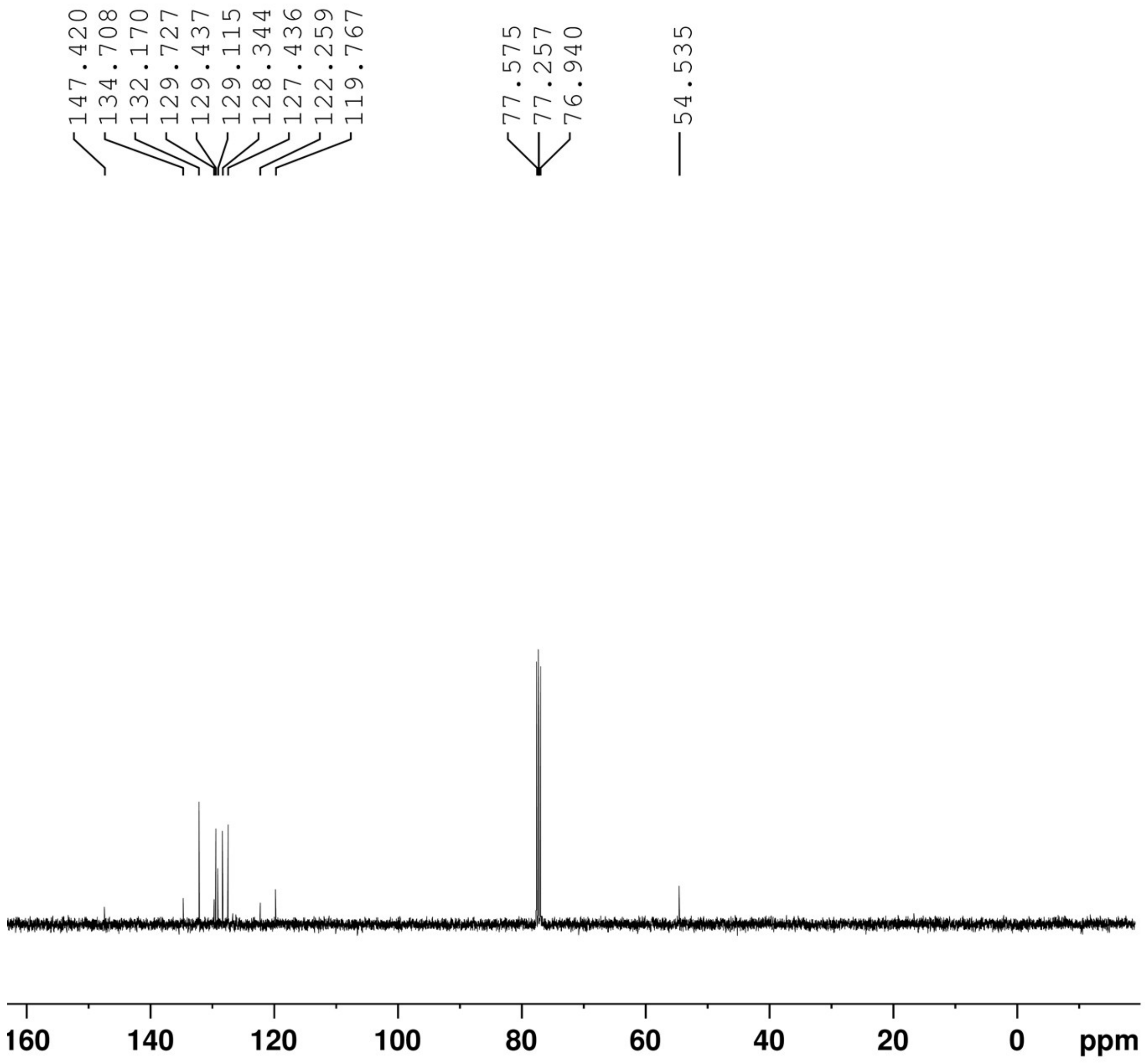

Figure S15. ^{13}C NMR (100 MHz, $CDCl_3$) spectra of compound **d**

4-(1-Benzyl-1*H*-1,2,3-triazol-4-yl)aniline(e)[4]

White solid, yield: Cu(I)@IPSi 0.190g (76%), Cu(II)@IPSi 0.185 g (74%), Cu(I)@pNIPAM-VP 0.188 g (75%), Cu(II)@ pNIPAM-VP 0.178 g (71%); R_f = 0.05 (hexane/ethyl acetate = 5:1, v/v); m.p. 183.1-184.5 ^{o}C; ^{1}H NMR (400 MHz, $CDCl_3$): δ 7.58 (d, *J* = 8.6 Hz, 2H), 7.52 (s, 1H), 7.38-7.27 (m, 5H), 6.70 (d, *J* = 8.6 Hz, 2H), 5.53 (s, 2H), 3.85 (s, 2H); ^{13}C NMR (100 MHz, $CDCl_3$): δ 148.58, 146.53, 134.88, 129.12, 128.70, 128.04, 126.92, 121.03, 118.26, 115.23. 54.15; CAS Number: 104951-46-4.

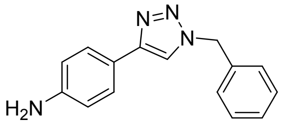

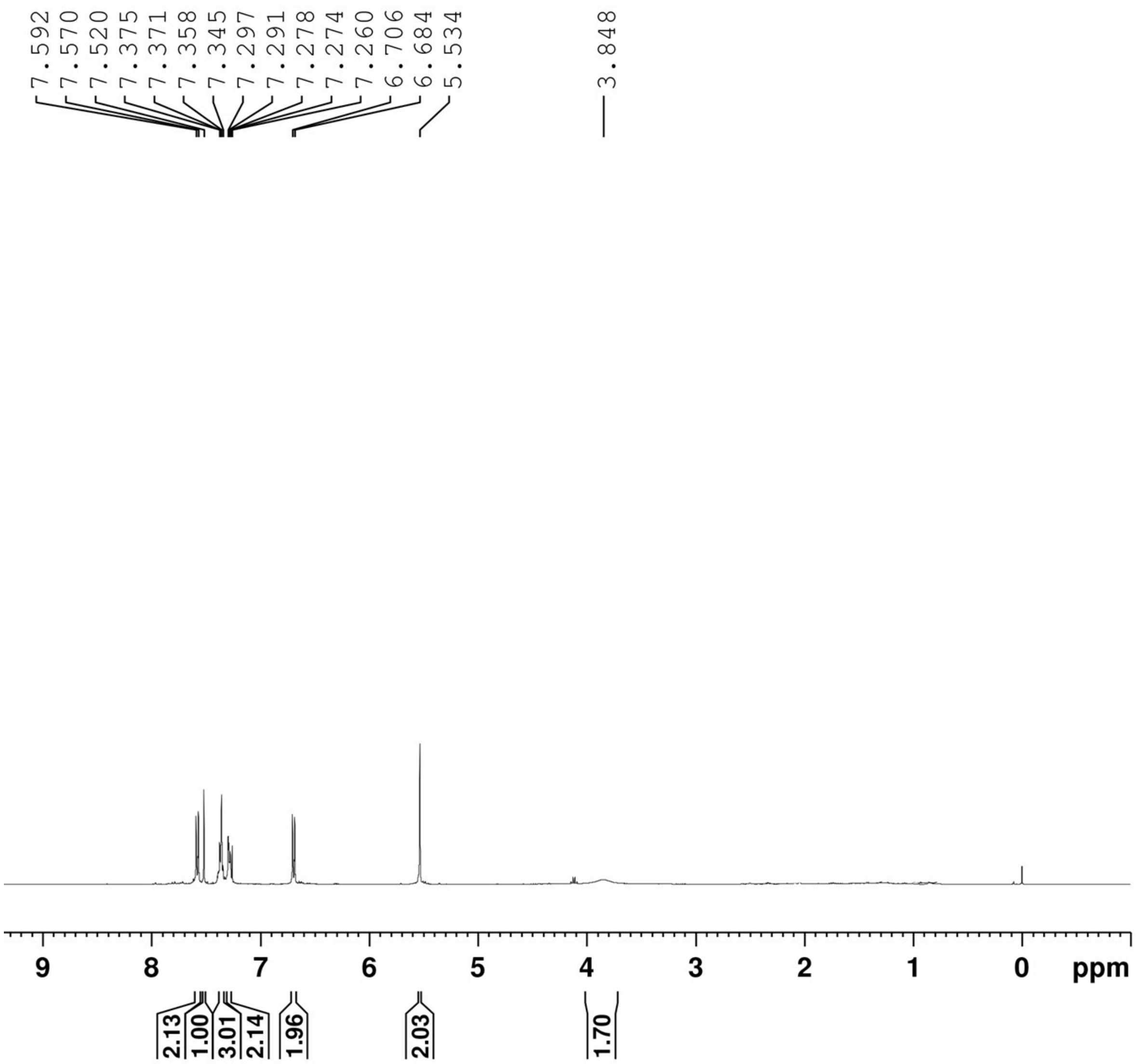

Figure S16. ^{1}H NMR (400 MHz, $CDCl_3$) spectra of compound **e**

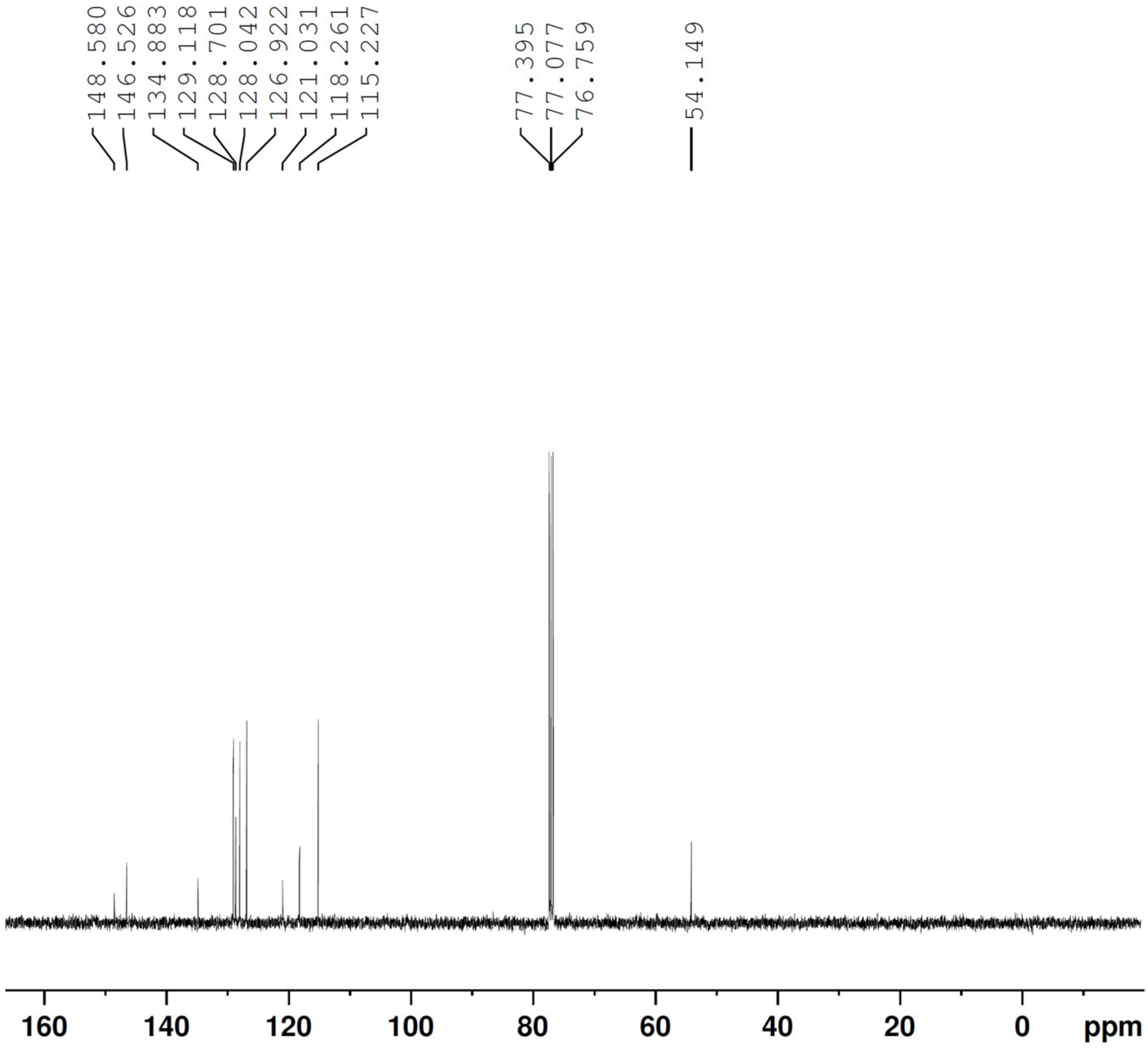

Figure S17. ^{13}C NMR (100 MHz, $CDCl_3$) spectra of compound **e**

1-Benzyl-4-(4-(trifluoromethyl)phenyl)-1*H*-1,2,3-triazole(f)[5]

White solid, yield: Cu(I)@IPSi 0.273 g (90%), Cu(II)@IPSi 0.273 g (90%), Cu(I)@pNIPAM-VP 0.227 g (75%), Cu(II)@pNIPAM-VP 0.273 g (90%); R_f = 0.35 (hexane/ethyl acetate = 5:1, v/v); m.p. 138.1-139.5 °C; ^{1}H NMR (400 MHz, $CDCl_3$): δ 7.90 (d, J = 8.0 Hz, 2H), 7.77 (s, 1H), 7.63 (d, J = 8.0 Hz, 2H), 7.41-7.30 (m, 5H), 5.57 (s, 2H). ^{13}C NMR (100 MHz, $CDCl_3$): δ 146.85, 134.40, 133.97, 128.98 (q, J = 32.6 Hz), 129.26, 128.97, 128.15, 125.76, 125.74 (q, J = 4.1 Hz), 124.10 (q, J = 240.4 Hz), 120.31, 54.38; CAS Number: 1237683-33-8.

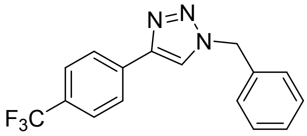

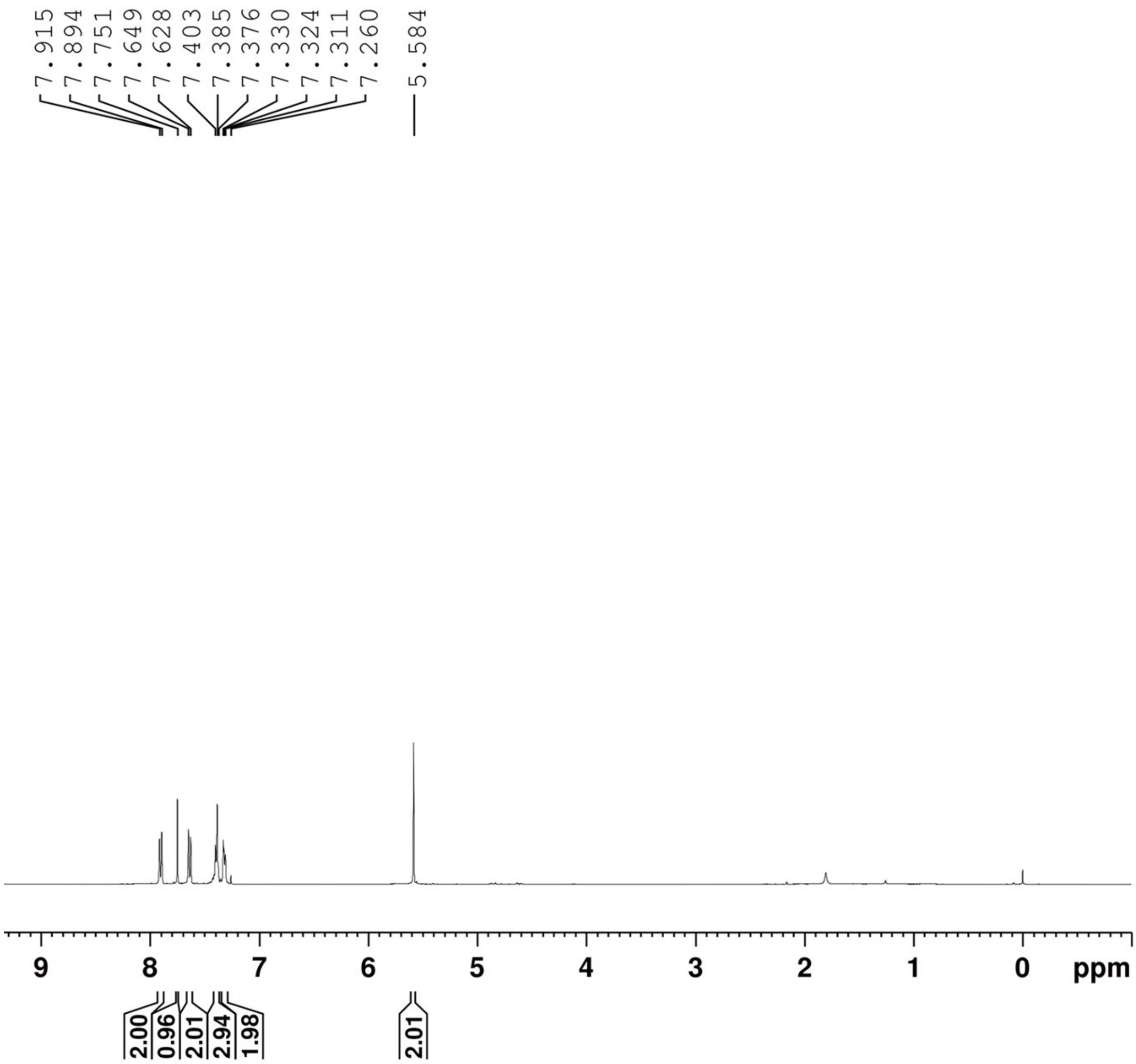

Figure S18. ^{1}H NMR (400 MHz, $CDCl_3$) spectra of compound **f**

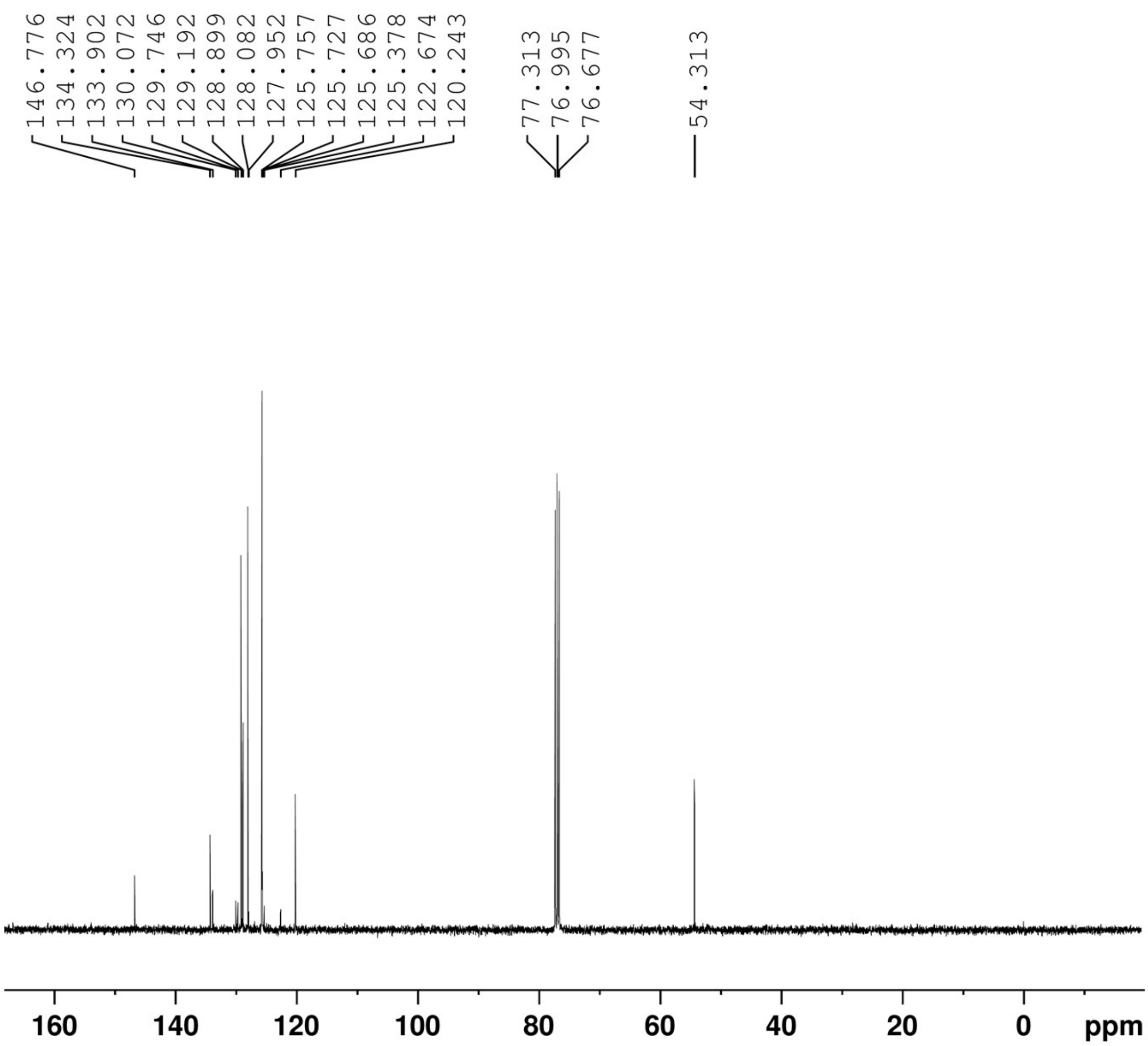

Figure S19. ^{13}C NMR (100 MHz, $CDCl_3$) spectra of compound **f**

1-Benzyl-4-(3,4-dichlorophenyl)-1*H*-1,2,3-triazole(g)

White solid, yield: Cu(I)@IPSi 0.292 g (96%), Cu(II)@IPSi 0.268 g (88%), Cu(I)@pNIPAM-VP 0.271 g (89%), Cu(II)@pNIPAM-VP 0.274 g (90%); R_f = 0.13 (hexane/ethyl acetate = 5:1, v/v); m.p. 135.5-136.8 ^{o}C; ^{1}H NMR (400 MHz, $CDCl_3$): δ 7.86 (d, *J* = 2.0 Hz, 1H), 7.69 (s, 1H), 7.60 (d, *J* = 8.4 Hz, 1H), 7.42 (d, *J* = 8.4 Hz, 1H), 7.38-7.36 (m, 3H), 7.31-7.28 (m, 2H), 5.58 (s, 2H); ^{13}C NMR (100 MHz, $CDCl_3$): δ 145.88, 134.23, 132.84, 131.77, 130.67, 130.46, 129.13, 128.84, 128.03, 127.29, 124.72, 119.93, 54.25; IR(KBr): 3018, 2929, 1455, 1214, 1131, 806, 667 cm^{-1}; HRMS(EI): m/z $[M]^+$ for $C_{15}H_{11}Cl_2N_3$, calculated: 303.0330, found: 303.0327.

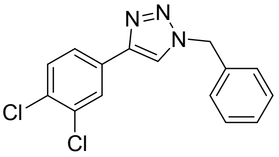

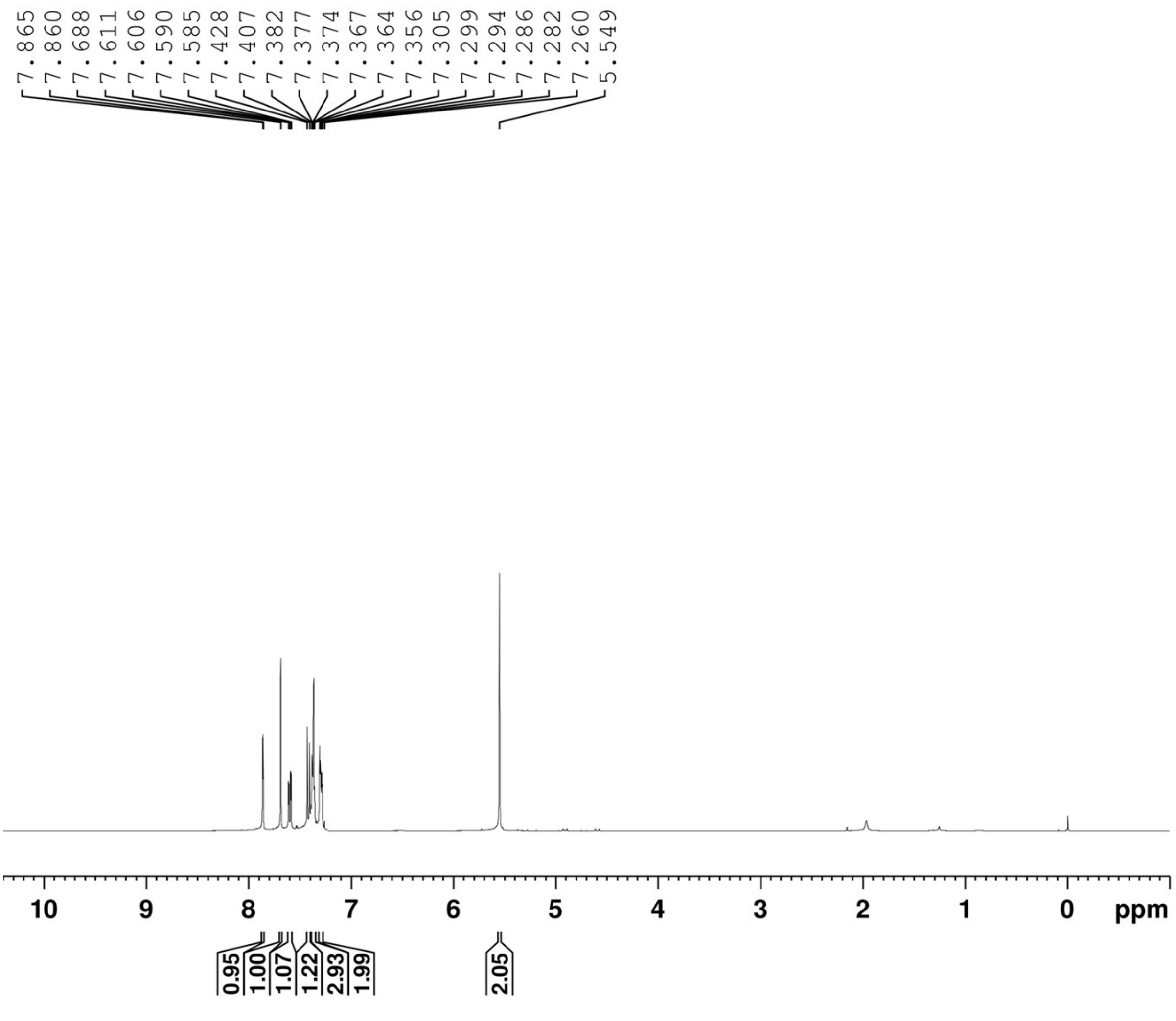

Figure S20. ^{1}H NMR (400 MHz, $CDCl_3$) spectra of compound **g**

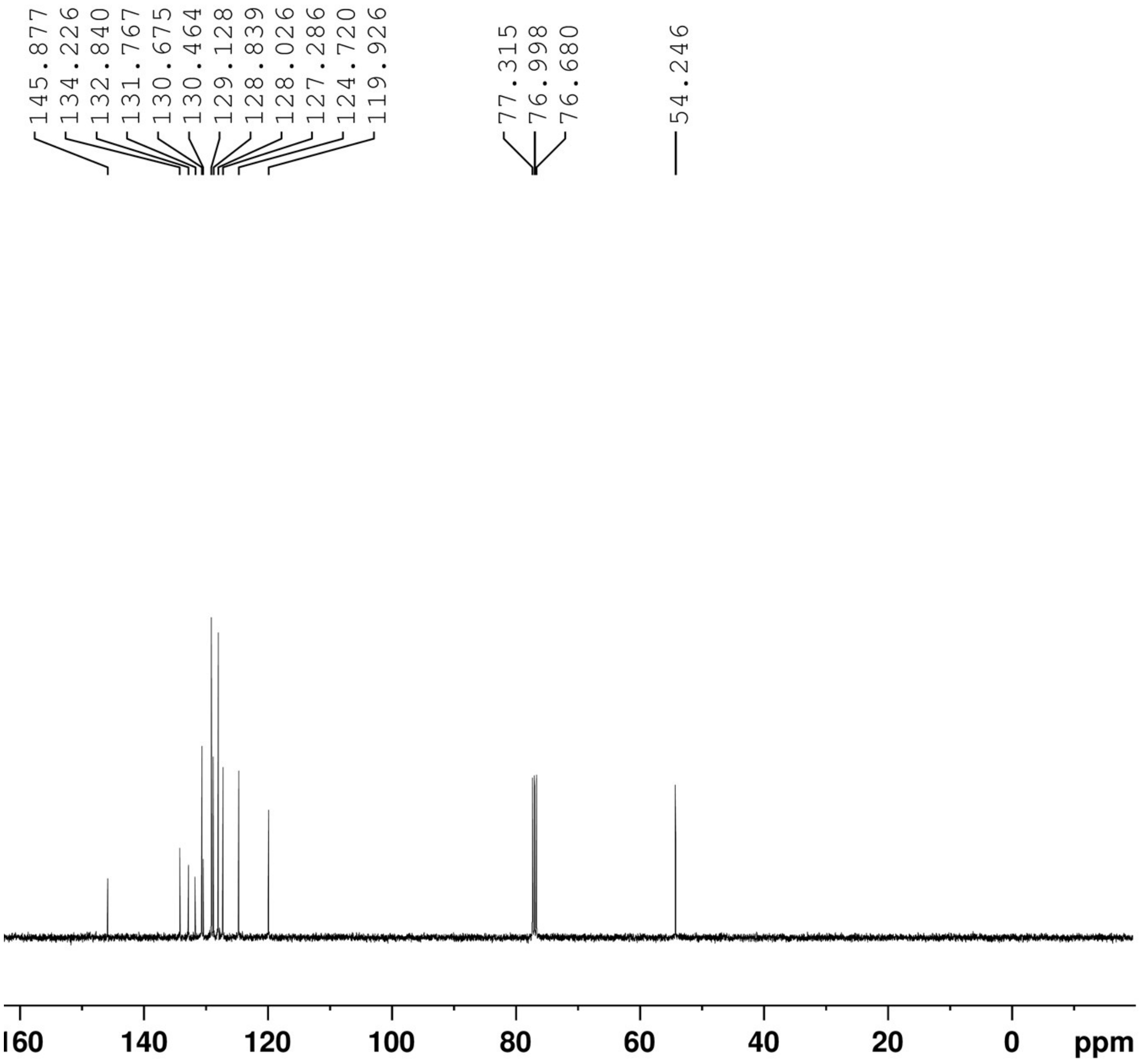

Figure S21. ^{13}C NMR (100 MHz, $CDCl_3$) spectra of compound **g**

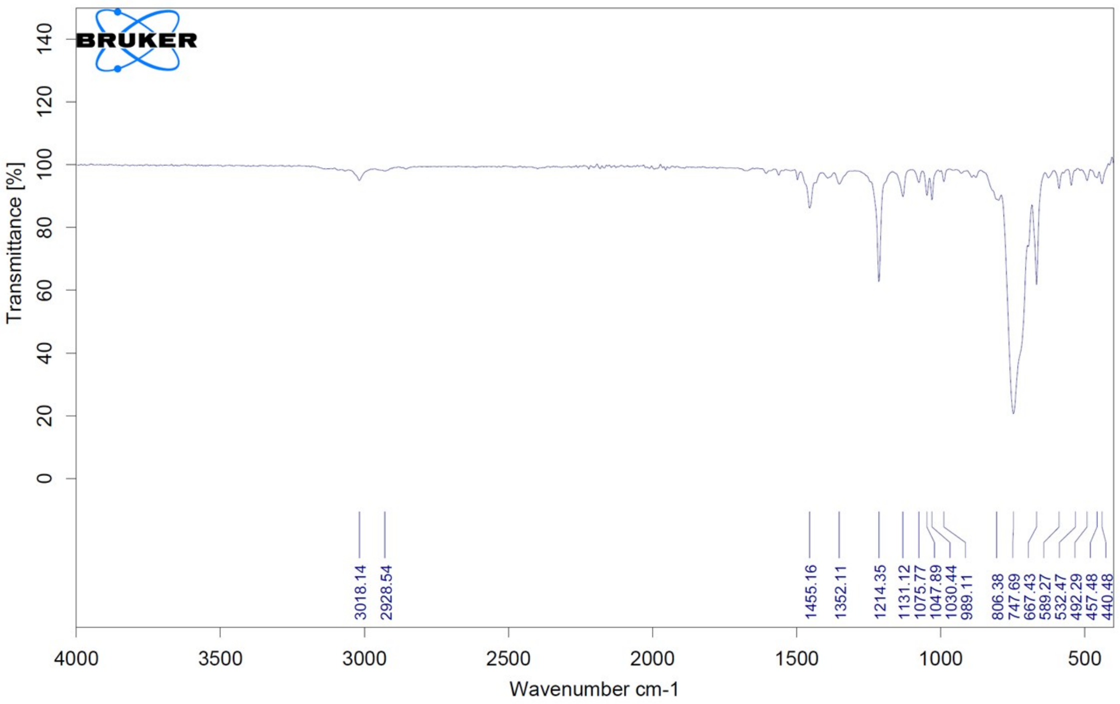

Figure S22. IR spectra of compound **g**

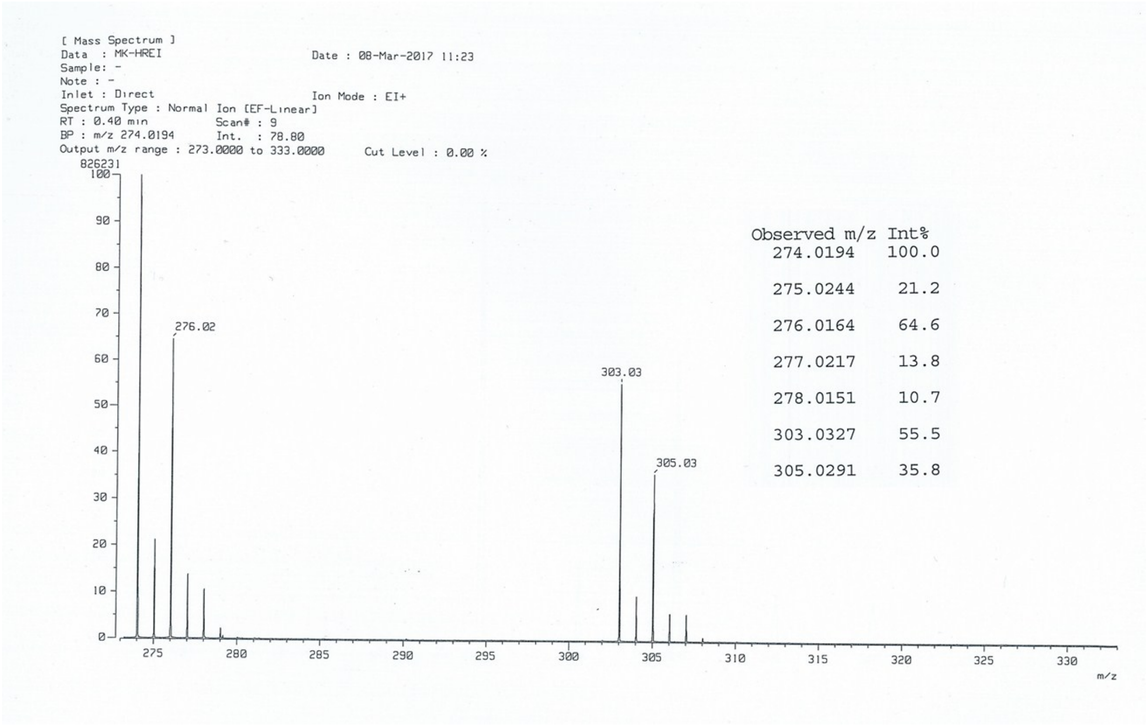

Figure S23. Mass spectra of compound **g**

1-Benzyl-4-(4-(*tert*-butyl)phenyl)-1*H*-1,2,3-triazole(h)[6]

White solid, yield: Cu(I)@IPSi 0.280 g (96%), Cu(II)@IPSi 0.251 g (86%), Cu(I)@pNIPAM-VP 0.245 g (84%), Cu(II)@pNIPAM-VP 0.262 g (90%); R_f = 0.28 (hexane/ethyl acetate = 5:1, v/v); m.p. 117.5-118.4 ^{o}C; ^{1}H NMR (400 MHz, $CDCl_3$): δ 7.73 (d, *J* = 8.4 Hz, 2H), 7.63 (s, 1H), 7.42 (d, *J* = 8.4 Hz, 2H), 7.39-7.29 (m, 5H), 5.57 (s, 2H), 1.33 (s, 9H); ^{13}C NMR (100 MHz, $CDCl_3$): δ 151.28, 148.25, 134.83, 129.14, 128.73, 128.00, 127.75, 125.73, 125.44, 119.25, 54.18, 34.67, 31.30; CAS Number: 1314406-61-5.

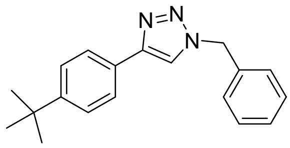

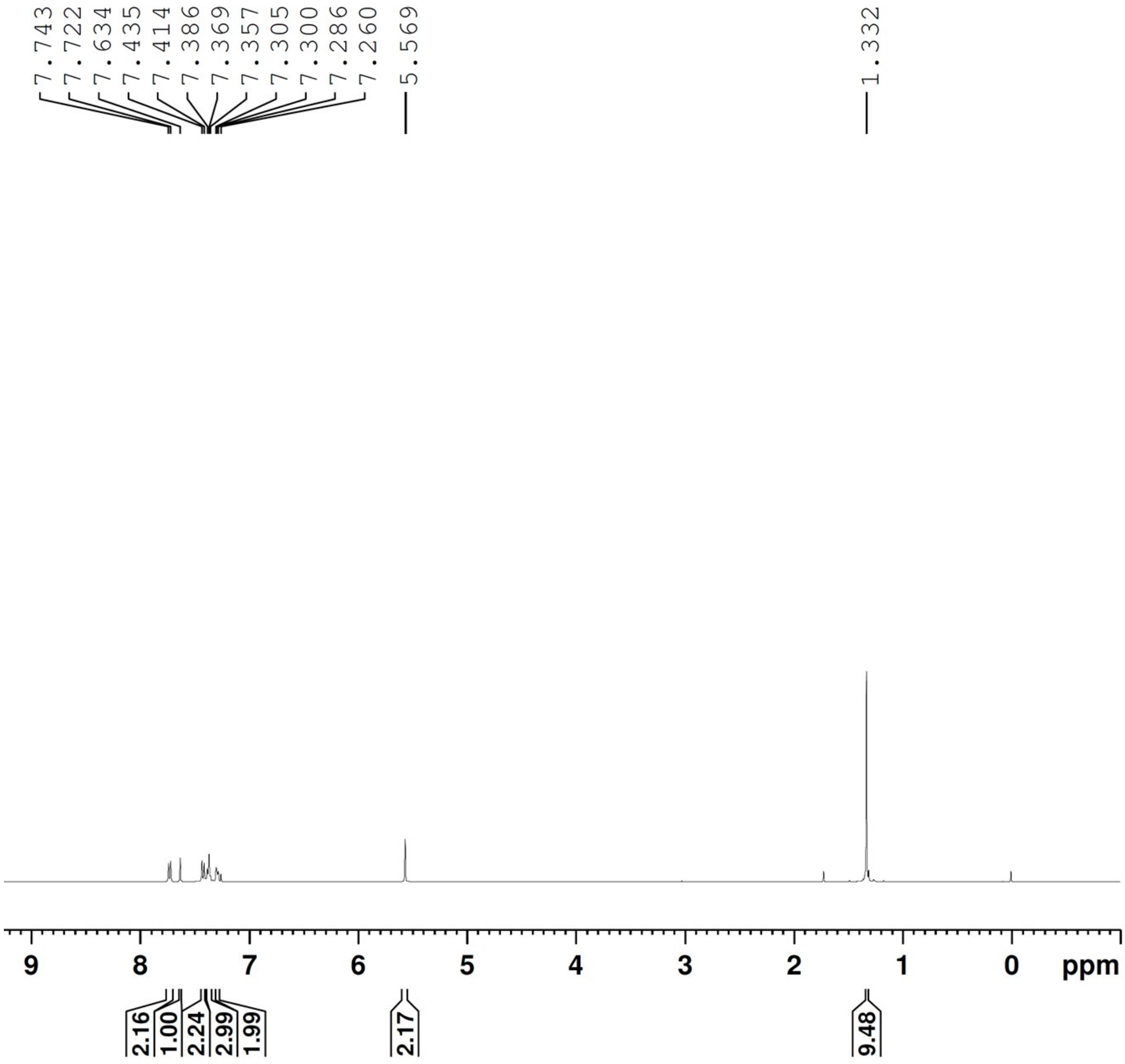

Figure S24. ^{1}H NMR (400 MHz, $CDCl_3$) spectra of compound **h**

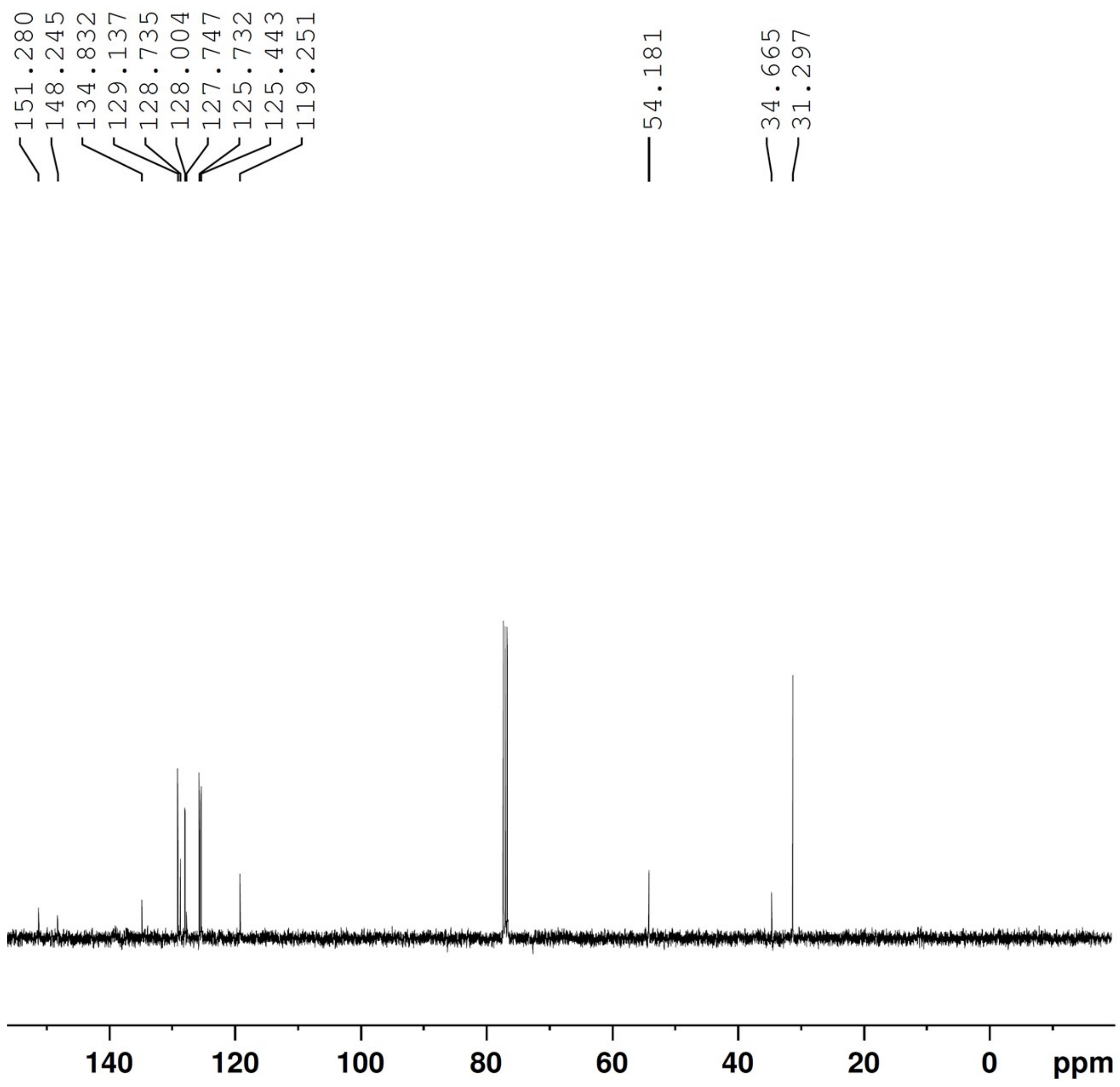

Figure S25. ^{13}C NMR (100 MHz, $CDCl_3$) spectra of compound **h**

1-(4-Nitrobenzyl)-4-phenyl-1*H*-1,2,3-triazole (i)[6]

White solid, yield: Cu(I)@IPSi 0.263 g (94%), Cu(II)@IPSi 0.263 g (94%), Cu(I)@pNIPAM-VP 0.0.202 g (72%), Cu(II)@pNIPAM-VP 0.207 g (74%); R_f = 0.10 (hexane/ethyl acetate = 5:1, v/v); m.p. 157.4-158.9°C; ^{1}H NMR (400 MHz, $CDCl_3$): δ 8.25 (d, *J* = 8.1 Hz, 2H), 7.82 (d, *J* = 8.2 Hz, 2H), 7.75 (s, 1H), 7.46-7.41 (m, 4H), 7.37-7.33 (m, 1H); ^{13}C NMR (100 MHz, $CDCl_3$): δ 148.75, 148.12, 141.76, 130.11, 128.93, 128.56, 128.51, 125.76, 124.36, 119.67, 53.20; CAS Number: 104926-74-1.

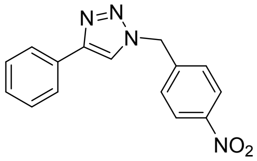

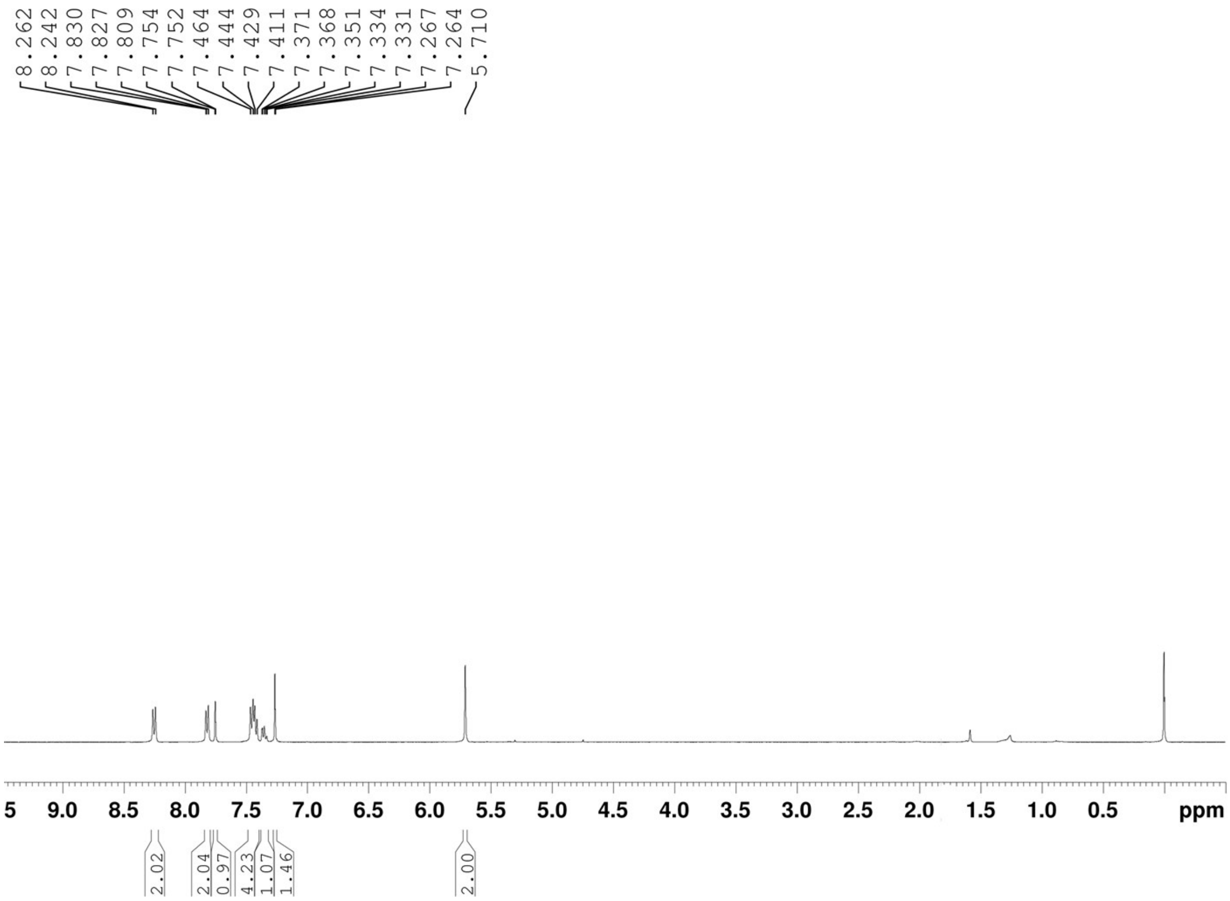

Figure S26. ^{1}H NMR (400 MHz, $CDCl_3$) spectra of compound **i**

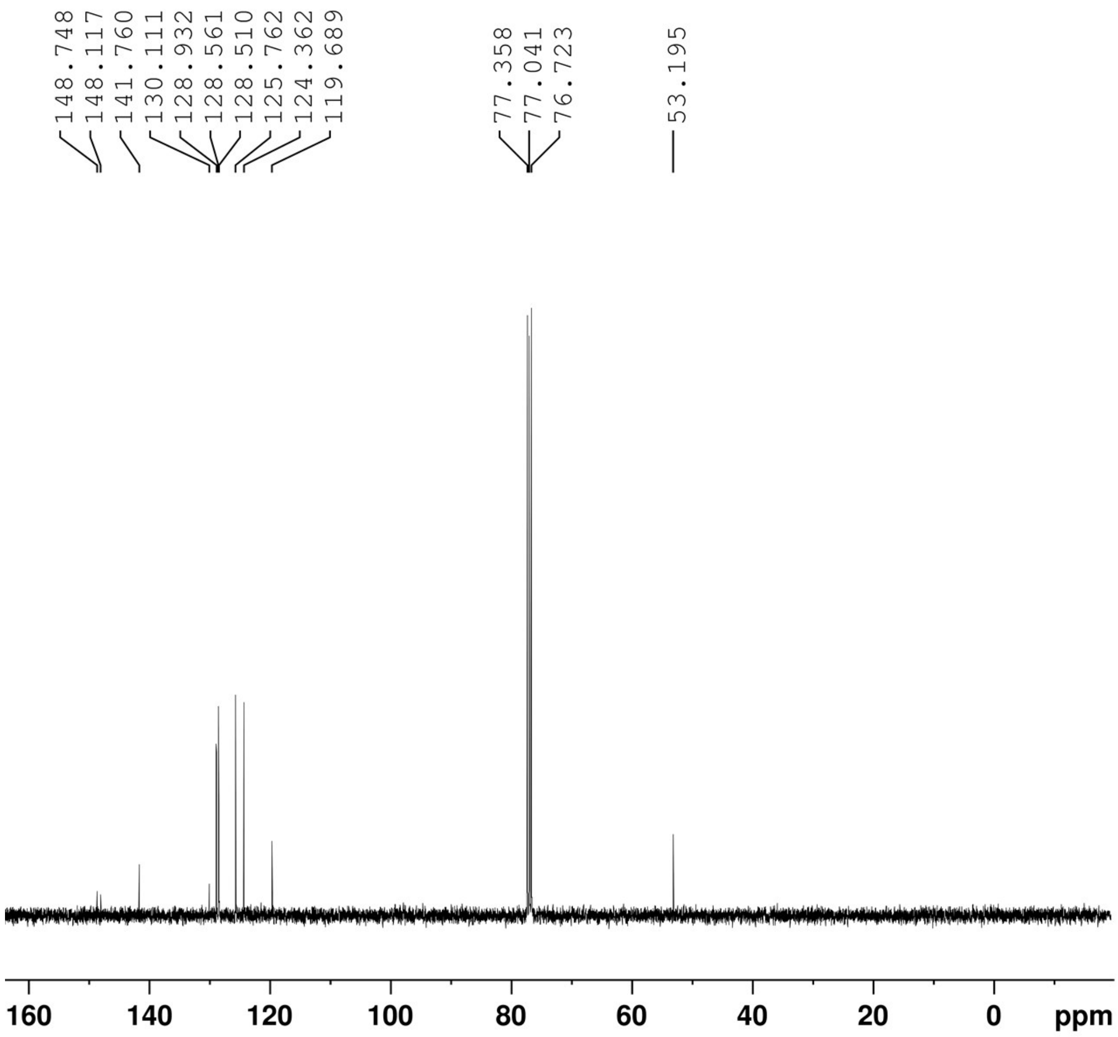

Figure S27. ^{13}C NMR (100 MHz, $CDCl_3$) spectra of compound **i**

4-Phenyl-1-(4-(trifluoromethyl)benzyl)-1*H*-1,2,3-triazole(j)[5]

White solid, yield: Cu(I)@IPSi 0.243 g (80%), Cu(II)@IPSi 0.252 g (83%), Cu(I)@pNIPAM-VP 0.267 g (88%), Cu(II)@pNIPAM-VP 0.227 g (75%); $R_f = 0.23$ (hexane/ethyl acetate = 5:1, v/v); m.p. 130.0-131.9 °C; ^{1}H NMR (400 MHz, $CDCl_3$): δ 7.80 (d, J = 7.2 Hz, 2H), 7.72 (s, 1H), 7.63 (d, J = 8.2 Hz), 7.42-7.38 (m, 4H), 7.34-7.30 (m, 1H), 5.62 (s, 2H); ^{13}C NMR (100 MHz, $CDCl_3$): δ 148.53, 138.70, 131.02 (q, J = 32.7), 130.26, 128.90, 128.39, 128.19, 126.14 (q, J = 3.7), 125.74, 123.80 (q, J = 270.6), 119.69, 53.52); CAS Number: 1252678-76-4.

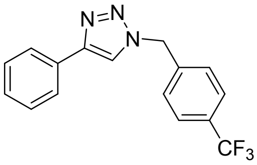

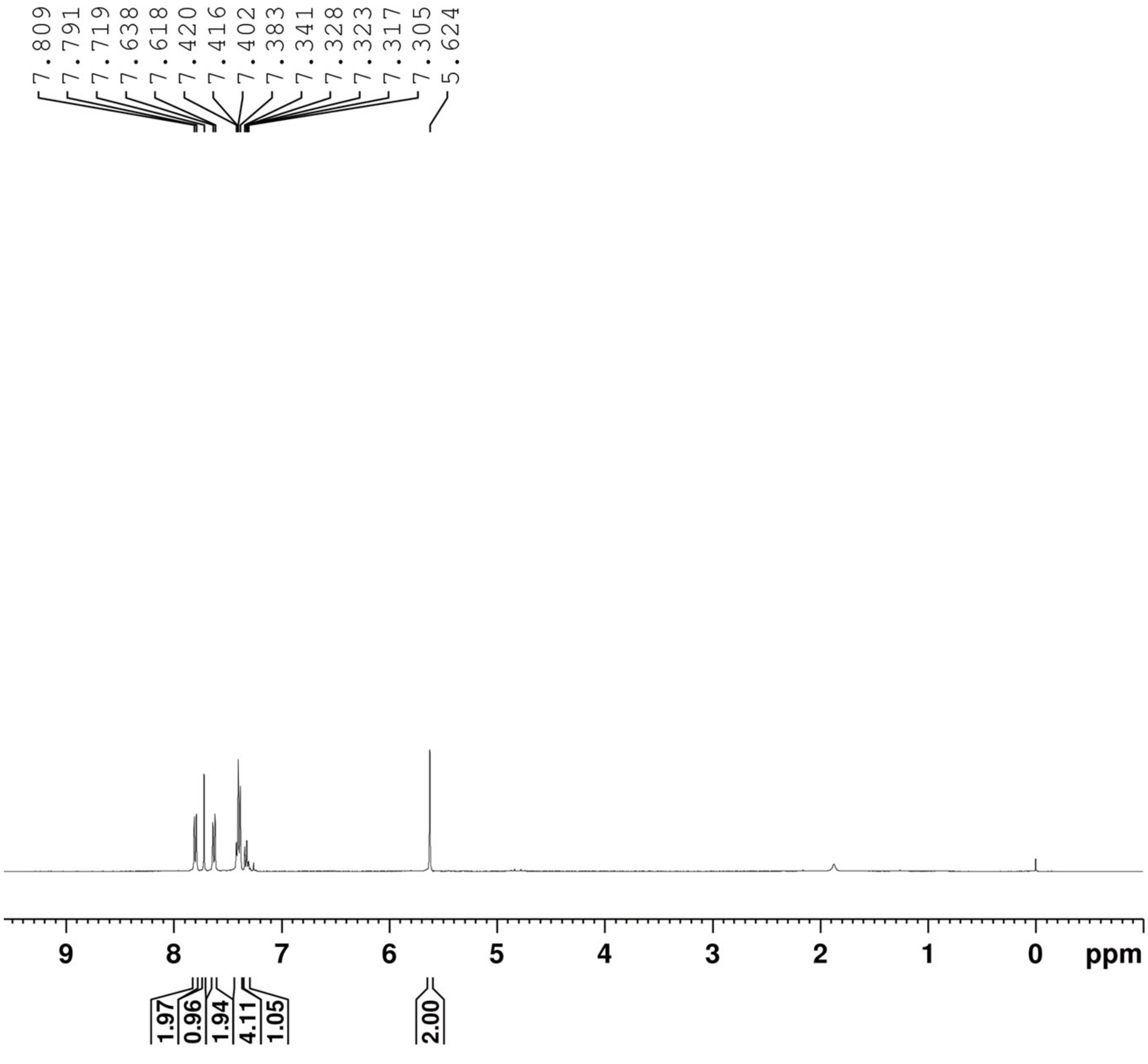

Figure S28. ^{1}H NMR (400 MHz, $CDCl_3$) spectra of compound **j**

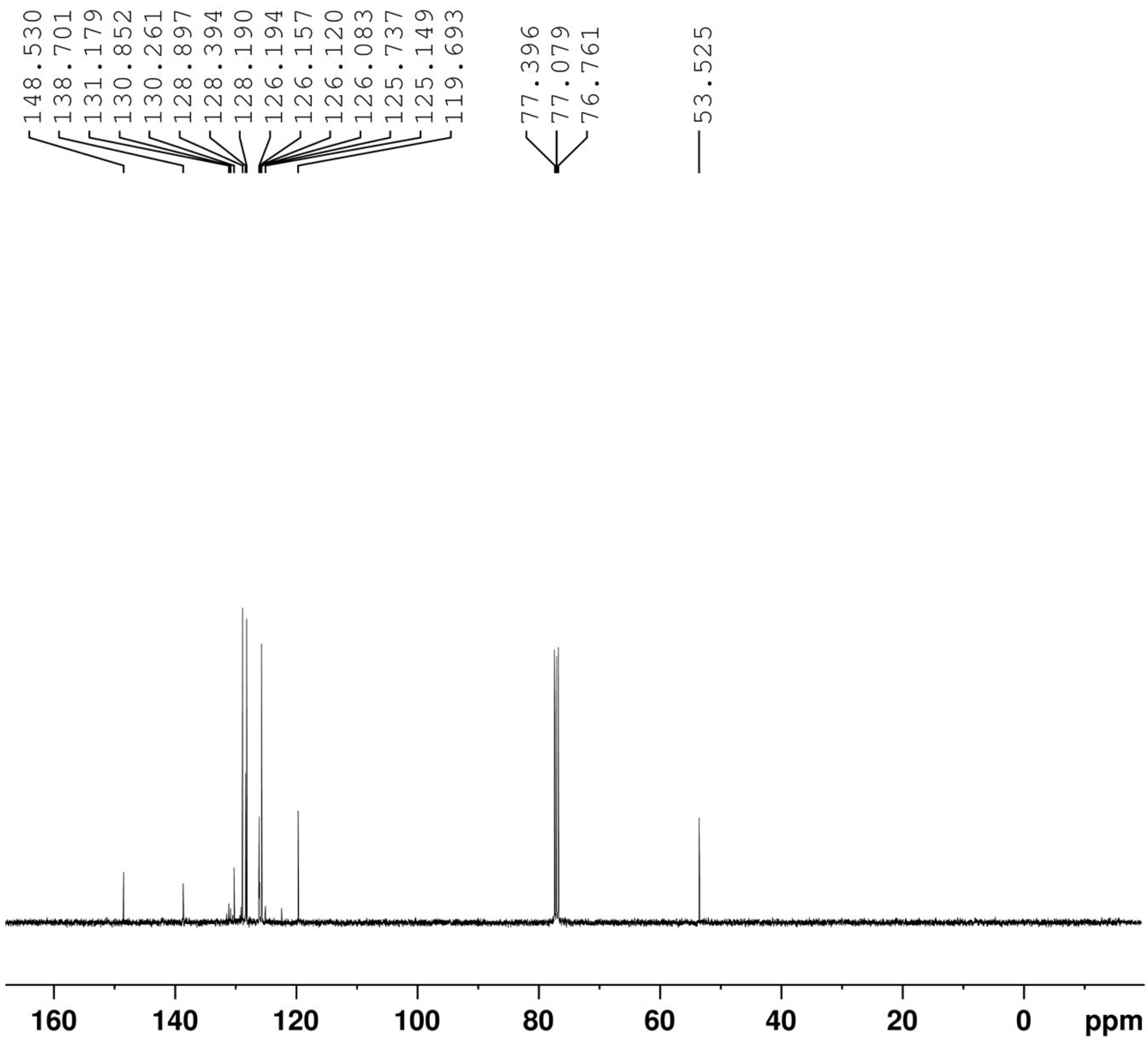

Figure S29. ^{13}C NMR (100 MHz, $CDCl_3$) spectra of compound **j**

1-(4-Chlorobenzyl)-4-phenyl-1*H*-1,2,3-triazole(k)[7]

White solid, yield: Cu(I)@IPSi 0.243 g (90%), Cu(II)@IPSi 0.243 g (90%), Cu(I)@pNIPAM-VP 0.167 g (62%), Cu(II)@pNIPAM-VP 0.237 g (88%); R_f = 0.25 (hexane/ethyl acetate = 3:1, v/v); m.p. 144.0-145.2°C; ^{1}H NMR (400 MHz, $CDCl_3$): δ 7.79 (d, *J* = 7.0 Hz, 2H), 7.67 (s, 1H), 7.42-7.30 (m, 5H), 7.23 (d, *J* = 8.6 Hz, 2H), 5.53 (s, 2H); ^{13}C NMR (100 MHz, $CDCl_3$): δ 148.42, 134.87, 133.19, 130.36, 130.36, 129.39, 128.87, 128.31, 125.73, 119.44, 53.50; CAS Number: 126800-07-5.

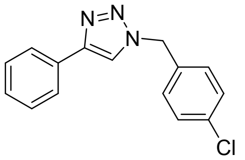

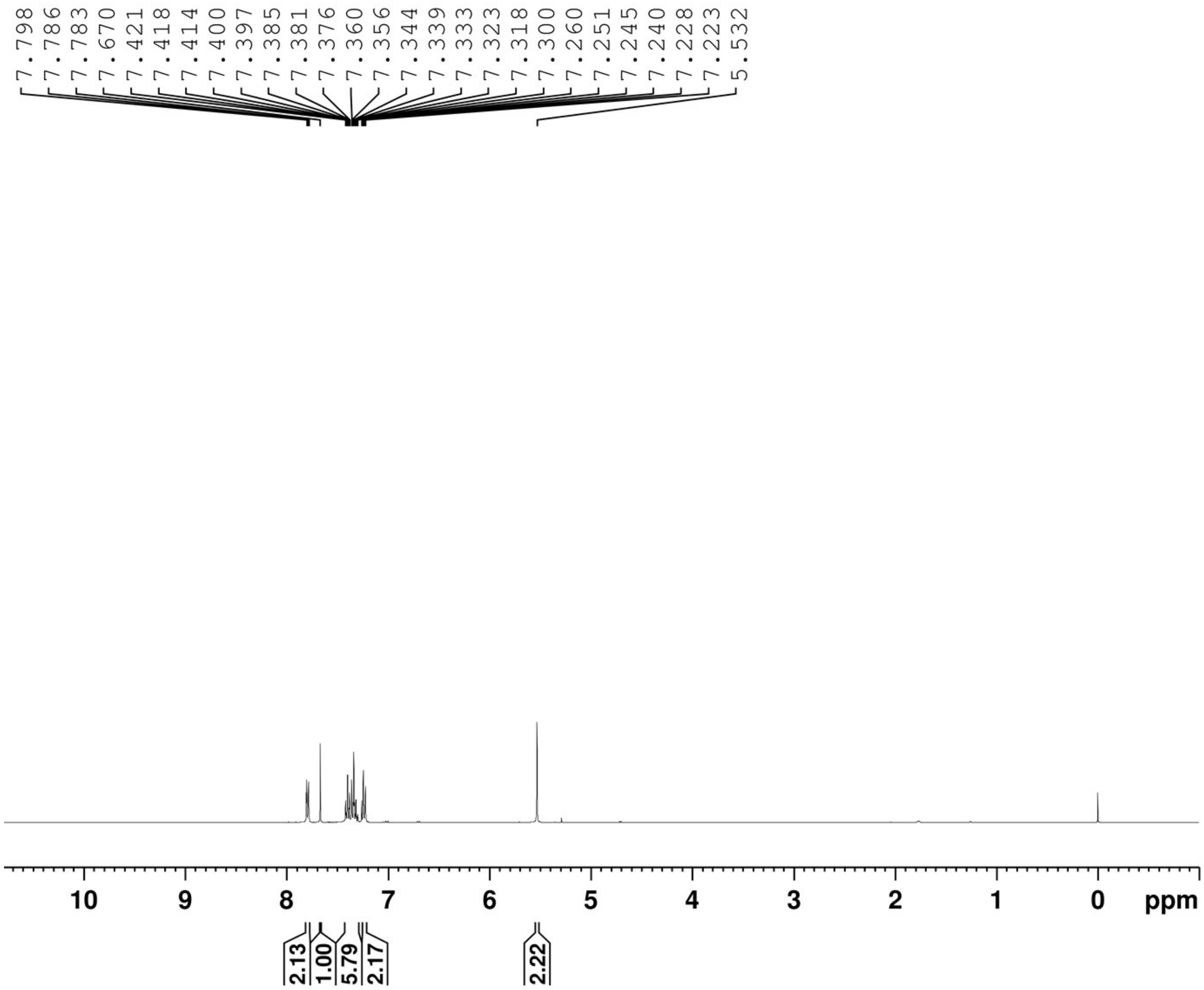

Figure S30. ^{1}H NMR (400 MHz, $CDCl_3$) spectra of compound **k**

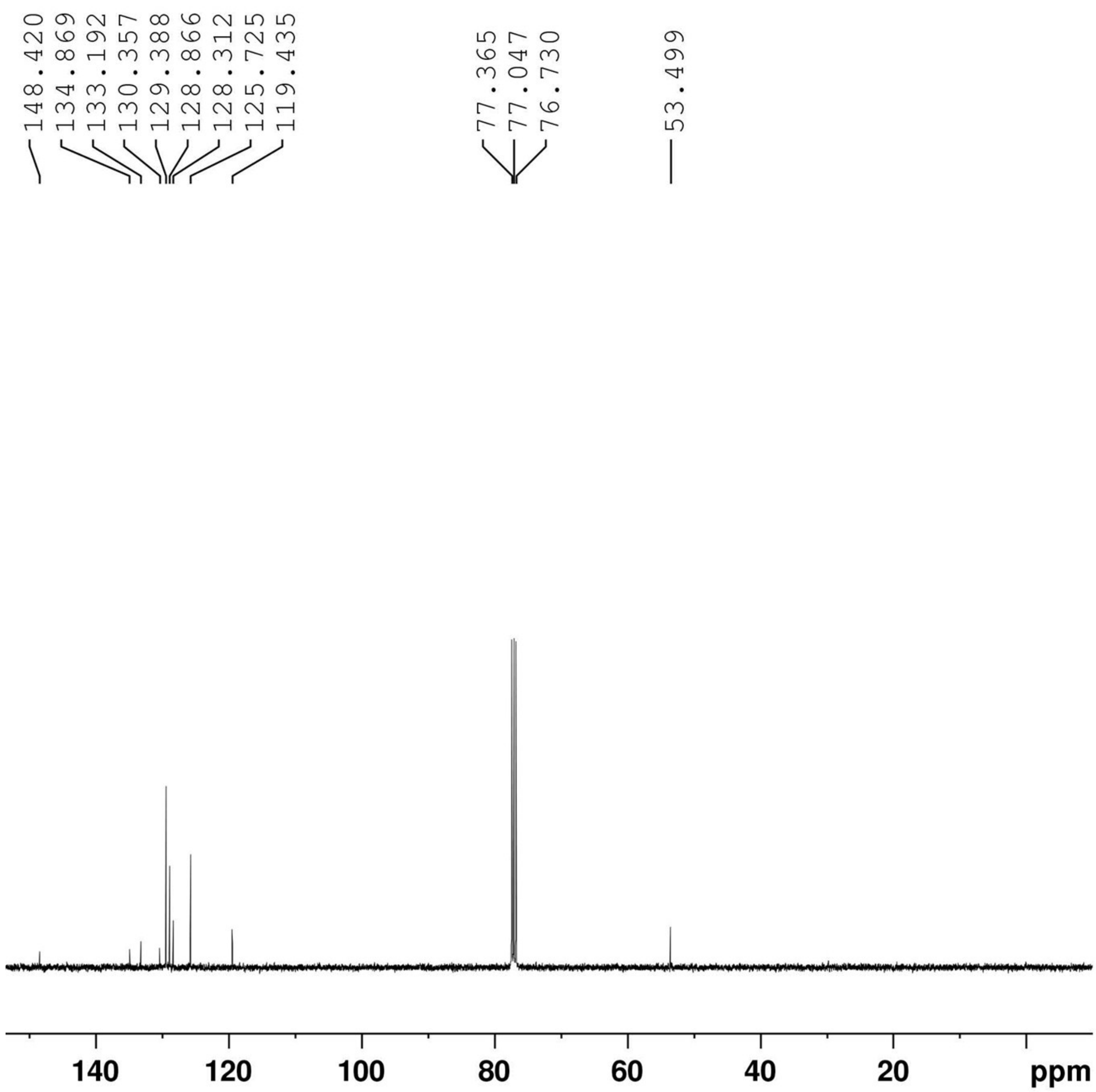

Figure S31. ^{13}C NMR (100 MHz, $CDCl_3$) spectra of compound **k**

1-(3-Methoxybenzyl)-4-phenyl-1*H*-1,2,3-triazole (l)[8]

White solid, yield: Cu(I)@IPSi 0.255 g (96%), Cu(II)@IPSi 0.225 g (96%), Cu(I)@pNIPAM-VP 0.236 g (89%), Cu(II)@pNIPAM-VP 0.233 g (88%); R_f = 0.25 (hexane/ethyl acetate = 5:1, v/v); m.p. 104.5-106°C; ^{1}H NMR (400 MHz, $CDCl_3$): δ 7.80 (d, *J* = 7.3 Hz, 2H), 7.67 (s, 1H), 7.40 (t, *J* = 8.0, 7.3 Hz, 2H), 7.34-7.29 (m, 2H), 6.90 (d, *J* = 8.1 Hz, 2H), 6.84 (s, 1H), 5.55 (s, 2H), 3.79 (s, 3H); ^{13}C NMR (100 MHz, $CDCl_3$): 160.22, 146.86, 135.82, 133.99, 130.35, 130.16, 125.83, 125.79, 120.32, 120.26, 114.31, 113.84, 55.34, 54.33; CAS Number: 126800-01-9.

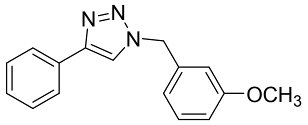

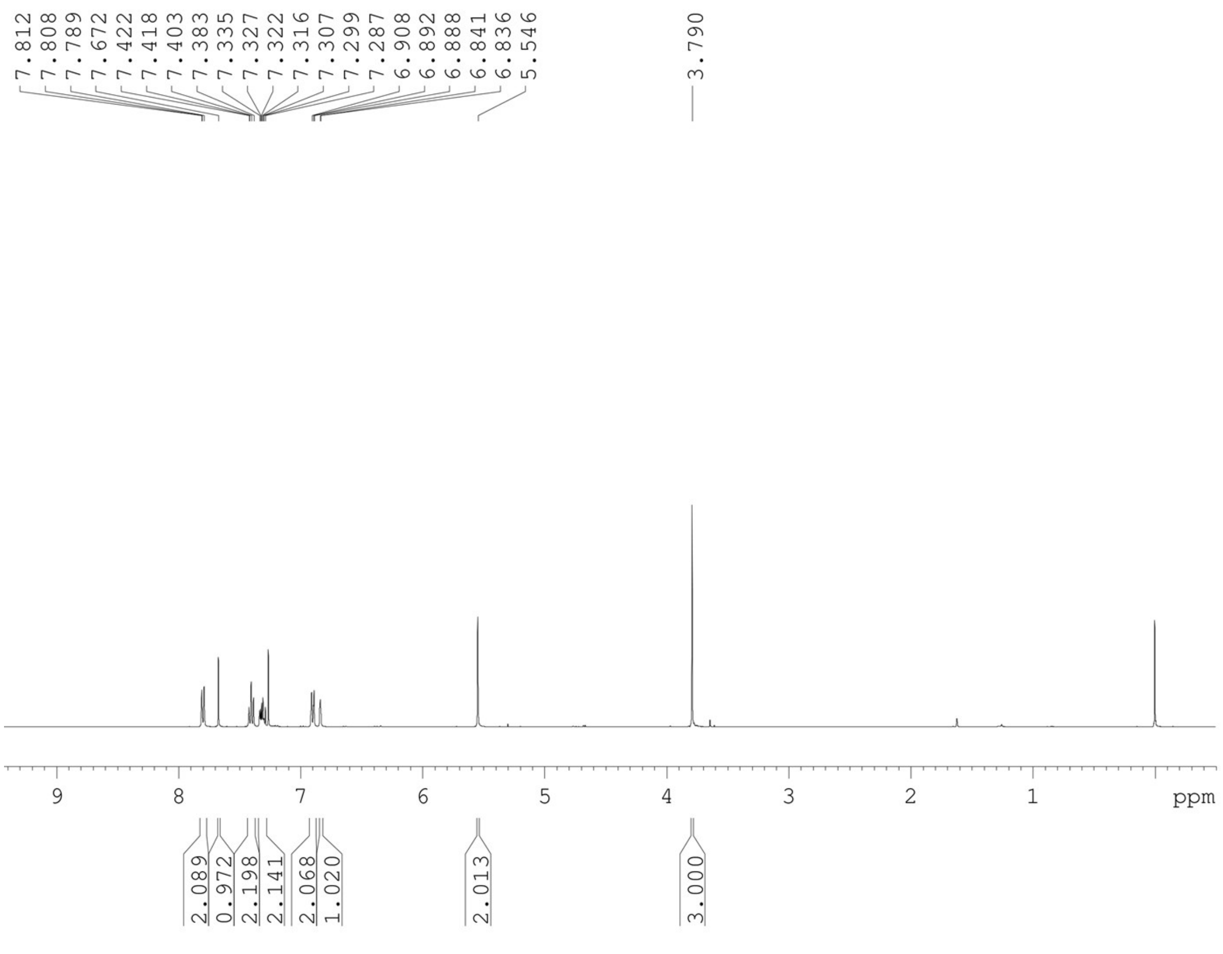

Figure S32. ^{1}H NMR (400 MHz, $CDCl_3$) spectra of compound **l**

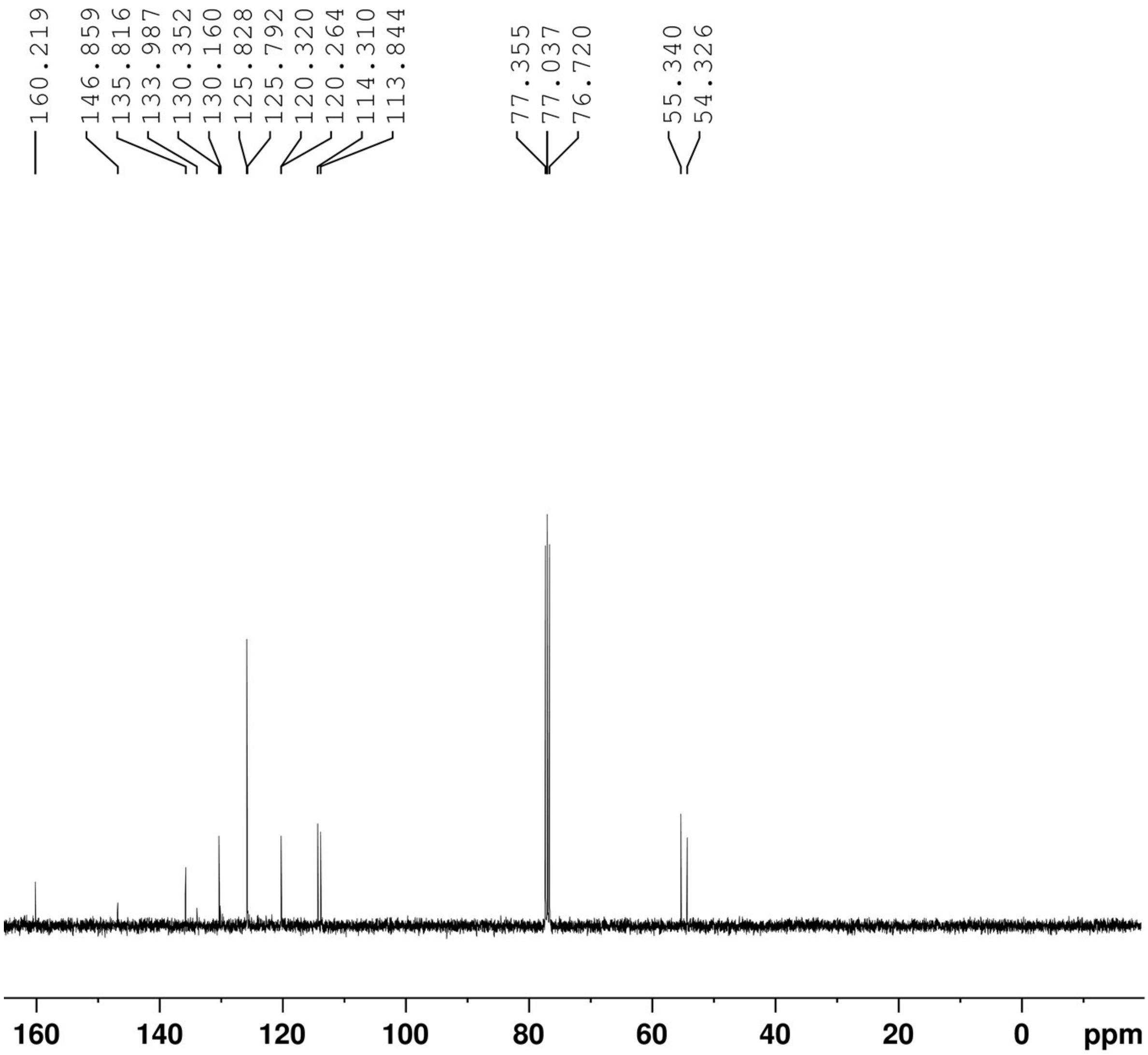

Figure S33. ^{13}C NMR (100 MHz, $CDCl_3$) spectra of compound **l**

1-(4-Methylbenzyl)-4-phenyl-1*H*-1,2,3-triazole (m)[9]

White solid, yield: Cu(I)@IPSi 0.209 g (84%), Cu(II)@IPSi 0.209 g (84%), Cu(I)@pNIPAM-VP 0.175 g (70%), Cu(II)@pNIPAM-VP 0.182g (73%); R_f = 0.25 (hexane/ethyl acetate = 3:1, v/v); m.p. 110.1-111.4°C; ^{1}H NMR (400 MHz, $CDCl_3$): δ 7.79 (d, J = 7.2 Hz, 2H), 7.63 (s, 1H), 7.41-7.29 (m, 3H), 7.23-7.18 (m, 4H), 5.53 (s, 2H), 2.36 (s, 3H); ^{13}C NMR (100 MHz, $CDCl_3$): δ 148.16, 138.75, 131.67, 130.62, 129.83, 128.8, 128.15, 128.12, 125.70, 119.39, 54.10, 21.19; CAS Number:126800-02-0.

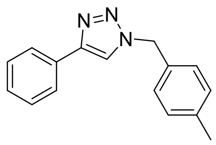

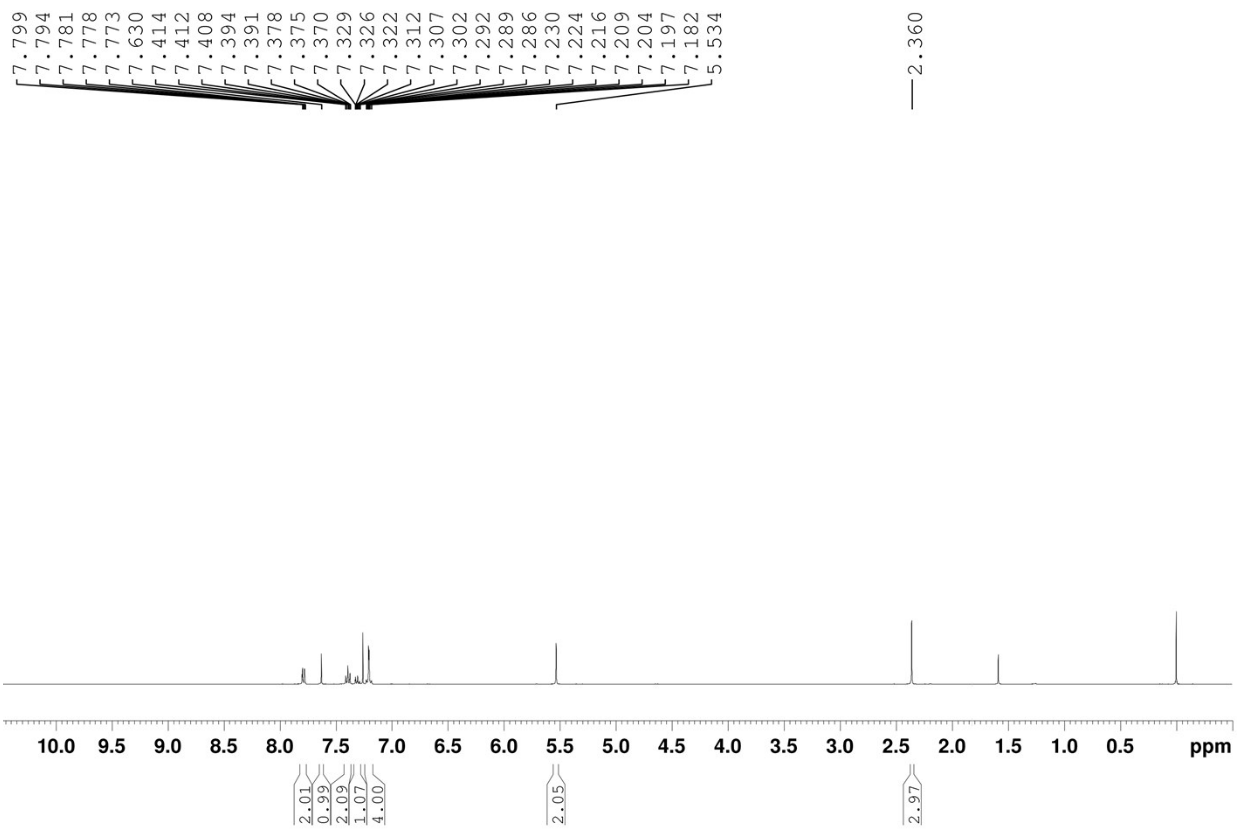

Figure S34. ^{1}H NMR (400 MHz, $CDCl_3$) spectra of compound **m**

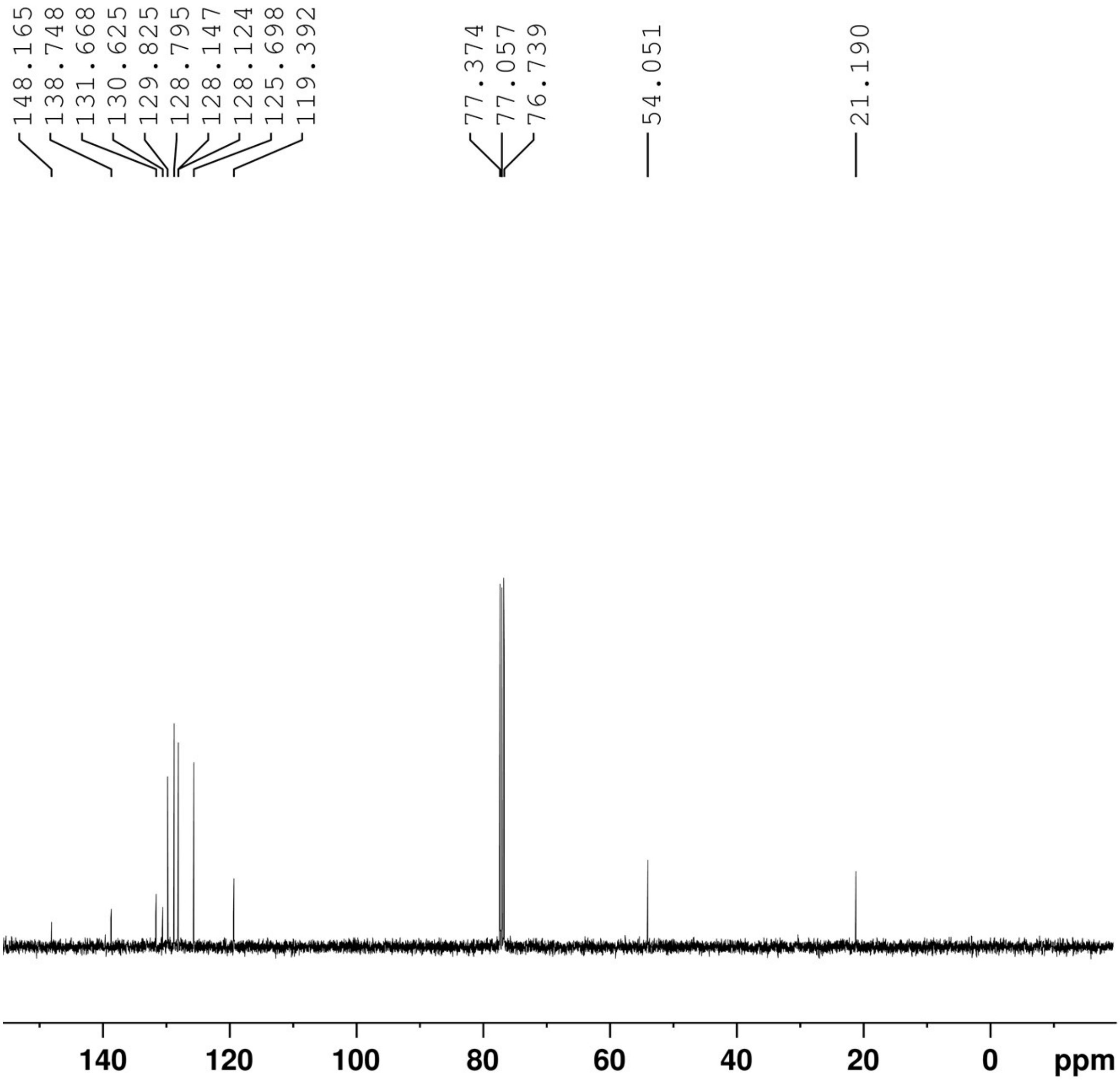

Figure S35. ^{13}C NMR (100 MHz, $CDCl_3$) spectra of compound **m**

1-(4-Chlorobenzyl)-4-(4-methoxyphenyl)-1*H*-1,2,3-triazole (n)[10]

White solid, yield: Cu(I)@IPSi 0.273 g (91%), Cu(II)@IPSi 0.273 g (91%), Cu(I)@pNIPAM-VP 0.276 g (92%), Cu(II)@pNIPAM-VP 0.270 g (90%); R_f = 0.20 (hexane/ethyl acetate = 5:1, v/v); m.p. 159.1-160.7 °C; ^{1}H NMR (400 MHz, $CDCl_3$): δ 7.71 (d, *J* = 8.8 Hz, 2H), 7.58 (s, 1H), 7.34 (d, *J* = 8.4 Hz, 2H), 7.22 (d, *J* = 8.4 Hz, 2H), 6.92 (d, *J* = 8.8 Hz, 2H), 5.51 (s, 2H), 3.82 (s, 3H); ^{13}C NMR (100 MHz, $CDCl_3$): δ 159.66, 148.25, 134.76, 133.32, 129.36, 129.33, 127.02, 123.10, 118.69, 114.24, 55.33, 53.42; CAS Number: 1839568-93-2.

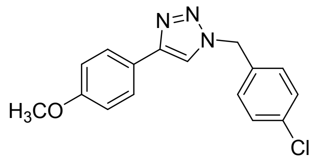

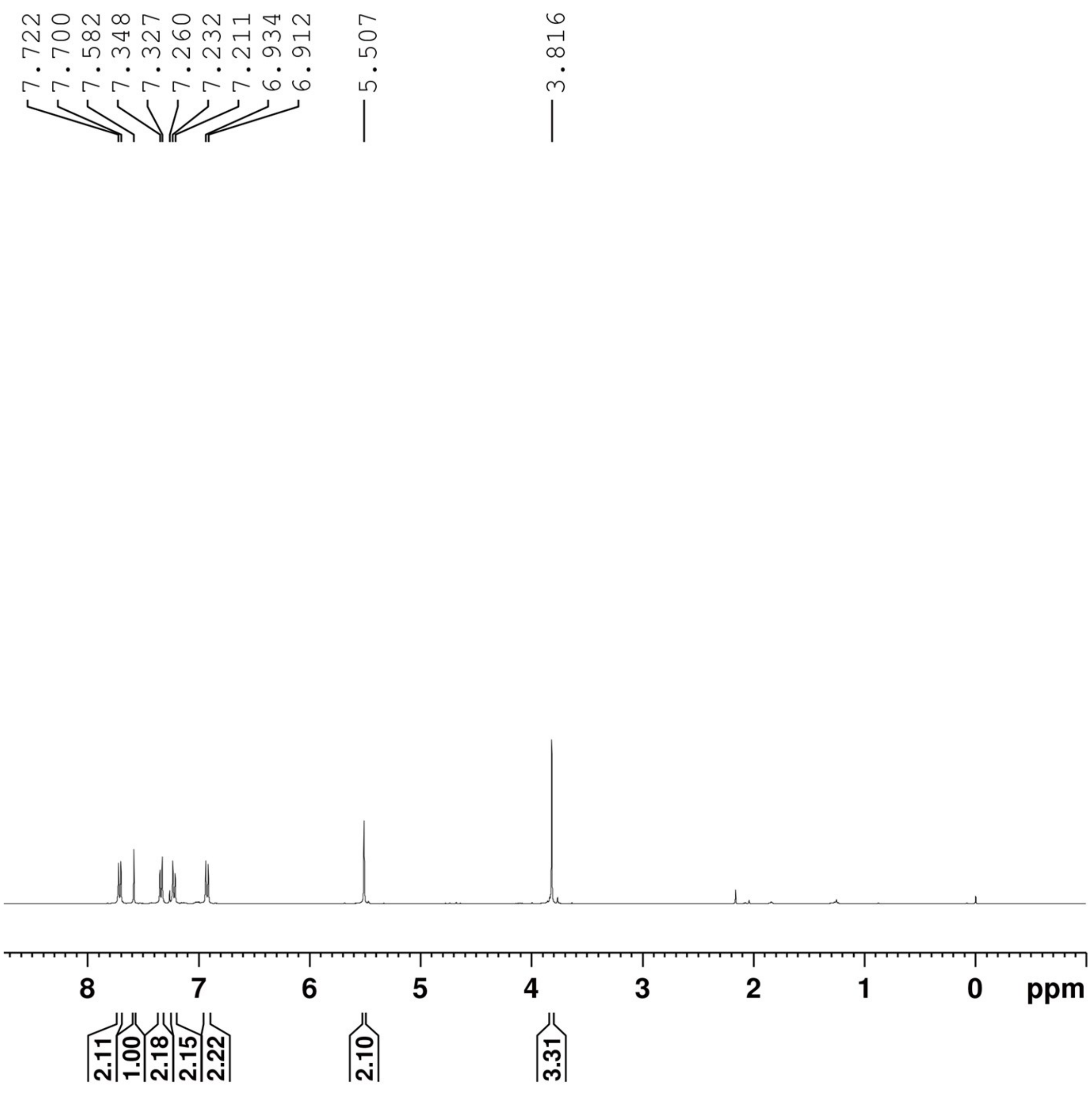

Figure S36. ^{1}H NMR (400 MHz, $CDCl_3$) spectra of compound **n**

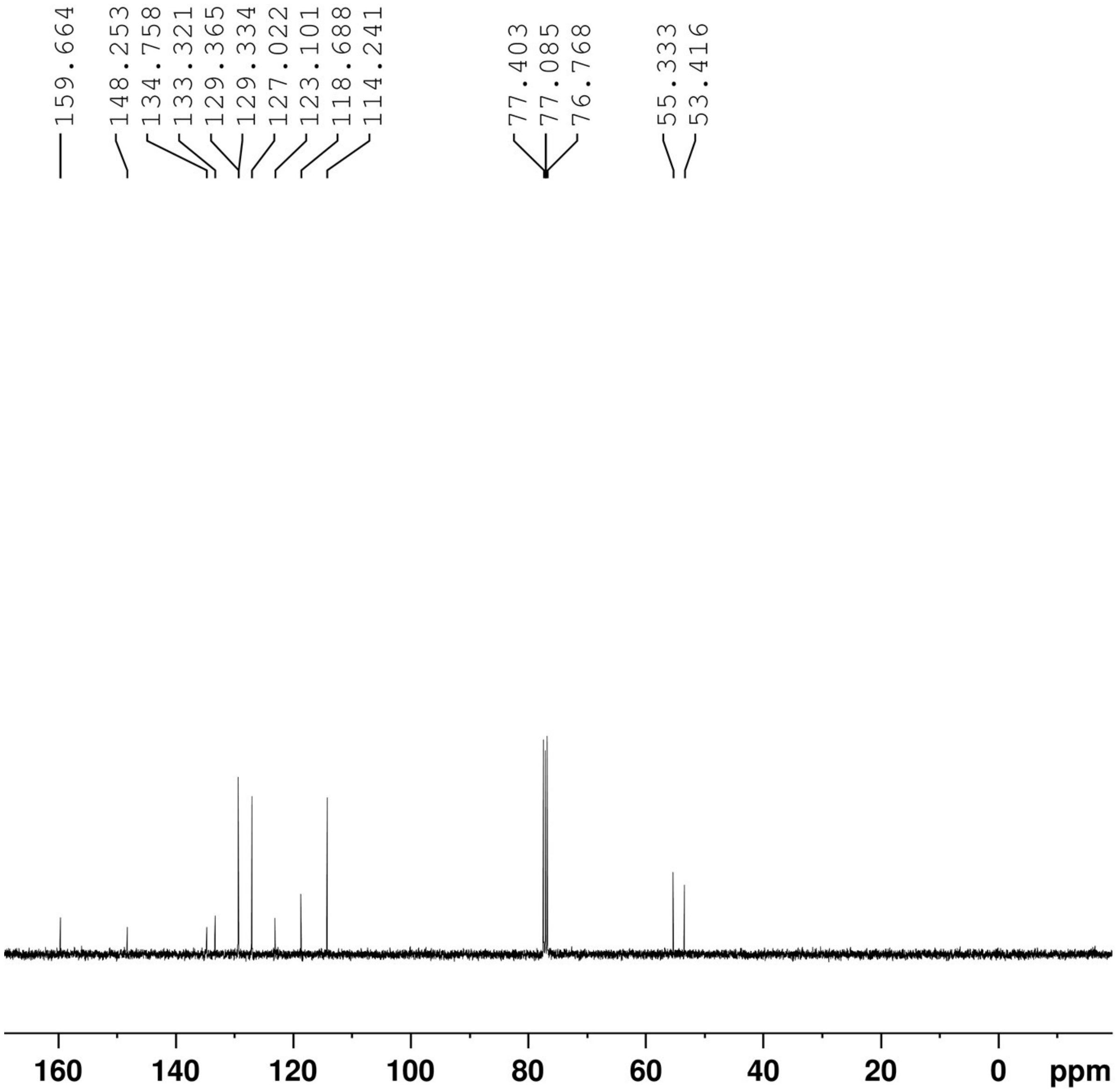

Figure S37. ^{13}C NMR (100 MHz, $CDCl_3$) spectra of compound **n**

1-(3-Methoxybenzyl)-4-(4-methoxyphenyl)-1*H*-1,2,3-triazole (o)[11]

White solid, yield: Cu(I)@IPSi 0.266 g (90%), Cu(II)@IPSi 0.275 g (93%), Cu(I)@pNIPAM-VP 0.266 g (90%), Cu(II)@pNIPAM-VP 0.272 g (92%); R_f = 0.15 (hexane/ethyl acetate = 5:1, v/v); m.p. 133.8-134.9 ^{o}C; ^{1}H NMR (400 MHz, $CDCl_3$): δ 7.72 (d, J = 8.8 Hz, 2H), 7.59 (s, 1H), 7.29 (t, J = 8.0 Hz, 1H), 6.92 (d, J = 8.8 Hz, 2H), 6.88 (d, J = 8.4 Hz, 2H), 6.82 (t, J = 1.8 Hz, 1H), 5.51 (s, 2H), 3.81 (s, 3H), 3.77 (s, 3H); ^{13}C NMR (100 MHz, $CDCl_3$): δ 160.12, 159.58, 148.09, 136.24, 130.22, 127.01, 123.28, 120.25, 118.76, 114.52, 114.20, 113.62, 53.33, 53.11, 54.13; CAS Number: 1135334-77-8.

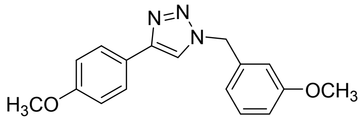

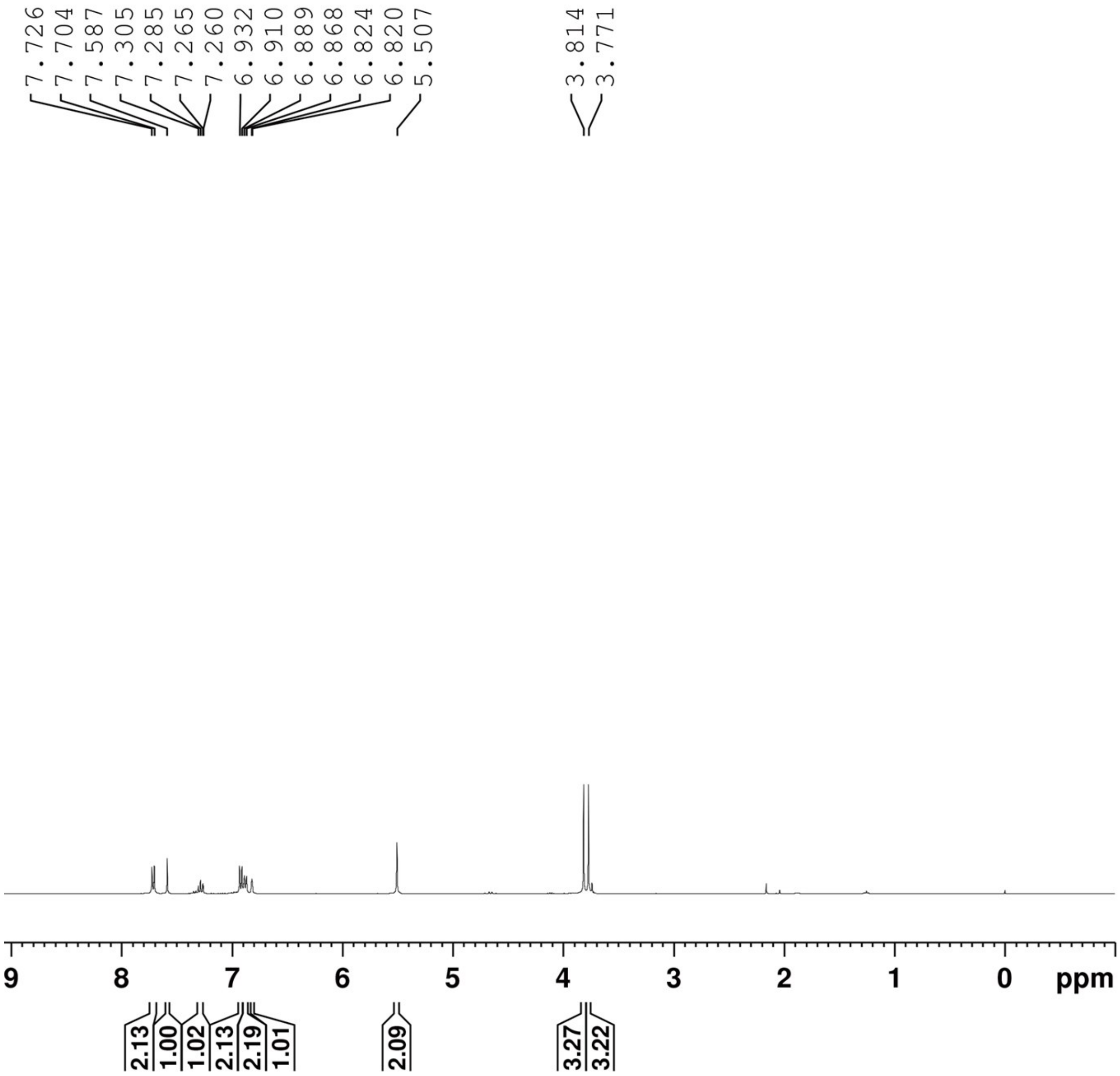

Figure S38. ^{1}H NMR (400 MHz, $CDCl_3$) spectra of compound **o**

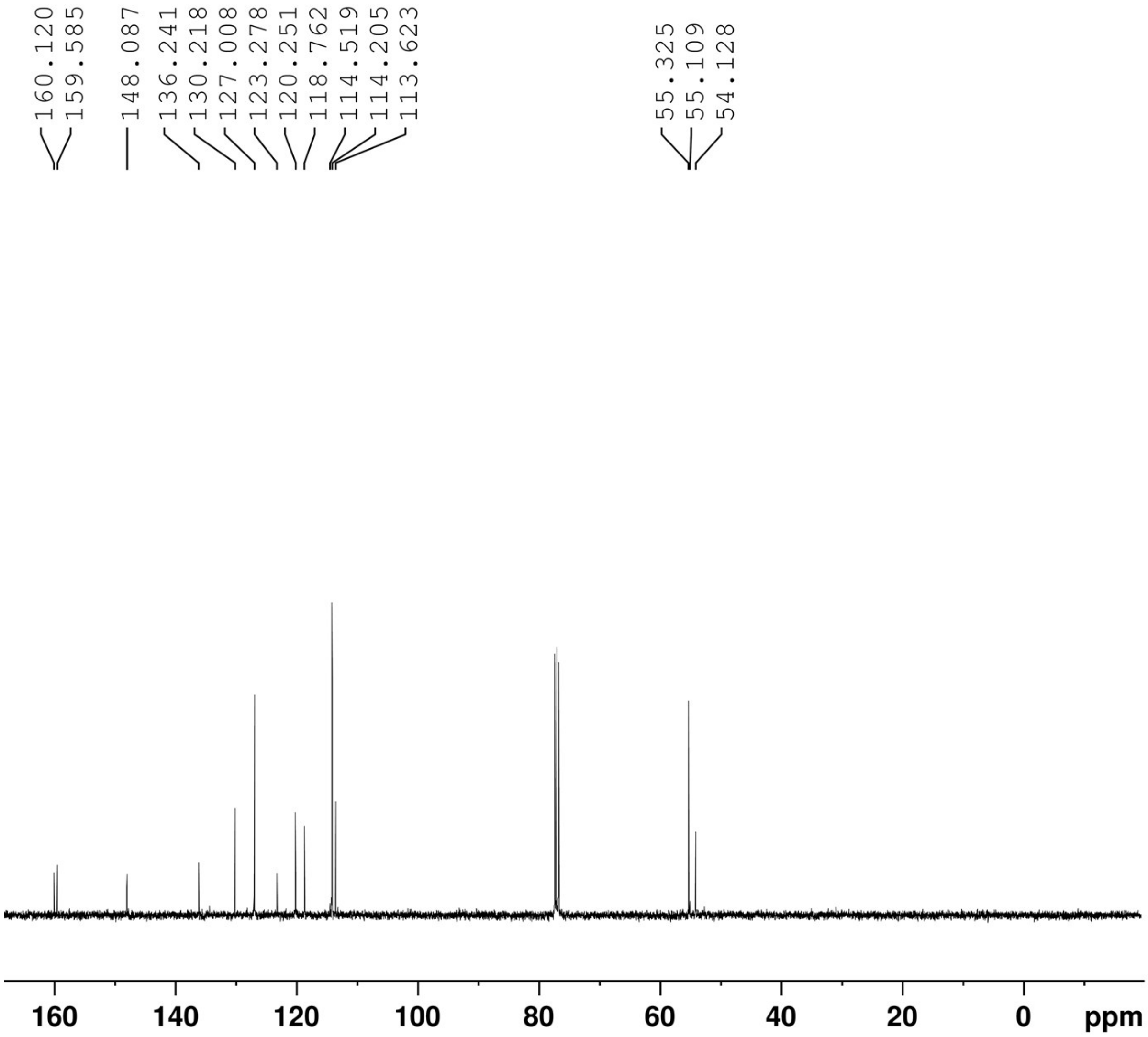

Figure S39. ^{13}C NMR (100 MHz, $CDCl_3$) spectra of compound **o**

1-(4-Chlorobenzyl)-4-(4-(trifluoromethyl)phenyl)-1*H*-1,2,3-triazole (p)

White solid, yield: Cu(I)@IPSi 0.321g (95%), Cu(II)@IPSi 0.317 g (94%), Cu(I)@pNIPAM-VP 0.307 g (91%), Cu(II)@pNIPAM-VP 0.314 g (93%); R_f = 0.20 (hexane/ethyl acetate = 5:1, v/v); m.p. 159.4-160.7 °C; ^{1}H NMR (400 MHz, $CDCl_3$): δ 7.91 (d, *J* = 8.1 Hz, 2H) 7.74 (s, 1H), 7.66 (d, *J* = 8.2 Hz, 2H), 7.38 (d, *J* = 8.4 Hz, 2H), 7.26 (d, *J* = 8.4 Hz, 2H), 5.56 (s, 2H); ^{13}C NMR (100 MHz, $CDCl_3$): δ 146.99, 135.03, 133.77, 132.84, 130.08 (q, *J* = 32 Hz), 129.45, 129.42, 125.82 (q, *J* = 2.88), 125.81, 124.02 (q, *J* = 270.2), 120.13. 53.60; IR(KBr): 3105, 3020, 1920, 2850, 1621, 1325, 1229, 1162, 1125, 1064, 1015, 744, 668 cm^{-1}; HRMS(EI): *m/z* [M]$^+$ for $C_{16}H_{11}ClF_3N_3$, calculated: 337.0594, found: 337.0591.

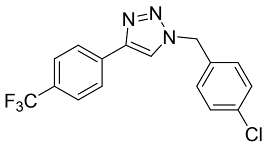

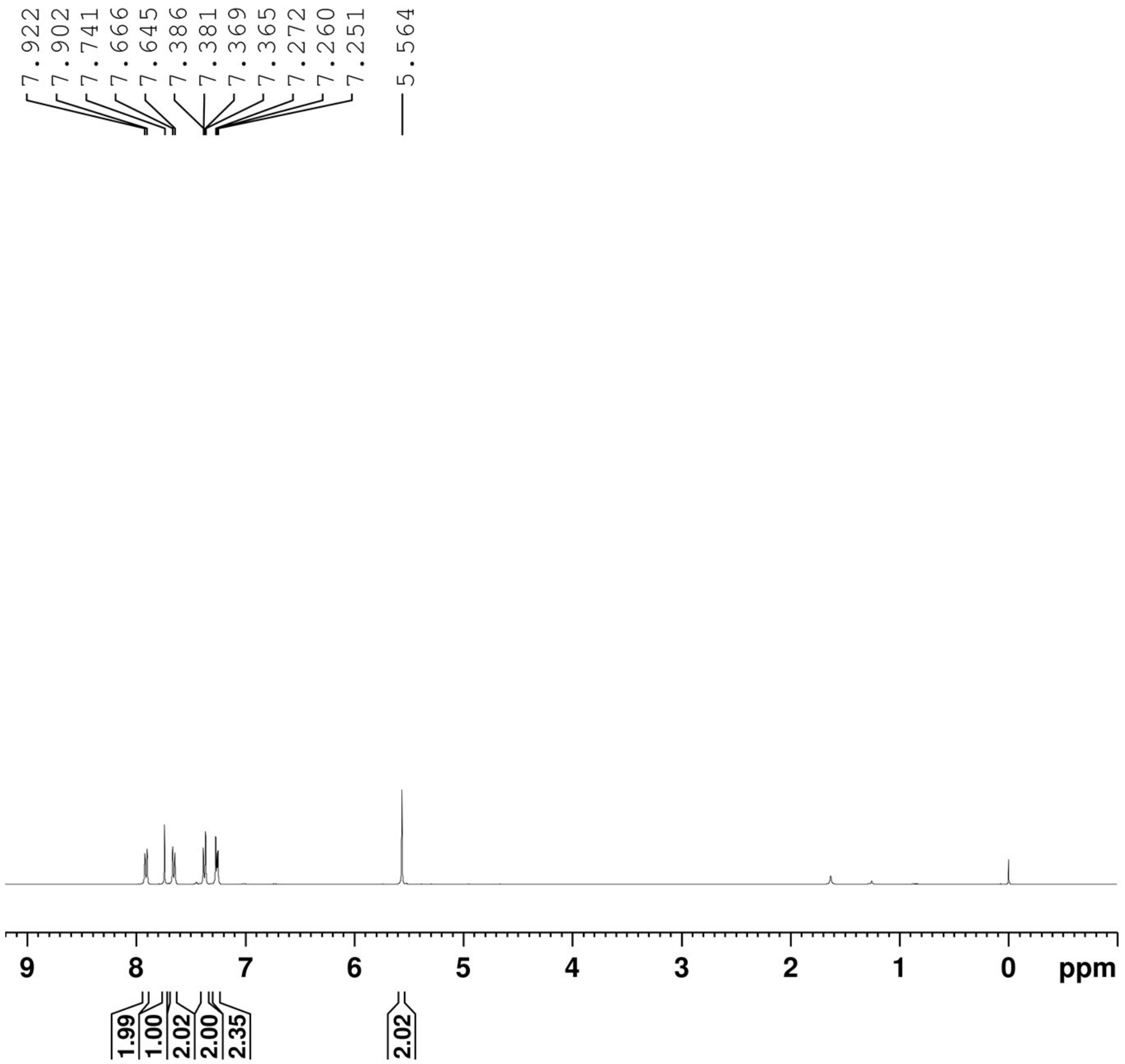

Figure S40. ^{1}H NMR (400 MHz, $CDCl_3$) spectra of compound **p**

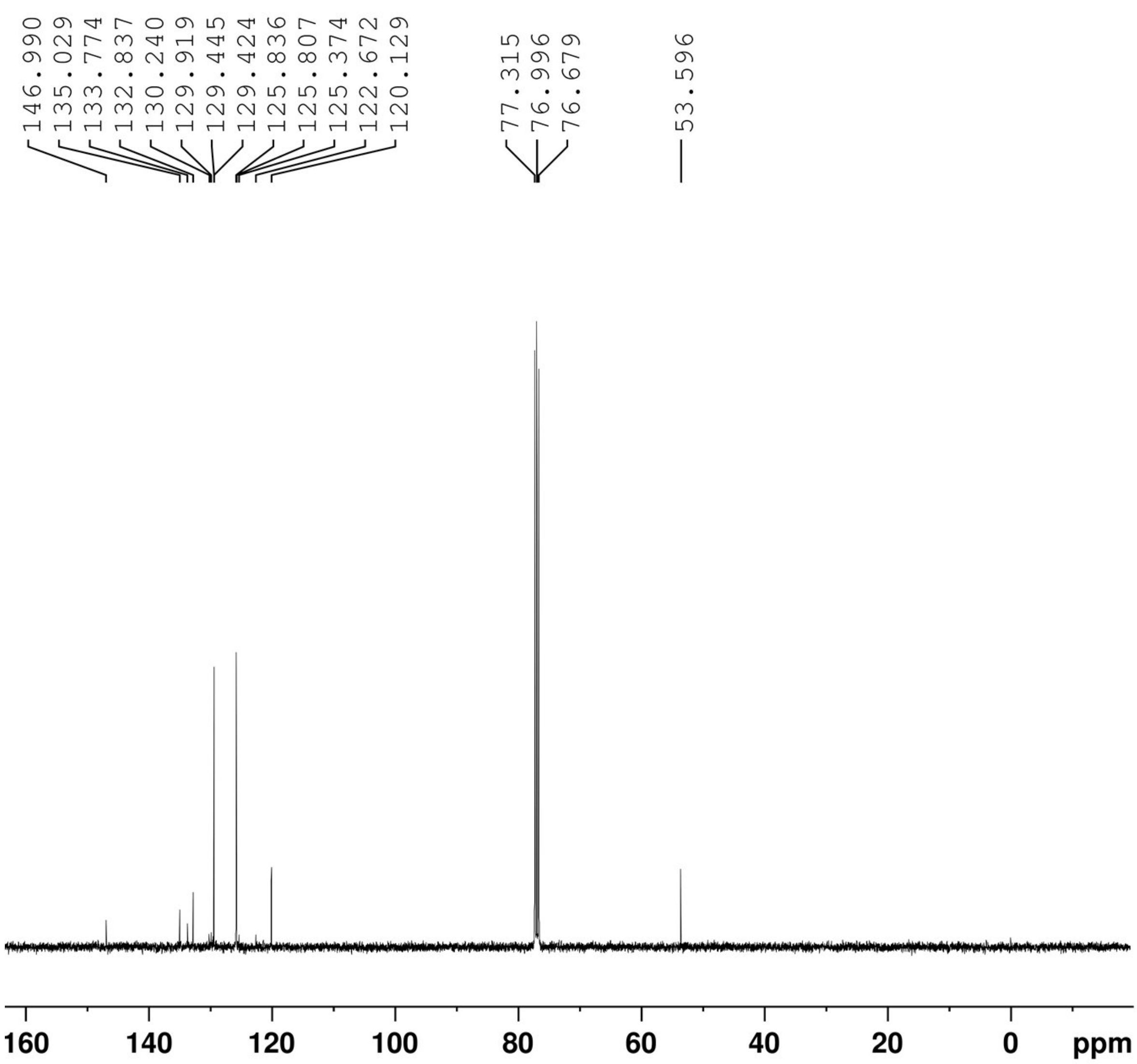

Figure S41. ^{13}C NMR (100 MHz, $CDCl_3$) spectra of compound **p**

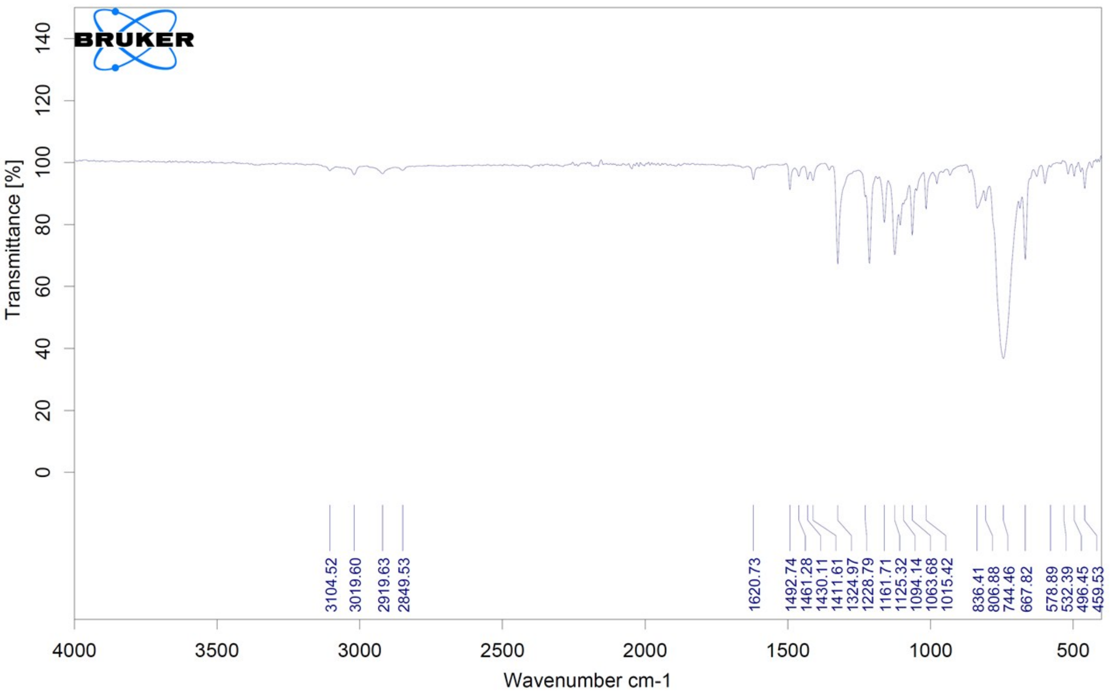

Figure S42. IR spectra of compound **p**

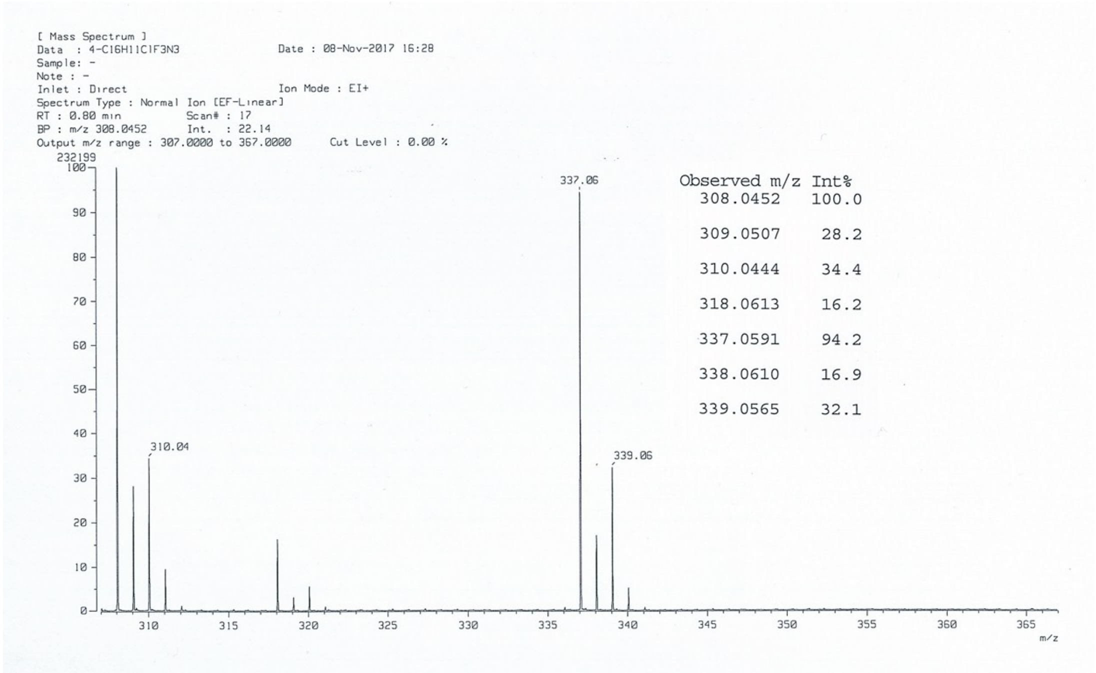

Figure S43. Mass spectra of compound **p**

1-(3-Methoxybenzyl)-4-(4-(trifluoromethyl)phenyl)-1*H*-1,2,3-triazole (q)

White solid, yield: Cu(I)@IPSi 0.323 g (97%), Cu(II)@IPSi 0.287 g (86%), Cu(I)@pNIPAM-VP 0.307 g (92%), Cu(II)@pNIPAM-VP 0.300 g (90%); R_f = 0.10 (hexane/ethyl acetate = 5:1, v/v); m.p. 116.2-117.4 ^{o}C; ^{1}H NMR (400 MHz, $CDCl_3$): δ 7.91 (d, *J* = 8.0 Hz, 2H), 7.75 (s, 1H), 7.65 (d, *J* = 8.4 Hz, 2H), 7.31 (t, *J* = 8.0 Hz, 1H), 6.91 (d, *J* = 8.3 Hz, 2H), 6.84 (t, *J* = 1.9 Hz, 1H); ^{13}C NMR (100 MHz, $CDCl_3$): δ 160.21, 146.86, 135.82, 133.98, 130.36, 129.97 (q, *J* = 32.6 Hz), 125.83, 125.81 (q, *J* = 3.0 Hz), 124.10 (q, *J* = 270.4 Hz), 120.32, 120.28, 114.30, 113.84, 55.34, 54.33; IR(KBr): 3108, 3016, 2948, 2844, 1614, 1587, 1329, 1164, 1109, 1067, 835, 752 cm^{-1}; HRMS(EI): *m/z* $[M]^+$ for $C_{17}H_{14}F_3N_3O$, calculated: 333.1089, found: 333.1088.

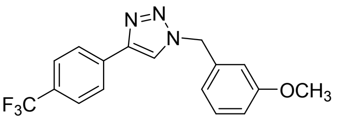

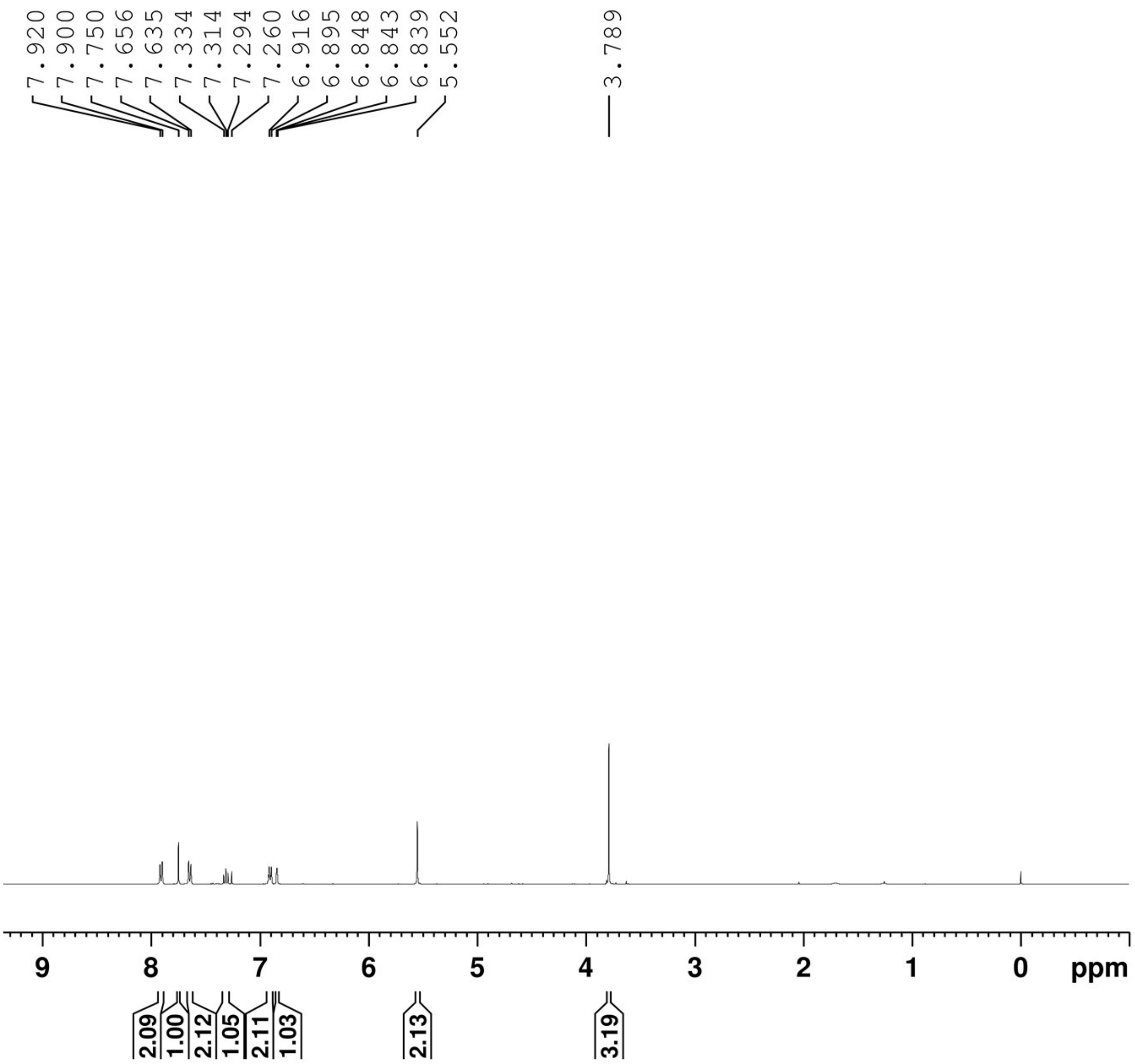

Figure S44. ^{1}H NMR (400 MHz, $CDCl_3$) spectra of compound **q**

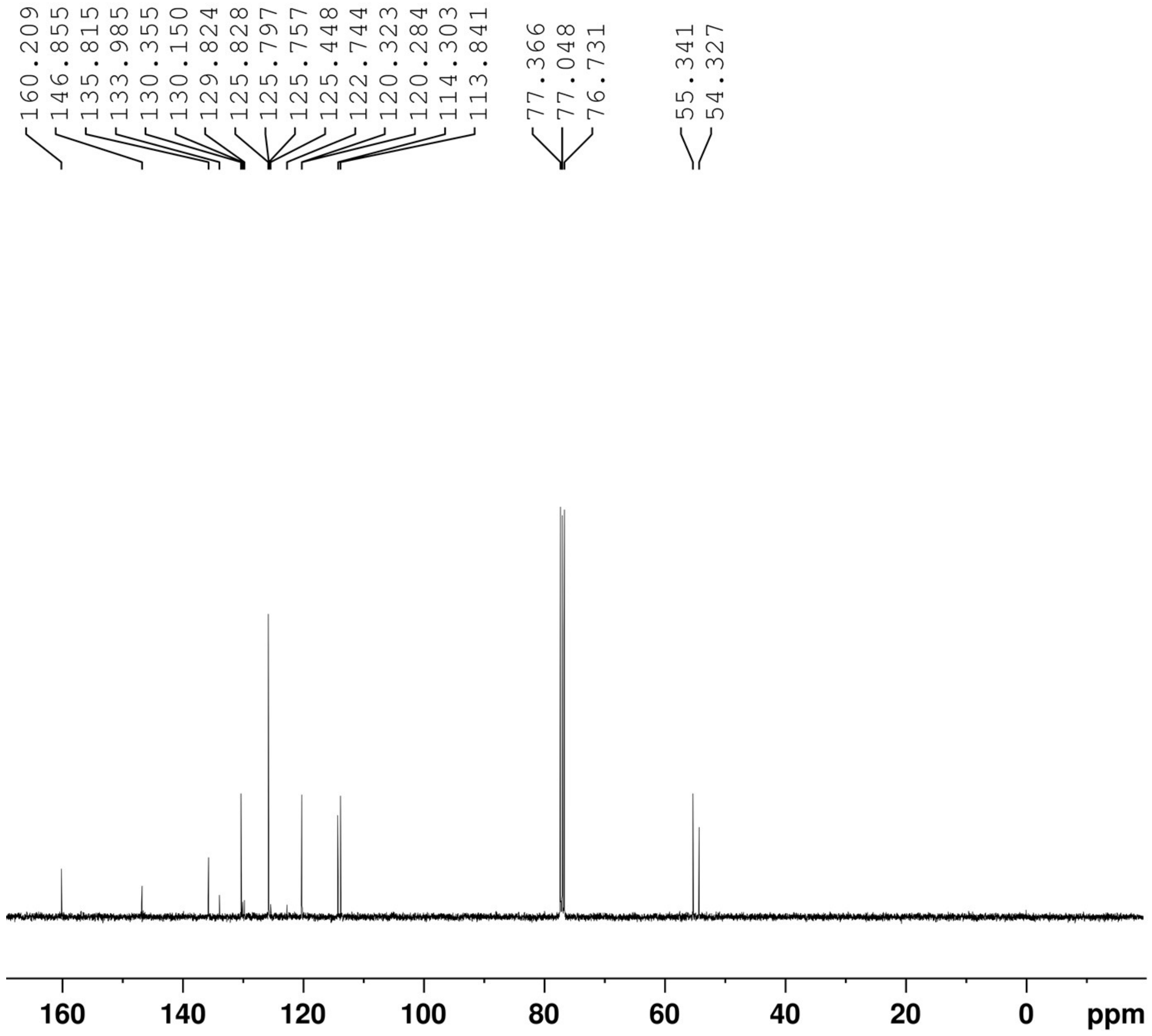

Figure S45. ^{13}C NMR (100 MHz, $CDCl_3$) spectra of compound **q**

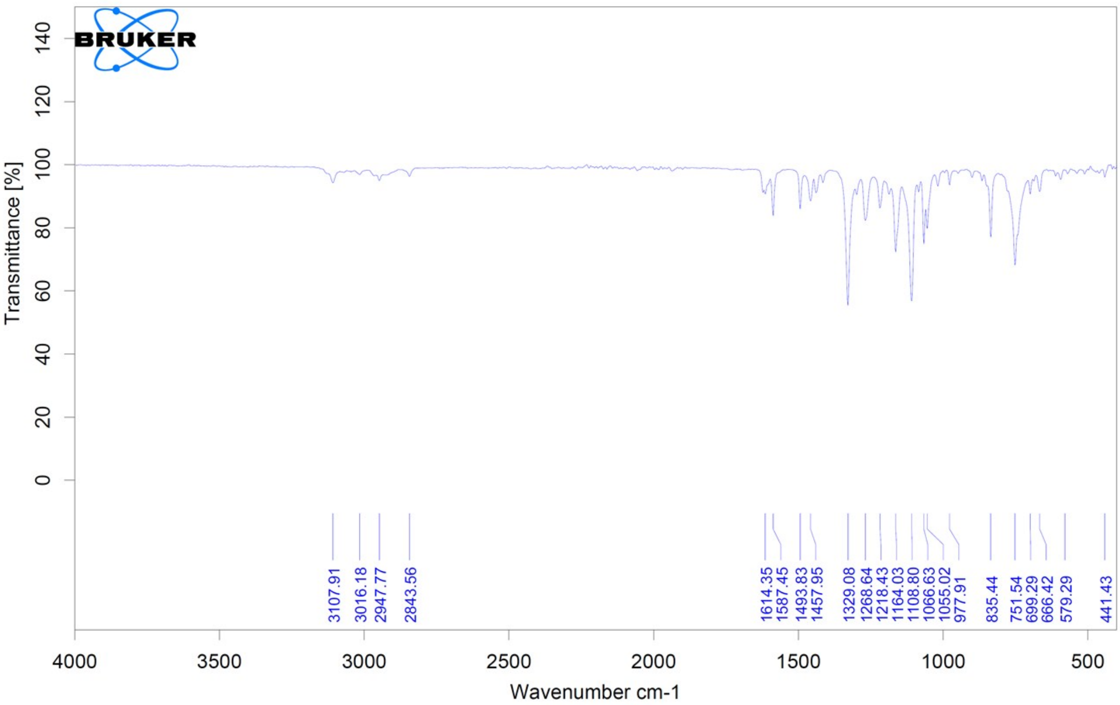

Figure S46. IR spectra of compound **q**

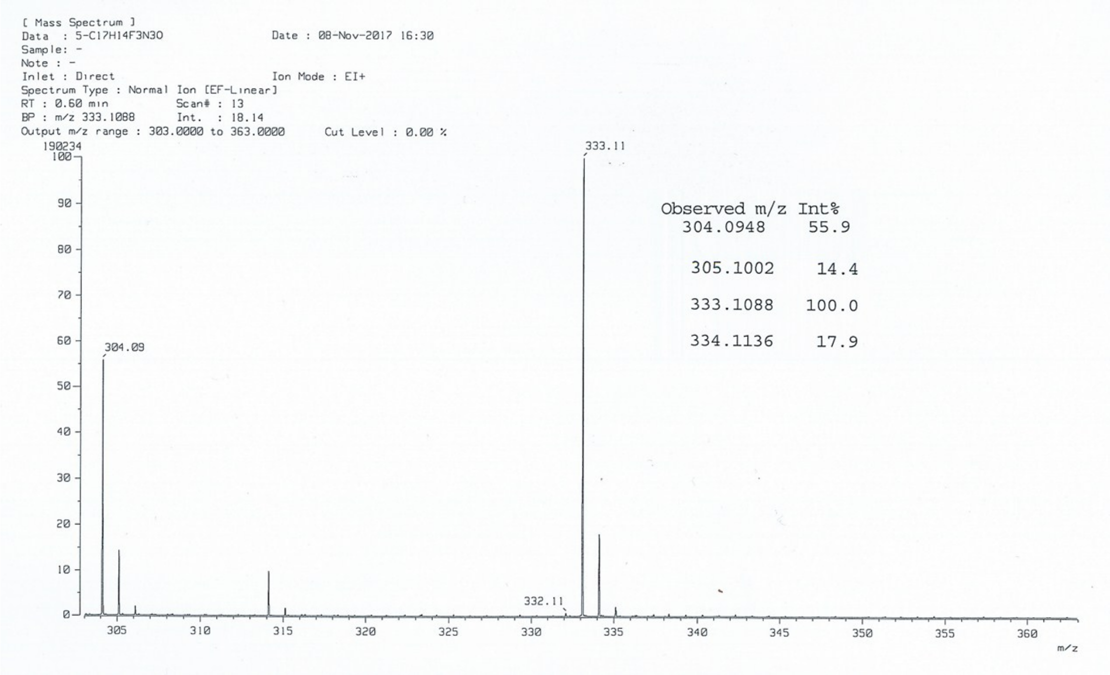

Figure S47. Mass spectra of compound **q**

6. References

[1] H. Sharghi, R. Khalifeh, M. M. Doroodmand, *Adv. Synth. Catal.* **2009**, *351*, 207-218.

[2] Q. Yang, C. Kuang, X. Cheng, Y. Yang, *Synthesis* **2011**, *2011*, 2907-2912.

[3] K. Kamata, Y. Nakagawa, K. Yamaguchi, N. Mizuno, *J. Am .Chem. Soc.* **2008**, *130*, 15304-15310.

[4] F. Wang, H. Fu, Y. Jiang, Y. Zhao, *Green Chem.* **2008**, *10*. 452-456.

[5] K. Asano, S. Matsubara, *Org. Lett.* **2010**, *12*, 4988-4991.

[6] Z. Jia, K. Wang, T. Li, B. Tan, Y. Gu, *Catal. Sci. Tech.* **2016**, *6*, 4345-4355.

[7] S. Chassaing, A. Sani Souna Sido, A. Alix, M. Kumarraja, P. Pale, J. Sommer, *Chemistry* **2008**, *14*, 6713-6721.

[8] L. Rinaldi, K. Martina, F. Baricco, L. Rotolo, G. Cravotto, *Molecules* **2015**, *20*, 2837-2849.

[9] C. Shao, X. Wang, Q. Zhang, S. Luo, J. Zhao, Y. Hu, *J. Org. Chem.* **2011**, *76*, 6832-6836.

[10] S. S. E. Ghodsinia, B. Akhlaghinia, R. Jahanshahi, *RSC Adva.***2016**, *6*, 63613-63623.

[11] D. Linares, O. Bottzeck, O. Pereira, A. Praud-Tabaries, Y. Blache, *Bioorg. Med. Chem. Lett.* **2011**, *21*, 6751-6755.
